# The first example of the Fischer–Hepp type rearrangement in pyrimidines

**DOI:** 10.3762/bjoc.9.212

**Published:** 2013-09-06

**Authors:** Inga Cikotiene, Mantas Jonusis, Virginija Jakubkiene

**Affiliations:** 1 Department of Organic Chemistry, Faculty of Chemistry, Vilnius University, Naugarduko 24, LT-03225, Vilnius, Lithuania, Fax: +370 5 233 09 87 Email: Inga Cikotiene - inga.cikotiene@chf.vu.lt

**Keywords:** Fischer–Hepp rearrangement, nitrosation, 5-nitrosopyrimidines, nucleophilic substitution, pyrimidinediamines

## Abstract

A *N*-nitroso moiety can be used for the activation of chloropyrimidines toward a nucleophilic substitution reaction with amines. The subsequent treatment of the obtained products with aq H_2_SO_4_ can lead to either *N*-denitrosation to obtain 4,6-pyrimidinediamines or to a Fischer–Hepp type rearrangement to obtain 5-nitroso-4,6-pyrimidinediamines. It was found that the outcome of the reaction strongly depends on the structure of the pyrimidines. Activation of the pyrimidine ring by three groups with a positive mesomeric effect is crucial for the intramolecular nitroso group migration.

## Introduction

**Scheme 1:**
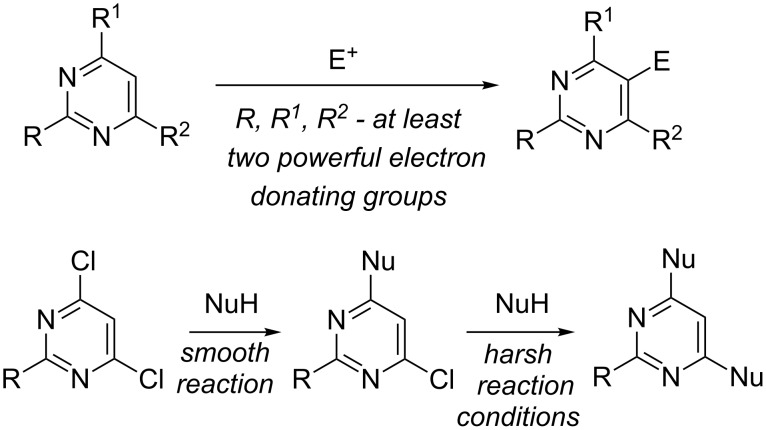
General behavior of electrophilic and nucleophilic substitution reactions of pyrimidines.

The pyrimidine moiety is an important structural motif in natural products and therefore frequently used as a building block for pharmaceutical agents [1–2]. It is well-known that chemical properties of pyrimidines depend on the π-defficient character of this heterocycle. An electrophilic aromatic substitution at the C-5 of a pyrimidine is usually difficult [1,3–5]. However, the presence of two or three activating groups leads to the successful introduction of an electrophile (Scheme 1) [1,6–8]. On the other hand, nucleophilic aromatic substitution reactions of halopyrimidines are smooth and high-yielding, especially when an electron-withdrawing group is present in this heterocycle. It is noteworthy that in the case of non-activated dihalopyrimidines, the first nucleophilic displacement reaction deactivates the pyrimidine core toward subsequent substitution. The usage of very harsh reaction conditions (prolonged heating for hours or days, high pressure or microwave irradiation of the reaction mixtures) is required to carry out the second S_N_Ar reaction (Scheme 1) [9–14].

**Scheme 2:**
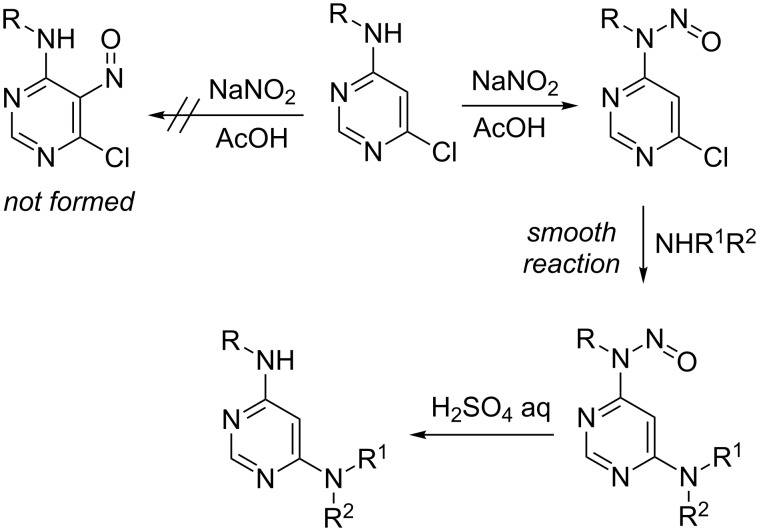
Our previous results.

In 2012 we published a rebuttal to the article about the electrophilic nitrosation of selected pyrimidines [15]. We showed that instead of the previously reported electrophilic attack of the C-5 by NO^+^, the secondary amino substituents in position 4 of the pyrimidine ring underwent *N*-nitrosation reactions (Scheme 2). We also recognized that a 6-chloro substituent is activated by the *N*-nitroso moiety in position 4 of the pyrimidine ring which facilitates nucleophilic substitutions. Moreover, after completion of the substitution reaction, *N*-nitroso moieties can be easily removed by short heating in diluted sulfuric acid [16].

When we fine-tuned our proposed method for the preparation of a bigger variety of pyrimidinediamines [17–27], we observed a unique reactivity of some substrates. It was found that the presence of methylthio, acetamido or morpholino substituents in position 2 of the pyrimidine ring changed the outcome of the reaction. Instead of a denitrosation reaction, selective nitroso group migration to C-5 and the formation of 2,4,6-trisubstituted 5-nitrosopyrimidines took place. It is noteworthy that the resulting 5-nitrosopyrimidines are important intermediates for the preparation of condensed pyrimidine derivatives [27–28], have an interesting crystal structures [29–31], can be useful as bidentate ligands [32–34], and represent a class of biologically active compounds [35–39].

## Results and Discussion

**Scheme 3:**
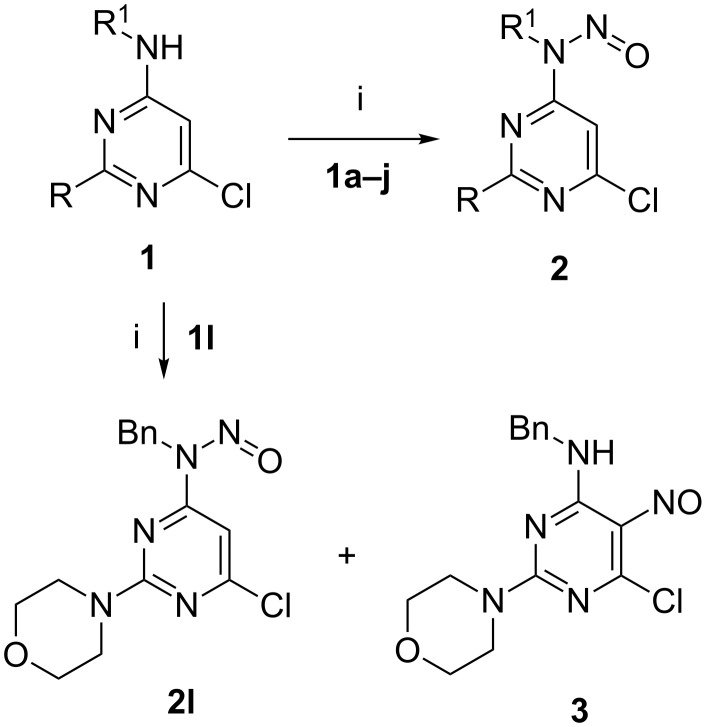
Reagents and conditions: i: NaNO_2_ (1.2 equiv), AcOH, rt; **1a**,**2a**: R = H; R^1^ = Me; **1b**,**2b**: R = H; R^1^ = Bn; **1c**,**2c**: R = H; R^1^ = Bu; **1d**,**2d**: R = Me; R^1^ = Bn; **1e**,**2e**: R = SMe; R^1^ = Bn; **1f**,**2f**: R = SMe; R^1^ = CH_2_(4-MeOC_6_H_4_); **1g**,**2g**: R = SMe; R^1^ = (CH_2_)_2_(4-MeOC_6_H_4_); **1h**,**2h**: R = SMe; R^1^ = Bu; **1i**,**2i**: R = SMe; R^1^ = Ph; **1j**,**2j**: R = NHAc; R^1^ = Bn; **1k**,**2k**: R = NHAc; R^1^ = Bu; **1l**,**2l**: R = N(CH_2_)_4_O; R^1^ = Bn.

The starting compounds **1** were prepared by the reaction of commercially or synthetically available 4,6-dichloropyrimidines with an excess of primary amines in boiling 2-propanol. As it is was shown by us earlier, the starting compounds **1** underwent smooth and high-yielding *N*-nitrosation reactions by using sodium nitrite in acetic acid at room temperature. However, the nitrosation of compound **1l**, bearing a morpholino moiety in position 2 of the pyrimidine ring, was not selective. After the treatment of **1l** with sodium nitrite in acetic acid at room temperature, a mixture of *N*- and *C*-nitrosated compounds **2l** and **3** is formed (Scheme 3). This fact can be explained by the stronger activation of the pyrimidine ring by the tertiary amine moiety (morpholine is a better activator than methyl, methylthio or acetamido groups).

**Scheme 4:**
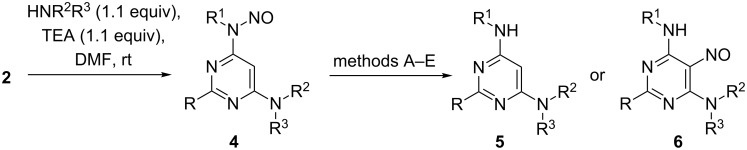
*N*-Denitrosation reaction and intramolecular nitroso group transfer reactions in 6,-*N*-disubstituted-*N*-nitrosopyrimidin-4-amines **4**.

The prepared requisite *N*-nitrosated compounds **2** easily undergo the subsequent nucleophilic substitution reaction with amines. The reactions performed smoothly when mixtures of compounds **2** and an excess of amines were stirred in DMF at room temperature. The substitution products **4** were isolated as yellowish solids (Scheme 4). Then the prepared *N*-nitrosated compounds **4** were tested toward denitrosation conditions. The data of this study are presented in Scheme 4 and Table 1.

**Table 1:** Study on *N*-denitrosation reaction and intramolecular nitroso group transfer reactions in 6,-*N*-disubstituted-*N*-nitrosopyrimidin-4-amines.

Entry	Comp. **4**	R	R^1^	NR^2^R^3^	Method	Product	Yield [%]

1	**4aa**	H	Me	N(CH_2_)_4_	A^a^	**5aa**	69%
2	**4aa**	H	Me	N(CH_2_)_4_	B^b^	**5aa**	97%
3	**4aa**	H	Me	N(CH_2_)_4_	E^c^	slow formation of **5aa**^d^	
4	**4ba**	H	Bn	N(CH_2_)_4_	A	**5ba**	69%
5	**4bb**	H	Bn	N(CH_2_)_4_O	A	**5bb**	92%
6	**4bc**	H	Bn	NHBu	A	**5bc**	54%
7	**4bd**	H	Bn	NHC_6_H_4_-4-OMe	A	**5bd**	56%
8	**4bd**	H	Bn	NHC_6_H_4_-4-OMe	A	**5bd**	75%
9	**4db**	Me	Bn	NEt_2_	A	**5db**	36%
10	**4db**	Me	Bn	NEt_2_	E	slow formation of **5db**^d^	
11	**4ea**	SMe	Bn	N(CH_2_)_5_	A	**5ea, 6ea**	56%^e^
12	**4ea**	SMe	Bn	N(CH_2_)_5_	B	**5ea**	60%
13	**4ea**	SMe	Bn	N(CH_2_)_5_	C^f^	n.r.	–
14	**4ea**	SMe	Bn	N(CH_2_)_5_	D^g^	n.r.	–
15	**4ea**	SMe	Bn	N(CH_2_)_5_	E	**6ea**	94%
16	**4ee**	SMe	Bn	NEt_2_	E	**6ee**	89%

^a^Reaction conditions: 10% aq H_2_SO_4_, 120 °C, 10 min. ^b^Reaction conditions: NH_2_-NH_2_·H_2_O (3 equiv), 10 mol % Pd/C, EtOH, reflux, 2 h. ^c^Reaction conditions: 50% aq H_2_SO_4_, rt, 10 min. ^d^Incomplete conversion of the starting materials. ^e^Overall yield ^f^Reaction conditions: 10% aq H_2_SO_4_, rt, 10 min–1 h. ^g^Reaction conditions: 25% aq H_2_SO_4_, rt, 10 min–1 h.

As it was shown by us earlier, *N*-nitrosated compounds **4**, bearing hydrogen or methyl groups in position 2 of the pyrimidine ring undergo a smooth denitrosation reaction during heating in 10% sulfuric acid at 120 °C (Table 1, entries 1 and 4–9). After the work-up of the reaction mixtures yellowish solids **5** were obtained in good yields. Moreover, the *N*-nitroso moiety can be easily removed by heating of compounds **4** with NH_2_NH_2_·H_2_O in ethanol in the presence of 10% Pd/C (Table 1, entries 2 and 12) under reflux. We became deeply intrigued by the result obtained during heating of the suspension of **4ea**, bearing a methylthio group in position 2 of the pyrimidine ring, in 10% sulfuric acid at 120 °C (Table 1, entry 11). After the classical work-up of the reaction mixture, two products were isolated. One of these products was proved to be *N*-benzyl-2-methylthio-6-(piperidin-1-yl)pyrimidin-4-amine (**5ea**), and the other one *N*-benzyl-2-methylthio-5-nitroso-6-(piperidin-1-yl)pyrimidin-4-amine (**6ea**), representing the product of an unprecedented nitroso group migration to C-5 (Table 1, entry 11). We assumed that at lower temperatures the product of the nitroso group migration could be favoured. However, when the mixture of **4ea** in 10% sulfuric acid was stirred at room temperature, no change of the starting material was observed by TLC (Table 1, entry 13). Increasing the concentration of sulfuric acid to 25% also did not give any satisfactory result (Table 1, entry 14). These facts can be explained by the poor solubility of **4ea** in 10 and 25% aqueous sulfuric acid, so that no reaction took place at room temperature. When we changed the concentration of sulfuric acid to 50% (v/v), the solubility of compound **4ea** at room temperature increased significantly, and after 10 min stirring and a work-up of the reaction mixture pure *N*-benzyl-2-methylthio-5-nitroso-6-(piperidin-1-yl)pyrimidin-4-amine (**6ea**) (Table 1, entry 15) was isolated. Analogously, 5-nitrosopyrimidine **6ee** was prepared (Table 1, entry 16). However, the stirring of the solutions of compounds **4aa** and **4db** in 50% (v/v) sulfuric acid at room temperature led to a slow denitrosation reaction and no rearrangement products were isolated or observed by TLC (Table 1, entries 3 and 10).

**Scheme 5:**
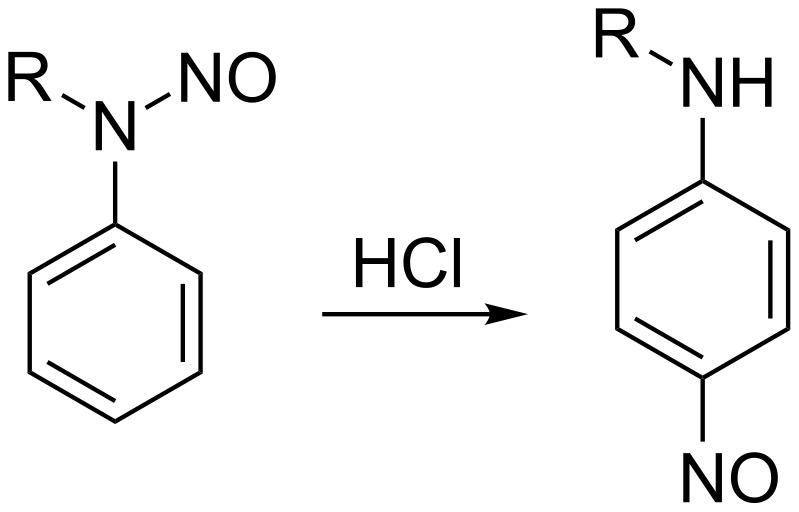
The classical Fischer–Hepp rearrangement*.*

In summary, the outcome of the reaction is determined by the structure of 6,-*N*-disubstituted-*N*-nitrosopyrimidin-4-amines **4**. The presence of a group with a positive mesomeric effect in position 2 of the pirimidine ring is crucial for the migration of the nitroso group to C-5. The migration of a nitroso group in benzene derivatives is a well-known reaction, named Fischer–Hepp rearrangement (Scheme 5) [40–42]. The classical Fischer–Hepp rearrangement takes place when *N*-nitroso secondary anilines are treated with HCl or HBr, other acids give poor results or none at all.

To the best of our knowledge, there are no examples of the migration of a nitroso group in heterocyclic compounds in the scientific literature. Obviously, the reaction we observed has a lot in common with the Fischer–Hepp rearrangement. However, hydrochloric and hydrobromic acids gave worse results in comparison to sulfuric acid. In order to shed some light on the reaction mechanism, we performed the rearrangement reaction of **4ea** in the presence of 20 equivalents of carbamide which is able to react with free NO^+^ ions. We found that the presence of carbamide did not have any effect on the rearrangement process, so we assume that the process is intramolecular and the denitrosation and the C-5 attack steps occurred concurrently in a protonated form of compounds **4**. A more detailed investigation of the mechanism of the reaction will be published in the future.

Next, we tried to apply a one-pot procedure for nucleophilic substitution/rearrangement steps. When the solutions of compounds **2e–l** in DMF were treated with the corresponding amine in the presence of triethylamine at room temperature, a smooth completion of the nucleophilic substitution reactions was observed by TLC. Then the solutions were diluted with 50% (v/v) sulfuric acid, and the resultant yellow colored mixtures were stirred at room temperature for 15 min. After the work-up of the reaction mixtures blue or violet coloured materials were isolated in good yields (Table 2).

**Table 2:** Data on the one-pot nucleophilic substitution/nitroso group transfer reactions in 6-chloro-*N*-disubstituted-*N*-nitrosopyrimidin-4-amines.

Entry	Starting material **2**	Amine R^2^R^3^NH	Product **6**	Yield [%]

	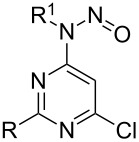		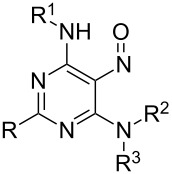	

1	**2e** (R = SMe; R^1^ = Bn)	(CH_2_)_5_NH	**6ea**	94%
2	**2e**	O(CH_2_)_4_NH	**6eb**	84%
3	**2e**	Et_2_NH	**6ec**	89%
4	**2e**	BnNH_2_	**6ed**	48%
5	**2e**	*n*-BuNH_2_	**6ee**	89%
6	**2e**	*c*-HexNH_2_	**6ef**	87%
7	**2e**	MeNH_2_	**6eg**	97%
8	**2e**	*c*-PrNH_2_	**6eh**	36%
9	**2e**	Ph(CH_2_)_2_NH_2_	**6ei**	89%
10	**2f** (R = SMe; R^1^ = CH_2_(4-MeOC_6_H_4_)	(CH_2_)_5_NH	**6fa**	65%
11	**2f**	iPrNH_2_	**6fb**	65%
12	**2f**	BnNH_2_	**6fc**	66%
13	**2g** (R = SMe; R^1^ = (CH_2_)_2_(4-MeOC_6_H_4_)	O(CH_2_)_4_NH	**6ga**	80%
14	**2h** (R = SMe; R^1^ = *n*-Bu)	(CH_2_)_5_NH	**6ha**	96%
15	**2h**	O(CH_2_)_4_NH	**6hb**	82%
16	**2h**	Et_2_NH	**6hc**	90%
17	**2h**	*n*-BuNH_2_	**6hd**	96%
18	**2h**	*c*-HexNH_2_	**6he**	91%
19	**2h**	Ph(CH_2_)_2_NH_2_	**6hf**	92%
20	**2i** (R = SMe; R^1^ = Ph)	O(CH_2_)_4_NH	**6ia**	42%
21	**2j** (R = NHAc; R^1^ = Bn)	O(CH_2_)_4_NH	**6ja**	78%
22	**2j**	(CH_2_)_5_NH	**6jb**	69%
23	**2k** (R = NHAc; R^1^ = *n*-Bu)	O(CH_2_)_4_NH	**6ka**	79%
24	**2l** (R = N(CH_2_)_4_O; R^1^ = Bn)	O(CH_2_)_4_NH	**6la**	72%

**Scheme 6:**
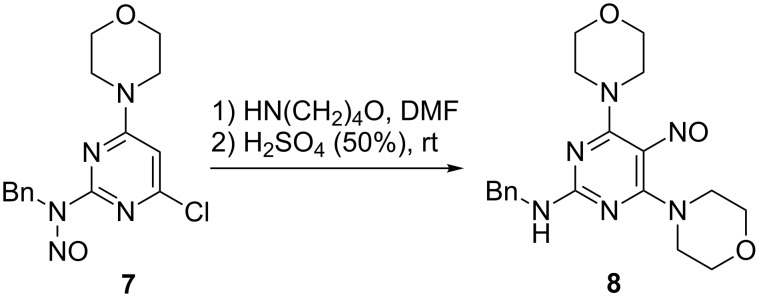
One-pot nucleophilic substitution and nitroso group migration in *N*-benzyl-4-chloro-6-morpholino*-N*-nitrosopyrimidin-2-amine (**7**).

Moreover, it was found, that the migration of the nitroso group is also possible from the second position of the pyrimidine ring. Thus, the treatment of *N*-benzyl-4-chloro-6-morpholino*-N*-nitrosopyrimidin-2-amine (**7**) with morpholine in DMF, and the subsequent quenching of the reaction mixture with 50% (v/v) sulfuric acid gave the desired *N*-benzyl-4,6-dimorpholino-5-nitrosopyrimidin-2-amine (**8**) in 92% yield (Scheme 6).

In conclusion, the ability of *N*-substituted-6-chloropyrimidin-4-amines to undergo a *N*-nitrosation reaction facilitates a very useful and convenient synthetic possibility for the preparation of pyrimidinediamines [16] or 5-nitroso-4,6-pyrimidinediamines. An *N*-nitroso moiety, which assisted the nucleophilic displacement reaction, does not require harsh reaction conditions and it is a high-yielding process. The subsequent migration reaction of the nitroso group in activated 6,-*N*-disubstituted-*N*-nitrosopyrimidin-4-amines occurs smoothly and allows for the preparation of chemically interesting 2,4,6-trisubstituted 5-nitrosopyrimidines.

## Conclusion

The first example of an intramolecular nitrosogroup transfer in 6,-*N*-disubstituted-*N*-nitrosopyrimidin-4-amines was demonstrated. It was found that the presence of at least three electron donating groups is crucial for the transfer of the nitroso group to C-5 of the pyrimidine ring. This method represents a novel, simple and high-yielding preparative way of synthetically useful trisubstituted 5-nitrosopyrimidines. The detailed investigation of the mechanism of this transformation is in progress and will be published in due course.

## Experimental

IR spectra were run in KBr discs on a Perkin-Elmer FT spectrophotometer Spectrum BX II. ^1^H and ^13^C NMR spectra were recorded with a Varian Unity Inova (300 MHz) or a Brucker spectrometer (400 MHz) by using residual solvent peaks as internal standard. HRMS spectra were obtained on a mass spectrometer Dual-ESI Q-TOF 6520 (Agilent Technologies). All reactions and the purity of the synthesized compounds were monitored by TLC with Silica gel 60 F_254_ aluminium plates (Merck). Visualization was accomplished by UV light.

Preparation of the starting materials **1**, *N*-nitrosation and denitrosation reactions were performed according the methods published earlier [15–16].

**Typical one-pot nucleophilic substitution–rearrangement procedure**. A solution of the corresponding 6-chloro-*N*-nitroso-*N*-substitutedpyrimidin-4-amines **2** or *N*-benzyl-4-chloro-6-morpholino*-N*-nitrosopyrimidin-2-amine (**7**) in DMF (0.4 mL) was treated with 2.2 equiv of the corresponding amine. The mixture was stirred at room temperature. After completion of the substitution reaction (10 min–60 h), 50% sulfuric acid (6 mL) was added. The resulting brightly yellow mixture was stirred at room temperature for 15 min. Then the pH of the mixture was carefully adjusted to >7 with solid sodium bicarbonate. The blue-coloured product was extracted with ethyl acetate and purified by recrystallisation or column chromatography (eluting with a mixture of hexane:EtOAc) to give products **6** and **8**.

***N*-Benzyl-2-methylthio-5-nitroso-6-(piperidin-1-yl)pyrimidin-4-amine (6ea)**: Blue solid; yield 94%; mp 127 °C; IR (KBr): ν_max_ = 3442 (NH) cm^−1^; ^1^H NMR (400 MHz, CDCl_3_, 25 °C) δ 1.77 (br. s, 6H, (CH_2_)_3_), 2.49 (s, 3H, SCH_3_), 4.16 (br. s, 4H, N(CH_2_)_2_), 4.73 (d, *J* = 6 Hz, 2H, CH_2_), 7.27–7.34 (m, 5H, ArH), 12.32 (br. s, 1H, NH) ppm; ^13^C NMR (100 Hz, CDCl_3_, 25 °C) δ 14.4 (SCH_3_), 24.5 (CH_2_), 26.7 (CH_2_), 43.8 (NCH_2_Bn), 50.02 (broad, N(CH_2_)_2_), 127.3, 127.6, 128.5, 137.2 (Ar-C), 141.2 (C-5), 147.5, 159.8 (C-4 and C-6), 177.1 (C-2) ppm; HRMS (ES): [M + Na]^+^ calcd for C_17_H_21_N_5_NaOS, 366.1359; found, 366.1359.

***N*-Benzyl-2-methylthio-6-morpholino-5-nitrosopyrimidin-4-amine (6eb)**: Blue solid; yield 84%; mp 101 °C; IR (KBr): ν_max_ = 3179 (NH) cm^−1^; ^1^H NMR (400 MHz, CDCl_3_, 25 °C) δ 2.50 (s, 3H, SCH_3_), 3.85 (t, *J* = 4.8 Hz, 4H, N(CH_2_)_2_), 4.29 (t, *J* = 4.8 Hz, 4H, O(CH_2_)_2_), 4.74 (d, *J* = 5.6 Hz, 2H, CH_2_), 7.28–7.37 (m, 5H, ArH), 12.27 (br. s, 1H, NH) ppm; ^13^C NMR (100 MHz, CDCl_3_, 25 °C) δ 14.6 (SCH_3_), 43.9 (NCH_2_Bn), 49.3 (broad, NCH_2_), 67.2 (OCH_2_), 127.5, 127.6, 128.6, 137.0 (Ar-C), 141.4 (C-5), 147.3, 160.1 (C-4 and C-6), 177.7 (C-2) ppm; HRMS (ES): [M + Na]^+^ calcd for C_16_H_19_N_5_NaO_2_S, 368.1152; found, 368.1149.

***N^4^*-Benzyl-*N^6^,N^6^*-diethyl-2-methylthio-5-nitrosopyrimidine-4,6-diamine (6ec)**: Blue solid; yield 89%; mp 58–60 °C; IR (KBr): ν_max_ = 3207 (NH) cm^−1^; ^1^H NMR (300 MHz, CDCl_3_, 25 °C) δ 1.34 (t, *J* = 6.9 Hz, 6H, (CH_3_)_2_), 2.52 (s, 3H, SCH_3_), 3.91 (q, *J* = 6.9 Hz, 4H, N(CH_2_)_2_), 4.74 (d, *J* = 6 Hz, 2H, CH_2_), 7.29–7.36 (m, 5H, ArH), 12.57 (br. s, 1H, NH) ppm; ^13^C NMR (75 Hz, CDCl_3_, 25 °C) δ 12.6 (broad, CH_3_), 13.9 (broad, CH_3_), 14.4 (SCH_3_), 43.7 (NCH_2_Bn), 46.0 (broad, NCH_2_Me), 47.6 (broad, NCH_2_Me), 127.4, 127.7, 128.5, 137.3 (Ar-C), 141.5 (C-5), 147.7, 159.4 (C-4 and C-6), 176.8 (C-2) ppm; HRMS (ES): [M + H]^+^ calcd for C_16_H_22_N_5_O_2_S, 348.1489; found, 348.1485.

***N^4^,N^6^*-Dibenzyl-2-methylthio-5-nitrosopyrimidine-4,6-diamine (6ed)**: Blue solid; yield 48%; mp 117–119 °C; IR (KBr): ν_max_ = 3335 (NH) cm^−1^; ^1^H NMR (400 MHz, CDCl_3_, 25 °C) δ 2.55 (s, 3H, SCH_3_), 4.75 (d, *J* = 6 Hz, 2H, CH_2_), 4.88 (d, *J* = 6 Hz, 2H, CH_2_), 7.28–7.39 (m, 10H, ArH), 8.17 (br. s, 1H, NH), 11.87 (br. s, 1H, NH) ppm; ^13^C NMR (100 Hz, CDCl_3_, 25 °C) δ 14.7 (SCH_3_), 43.6, 44.9 (NCH_2_Bn), 127.7, 127.8, 127.9, 128.7, 128.8, 136.8, 137.3 (Ar-C), 137.5 (C-5), 146.0, 161.1 (C-4 and C-6), 181.2 (C-2) ppm; HRMS (ES): [M + Na]^+^ calcd for C_19_H_19_N_5_NaOS, 388.1203; found, 388.1206.

***N^4^*-Benzyl-*N^6^*-butyl-2-methylthio-5-nitrosopyrimidine-4,6-diamine (6ee)**: Blue solid; yield 89%; mp 82–84 °C; IR (KBr): ν_max_ 3249 (NH) cm^−1^; NMR spectra contain signals of two rotamers. ^1^H NMR (400 MHz, CDCl_3_, 25 °C) δ = 0.94–0.99 (m, 3H, CH_3_), 1.37–1.49 (m, 2H, CH_2_), 1.56–1.72 (m, 2H, CH_2_), 2.53 (s, 3H, SCH_3_), 3.51–3.55 and 3.64–3.69 (2 m, 2H, NCH_2_), 4.72 (d, *J* = 5.6 Hz, 2H, NCH_2_Bn), 4.84 (d, *J* = 6 Hz, 2H, NCH_2_Bn), 7.28–7.38 (m, 5H, ArH), 7.92 and 8.20 (2 br. s, 1H, NH), 11.66 and 11.92 (2 br. s, 1H, NH) ppm; ^13^C NMR (100 Hz, CDCl_3_, 25 °C) δ 13.5, 13.6 (SCH_3_), 14.51, 14.53 (CH_2_), 19.8, 19.9 (CH_2_), 30.8, 31.2 (CH_2_), 39.2, 40.6 (NCH_2_Pr), 43.3, 44.6 (NCH_2_Bn), 127.4, 127.5, 127.6, 127.7, 128.5, 136.7, 137.19 (Ar-C), 137.24, 137.3 (C-5), 145.9, 146.2, 160.9 (C-4 and C-6), 180.7, 180.9 (C-2) ppm; HRMS (ES): [M + Na]^+^ calcd for C_16_H_21_N_5_NaOS, 354.1359; found, 354.1365.

## Supporting Information

File 1 Detailed data of all new materials, photos of the rearrangement process, copies of the NMR spectra of final compounds **6** and **8**.
