# Supplementary material for: The first example of the Fischer–Hepp type rearrangement in pyrimidines
Source: Beilstein J Org Chem. 2013 Sep 6;9:1819–25. doi: 10.3762/bjoc.9.212 (PMC3778328; doi:10.3762/bjoc.9.212)
Supplement: File 1 — Detailed data of all new materials, photos of the rearrangement process, copies of the NMR spectra of final compounds 6 and 8. [file Beilstein_J_Org_Chem-09-1819-s001.pdf]

Supporting Information

for

The first example of the Fischer–Hepp type rearrangement in pyrimidines

Inga Cikotiene*, Mantas Jonusis and Virginija Jakubkiene

Address: Department of Organic Chemistry, Faculty of Chemistry, Vilnius University, Naugarduko 24, LT-03225, Vilnius, Lithuania, Fax: +370 5 233 09 87

Email: Inga Cikotiene - inga.cikotiene@chf.vu.lt

Detailed data of all new materials, photos of rearrangement process, copies of NMR spectra of final compounds 6 and 8.

Data for compounds **1a,b** [1], **1c** [2], **2a,b** [3], **2c** [4], **2d** [4], **4aa** [3], **4ba** [3], **4bb** [3], **4bc** [4], **4bd** [4], **5aa** [4], **5ba** [4], **5bb** [4], **5bc** [4], **5bd** [4] have been published previously.

N-Benzyl-6-chloro-2-methylpyrimidin-4-amine **(1d):** yellowish wax; yield 94%. IR (KBr): ν_{max} = 3324 (NH) cm^{-1}. ^{1}H NMR (300 MHz, $CDCl_3$, 25 °C): δ = 2.51 (3H, s, CH_3), 4.45 – 4.60 (2H, m, NH$\underline{CH_2}$Ph), 5.42 (1H, br. s, NH), 6.21 (1H, s, C(5)-$\underline{H}$), 7.26 – 7.50 (5H, m, ArH) ppm. ^{13}C NMR (75 Hz, $CDCl_3$, 25 °C): δ = 26.02 (CH_3), 45.81 (NH$\underline{CH_2}$Ph), 98.84 (C-5), 127.58, 128.09, 129.16, 131.03 (Ar-C), 160.06, 163.68 (C-4 and C-6), 168.57 (C-2) ppm. HRMS (ESI): M+Na^+, found 256.0620. $C_{12}H_{12}{}^{35}ClN_3Na$ requires 256.0617.

N-Benzyl-6-chloro-2-methylthiopyrimidin-4-amine **(1e):** white solid; yield 96%, mp 72–74 ^{o}C. IR (KBr): ν_{max} = 3253 (NH) cm^{-1}. ^{1}H NMR (400 MHz, $CDCl_3$, 25 °C): δ = 2.48 (3H, s, SCH_3), 4.56 (2H, br. s, NH$\underline{CH_2}$Ph), 5.77 (1H, br. s, NH), 6.07 (1H, s, C(5)-$\underline{H}$), 7.29 – 7.38 (5H, m, ArH) ppm. ^{13}C NMR (100 Hz, $CDCl_3$, 25 °C): δ = 14.14 (SCH_3), 45.27 (NH$\underline{CH_2}$Bn), 98.58 (broad, C-5), 127.25, 127.56, 128.67, 137.42 (Ar-C), 158.79, 162.54 (C-4 and C-6), 172.08 (C-2) ppm. HRMS (ESI): M+Na^+, found 288.0334. $C_{12}H_{12}{}^{35}ClN_3NaS$ requires 288,0338.

6-Chloro-N-(4-methoxybenzyl)-2-methylthiopyrimidin-4-amine **(1f):** white solid; yield 96%, mp 85–87 ^{o}C (decomp). IR (KBr): ν_{max} = 3252 (NH) cm^{-1}. ^{1}H NMR (300 MHz, $CDCl_3$, 25 °C): δ = 2.48 (3H, s, SCH_3), 3.80 (3H, s, OCH_3), 4.44 (2H, br. s, NH$\underline{CH_2}$Ar), 4.98 (1H, br. s, NH), 6.03 (1H, s, C(5)-$\underline{H}$), 6.87 (2H, d, J = 9.0 Hz, ArH), 7.21 (2H, d, J = 9.0 Hz, ArH) ppm. ^{13}C NMR (75 Hz, $CDCl_3$, 25 °C): δ = 14.05 (SCH_3), 44.96 (NH$\underline{CH_2}$Ar), 55.27 (OCH_3), 97.71 (broad, C-5), 114.18, 128.78, 137.42, 139.00 (Ar-C), 159.16, 161.54 (C-4 and C-6), 171.92 (C-2) ppm. HRMS (ESI): M+Na^+, found 318.0448. $C_{13}H_{14}{}^{35}ClN_3NaOS$ requires 318.0444.

***6-Chloro-N-(4-methoxyphenethyl)-2-methylthiopyrimidin-4-amine* (1g):** white solid; yield 79%, mp 89–91 ^{o}C. IR (KBr): ν_{max} = 3250 (NH) cm^{-1}. ^{1}H NMR (300 MHz, $CDCl_3$, 25 °C): δ = 2.50 (3H, s, SCH_3), 2.84 (3H, t, *J* = 6.0 Hz, CH_2Ar), 3.55 (2H, br. s, NHCH_2), 3.79 (3H, s, OCH_3), 5.28 (1H, br. s, NH), 6.00 (1H, s, C(5)-H), 6.85 (2H, d, *J* = 9.0 Hz, ArH), 7.11 (2H, d, *J* = 9.0 Hz, ArH) ppm. ^{13}C NMR (75 Hz, $CDCl_3$, 25 °C): δ = 14.07 (SCH_3), 34.36 (CH_2Ar), 45.78 (NHCH_2), 55.26 (OCH_3), 98.44 (broad, C-5), 114.16, 129.66, 137.41, 139.05 (Ar-C), 158.63, 162.30 (C-4 and C-6), 172.00 (C-2) ppm. HRMS (ESI): M+Na^{+}, found 332.0609. $C_{14}H_{16}{}^{35}ClN_3NaOS$ requires 332.0600.

***N-Butyl-6-chloro-2-methylthiopyrimidin-4-amine* (1h):** white solid; yield 99%, mp 63–64 ^{o}C (petroleum). IR (KBr): ν_{max} = 3255 (NH) cm^{-1}. ^{1}H NMR (400 MHz, $CDCl_3$, 25 °C): δ = 0.95 (3H, t, *J* = 7.6 Hz, CH_3), 1.35 – 1.44 (2H, m, CH_2), 1.55 – 1.62 (2H, m, CH_2), 2.49 (3H, s, SCH_3), 3.29 (2H, br. s, NHCH_2), 5.28 (1H, br. s, NH), 6.04 (1H, s, C(5)-H) ppm. ^{13}C NMR (100 Hz, $CDCl_3$, 25 °C): δ = 13.63 (CH_3), 13.95 (SCH_3), 19.92 (CH_2), 31.10 (CH_2), 41.20 (NHCH_2), 99.89 (broad, C-5), 156.68, 162.62 (C-4 and C-6), 171.91 (C-2) ppm. HRMS (ESI): M+Na^{+}, found 254.0487. $C_9H_{14}{}^{35}ClN_3NaS$ requires 254.0489.

***6-Chloro-2-methylthio-N-phenylpyrimidin-4-amine* (1i):** yellow solid; yield 88%, mp 97–98 ^{o}C (2-propanol). IR (KBr): ν_{max} = 3276 (NH) cm^{-1}. ^{1}H NMR (400 MHz, $CDCl_3$, 25 °C): δ = 2.51 (3H, s, SCH_3), 6.37 (1H, s, C(5)-H), 7.04 – 7.24 (2H, m, ArH), 7.28 – 7.42 (4H, m and br. s, ArH, NH) ppm. ^{13}C NMR (100 Hz, $CDCl_3$, 25 °C): δ = 14.22 (SCH_3), 98.37 (broad, C-5), 122.83, 125.52, 129.54, 137.37 (Ar-C), 159.87, 161.42 (C-4 and C-6), 172.79 (C-2) ppm. HRMS (ESI): M+Na^{+}, found 274.0188. $C_{11}H_{10}{}^{35}ClN_3NaS$ requires 274.0182.

***N-(4-(Benzylamino)-6-chloropyrimidin-2-yl)acetamide* (1j):** white solid; yield 97%, mp 152–154 ^{o}C. IR (KBr): ν_{max} = 3341, 3250 (NH), 1676 (CO) cm^{-1}. ^{1}H NMR (300 MHz, DMSO, 25 °C): δ = 2.19 (3H, s, $COCH_3$), 4.53 (2H, br. s, NHCH_2Ph), 6.22 (1H, s, C(5)-H), 7.20 – 7.29 (5H, m, ArH), 7.99 (1H, t, *J* = 6.0 Hz, NHCH_2), 9.85 (1H, br. s, NH), ppm. ^{13}C NMR (75 Hz, DMSO, 25 °C): δ = 24.96 (CH_3), 43.91 (NHCH_2Ph), 98.57 (C-5), 126.88, 127.33, 128.24, 138.84 (Ar-C), 157.02, 157.41, 163.71 (C-4, C-6 and C-2), 170.27 (CO) ppm. HRMS (ESI): M+H^{+}, found 277.0851. $C_{13}H_{14}{}^{35}ClN_4O$ requires 277.0852.

***N-(4-(Butylamino)-6-chloropyrimidin-2-yl)acetamide* (1k):** white solid; yield 81%, mp 118–119 ^{o}C. IR (KBr): ν_{max} = 3343, 3255 (NH), 1675 (CO) cm^{-1}. ^{1}H NMR (300 MHz, $CDCl_3$, 25 °C): δ = 0.94 (3H, t, *J* = 6.0 Hz, CH_3), 1.35 – 1.43 (2H, m, CH_2), 1.56 – 1.66 (2H, m, CH_2), 2.54 (3H, s, $COCH_3$), 3.15 (2H, br. s, NHCH_2), 6.05 (1H, s, C(5)-H), 7.53 (1H, br. s, NH), 9.68 (1H, br. s, NH) ppm. ^{13}C NMR (75 Hz, $CDCl_3$, 25 °C): δ = 13.69 (CH_3), 20.07 (CH_2), 25.46 (CH_3CO), 30.79 (CH_2), 41.72 (NHCH_2), 95.45 (C-5), 164.13, 164.27, 166.36 (C-4, C-6 and C-2), 173.96 (CO) ppm. HRMS (ESI): M+H^{+}, found 243.1007. $C_{10}H_{16}{}^{35}ClN_4O$ requires 243.1012.

***N-Benzyl-6-chloro-2-morpholin-4-ylpyrimidin-4-amine* (1l)**: white solid; yield 85%; mp 154–156 ^{o}C. IR (KBr): ν_{max} = 3321 (NH) cm^{-1}. ^{1}H NMR (400 MHz, $CDCl_3$, 25 °C): δ = 3.71 – 3.76 (m, 8H, $N(CH_2)_4O$), 4.53 (2H, s, NHCH_2Ph), 5.08 (1H, br. s, NH), 5.75 (1H, s, C(5)-H), 7.30 – 7.39 (5H, m, ArH) ppm. ^{13}C NMR (100 MHz, $CDCl_3$, 25 °C): δ = 44.30 (NCH_2), 45.41 (NCH_2), 66.82 (OCH_2), 92.13 (C-5), 127.52, 127.65, 128.81, 138.35 (Ar-C), 159.90, 161.10 (C-4 and C-6), 163.58 (C-2) ppm. HRMS (ESI): M+H^{+}, found 305.1168. $C_{15}H_{18}ClN_4O$ requires 305.1164.

***N-Benzyl-6-chloro-2-methylthio-N-nitrosopyrimidin-4-amine* (2e):** yellow solid; yield 99%, mp 89–90 ^{o}C. ^{1}H NMR (400 MHz, $CDCl_3$, 25 °C): δ = 2.58 (3H, s, SCH_3), 5.33 (2H, br. s, NCH_2Ph), 7.19 – 7.36 (5H, m, ArH), 7.72 (1H, s, C(5)-H) ppm. ^{13}C NMR (100 Hz, $CDCl_3$, 25 °C): δ = 14.46 (SCH_3), 43.57 (NCH_2Ph), 102.54 (C-5), 127.90, 128.15, 128.67, 134.32 (Ar-

C), 161.57, 161.70 (C-4 and C-6), 173.61 (C-2) ppm. HRMS (ESI): $M+H^+$, found 295.0422. $C_{12}H_{12}{}^{35}ClN_4OS$ requires 295.0420.

6-Chloro-N-(4-methoxybenzyl)-2-methylthio-N-nitrosopyrimidin-4-amine **(2f):** yellowish solid; yield 81%, mp 88–89 ^oC. ^1H NMR (300 MHz, DMSO, 25 °C): δ = 2.57 (3H, s, SCH_3), 3.71 (3H, s, OCH_3), 5.22 (2H, s, N$\underline{CH_2}$Ar), 6.86 (2H, d, *J* = 9.0 Hz, ArH), 7.14 (2H, d, *J* = 9.0 Hz, ArH), 7.88 (1H, s, C(5)-$\underline{H}$), ppm. ^{13}C NMR (75 Hz, DMSO, 25 °C): δ = 13.91 (SCH_3), 43.24 (N$\underline{CH_2}$Ar), 55.04 (OCH_3), 102.92 (C-5), 113.92, 126.40, 129.00 (2C) (Ar-C), 158.59, 160.71 (C-4 and C-6), 172.61 (C-2) ppm. HRMS (ESI): $M+Na^+$, found 347.0348. $C_{13}H_{13}{}^{35}ClN_4NaO_2S$ requires 347.0345.

6-Chloro-N-(4-methoxyphenethyl)-2-methylthio-N-nitrosopyrimidin-4-amine **(2g):** yellowish solid; yield 89%, mp 92–93 ^oC. ^1H NMR (300 MHz, $CDCl_3$, 25 °C): δ = 2.60 (3H, s, SCH_3), 2.67 – 2.72 (3H, m, $\underline{CH_2}$Ar), 3.78 (3H, s, OCH_3), 4.26 – 4.31 (2H, m, N$\underline{CH_2}$), 6.82 (2H, d, *J* = 9.0 Hz, ArH), 7.10 (2H, d, *J* = 9.0 Hz, ArH), 7.62 (1H, s, C(5)-$\underline{H}$), ppm. ^{13}C NMR (75 Hz, $CDCl_3$, 25 °C): δ = 14.38 (SCH_3), 31.76 (CH_2Ar), 41.84 (N$\underline{CH_2}$), 55.24 (OCH_3), 102.24 (C-5), 114.00, 129.41, 129.68, 129.69 (Ar-C), 158.46, 161.49 (C-4 and C-6), 173.49 (C-2) ppm. HRMS (ESI): $M+Na^+$, found 361.0506. $C_{14}H_{15}{}^{35}ClN_4NaO_2S$ requires 361.0502.

N-Butyl-6-chloro-2-methylthio-N-nitrosopyrimidin-4-amine **(2h):** yellow powder; yield 96%, mp 45–46 ^oC. ^1H NMR (400 MHz, $CDCl_3$, 25 °C): δ = 0.89 (3H, t, *J* = 7.3 Hz, CH_3), 1.21 – 1.30 (2H, m, CH_2), 1.40 – 1.47 (2H, m, CH_2), 2.56 (3H, s, SCH_3), 4.08 (2H, t, *J* = 7.3 Hz, N$\underline{CH_2}$), 7.62 (1H, s, C(5)-$\underline{H}$) ppm. ^{13}C NMR (100 Hz, $CDCl_3$, 25 °C): δ = 13.61 (CH_3), 14.39 (SCH_3), 20.23 (CH_2), 28.64 (CH_2), 40.35 (NH$\underline{CH_2}$), 102.31 (C-5), 161.50, 161.81 (C-4 and C-6), 173.52 (C-2) ppm. HRMS (ESI): $M+Na^+$, found 283.0386. $C_9H_{13}{}^{35}ClN_4NaOS$ requires 283.0391.

6-Chloro-2-methylthio-N-phenyl-N-nitrosopyrimidin-4-amine **(2i):** yellow solid; yield 66%, mp 104–106 ^oC (2-propanol). ^1H NMR (400 MHz, $CDCl_3$, 25 °C): δ = 2.27 (3H, s, SCH_3), 6.95 – 7.05 (2H, m, ArH), 7.49 – 7.58 (3H, m, ArH), 7.64 (1H, s, C(5)-$\underline{H}$) ppm. ^{13}C NMR (100 Hz, $CDCl_3$, 25 °C): δ = 14.12 (SCH_3), 102.37 (C-5), 128.18, 129.52, 130.05, 133.43 (Ar-C), 161.58, 162.14 (C-4 and C-6), 173.66 (C-2) ppm. HRMS (ESI): $M+Na^+$, found 303.0087. $C_{11}H_9{}^{35}ClN_4NaOS$ requires 303.0083.

N-(4-(Benzyl(nitroso)amino)-6-chloropyrimidin-2-yl)acetamide **(2j):** white solid; yield 60%, mp 129–130 ^oC. IR (KBr): ν_{max} = 3050 (NH), 1659 (CO) cm^{-1}. ^1H NMR (300 MHz, DMSO, 25 °C): δ = 2.50 (3H, s, $COCH_3$), 5.32 (2H, s, N$\underline{CH_2}$Ph), 7.21 – 7.28 (5H, m, ArH), 7.75 (1H, s, C(5)-$\underline{H}$), 8.30 (1H, br. s, NH) ppm. ^{13}C NMR (75 Hz, DMSO, 25 °C): δ = 25.31 (CH_3), 43.60 (N$\underline{CH_2}$Ph), 102.08 (C-5), 127.84, 127.88, 128.66, 133.91 (Ar-C), 156.49, 162.41, 163.02 (C-4, C-6 and C-2), 170.52 (CO) ppm. HRMS (ESI): $M+H^+$, found 328.0579. $C_{13}H_{12}{}^{35}ClN_5O_2$ requires 328.0577.

N-(4-(Butyl(nitroso)amino)-6-chloropyrimidin-2-yl)acetamide **(2k):** white solid; yield 77%, mp 111–113 ^oC. IR (KBr): ν_{max} = 3062 (NH), 1651 (CO) cm^{-1}. ^1H NMR (300 MHz, $CDCl_3$, 25 °C): δ = 0.94 (3H, t, *J* = 7.2 Hz, CH_3), 1.24 – 1.37 (2H, m, CH_2), 1.43 – 1.53 (2H, m, CH_2), 2.59 (3H, s, $COCH_3$), 4.10 (2H, t, *J* = 7.8 Hz, N$\underline{CH_2}$), 7.70 (1H, s, C(5)-$\underline{H}$), 8.21 (1H, br. s, NH) ppm. ^{13}C NMR (75 Hz, $CDCl_3$, 25 °C): δ = 13.57 (CH_3), 20.21 (CH_2), 25.30 ($\underline{CH_3}$CO), 28.57 (CH_2), 40.49 (N$\underline{CH_2}$), 101.84 (C-5), 156.58, 162.27, 163.28 (C-4, C-6 and C-2), 170.85 (CO) ppm. HRMS (ESI): $M+Na^+$, found 294.0728. $C_{10}H_{14}{}^{35}ClN_5NaO_2$ requires 294.0722.

N-Benzyl-6-chloro-2-morpholin-4-yl-N-nitrosopyrimidin-4-amine **(2l)**: yellowish solid; yield 37%; mp 130–132 ^oC. ^1H NMR (400 MHz, $CDCl_3$, 25 °C): δ = 3.73 – 3.78 (4H, m, $N(CH_2)_2$), 3.81 – 3.87 (4H, m, $O(CH_2)_2$), 5.28 (2H, s, NH$\underline{CH_2}$Ph), 7.16 – 7.20 (2H, s and m,

C(5)-H, ArH), 7.25 – 7.32 (4H, m, ArH) ppm. ^{13}C NMR (100 MHz, $CDCl_3$, 25 °C): δ = 43.51 (NCH_2), 44.40 (NCH_2), 66.62 (OCH_2), 96.03 (C-5), 127.68, 127.71, 128.62, 134.80 (Ar-C), 160.42, 162.34 (C-4 and C-6), 162.44 (C-2) ppm. HRMS (ESI): [M + Na]$^+$, found 356.0880. $C_{15}H_{16}ClN_5NaO_2$ requires 356.0885.

***N*-Benzyl-6-chloro-2-morpholin-4-yl-5-nitrosopyrimidin-4-amine** (**3**): blue solid; yield 35%; mp 139–141 °C (decomp.). IR (KBr): ν_{max} = 3237 (NH) cm^{-1}. ^{1}H NMR (400 MHz, $(CD_3)_2CO$, 25 °C): δ = 3.70 – 3.73 (2H, m, NCH_2), 3.75 – 3.78 (2H, m, NCH_2), 3.95 – 3.98 (2H, m, OCH_2), 4.02 – 4.05 (2H, m, OCH_2), 4.73 – 4.75 (2H, m, NH$\underline{CH_2}$Ph), 7.16 – 7.20 (2H, m, C(5)-H, ArH), 7.26 – 7.40 (4H, m, ArH), 11.03 (1H, br. s, NH) ppm. ^{13}C NMR (100 MHz, $CDCl_3$, 25 °C): δ = 43.30 (NCH_2), 45.24 (NCH_2), 66.03 (OCH_2), 66.32 (OCH_2), 127.31, 127.84, 128.53, 138.04 (Ar-C), 144.06 (C-5), 149.03, 157.80 (C-4 and C-6), 172.32 (C-2) ppm. HRMS (ES): [M + Na]$^+$, found 356.0889. $C_{15}H_{16}ClN_5NaO_2$ requires 356.0885.

***N*-Benzyl-*N*-(6-diethylamino-2-methylpyrimidin-4-yl)nitrous amide** (**4db**): yellowish wax; yield 69%.^{1}H NMR (400 MHz, $CDCl_3$, 25 °C): δ = 1.19 (6H, t, J = 7.1 Hz. ($CH_2\underline{CH_3}$)$_2$), 2.53 (3H, s, CH_3), 3.39 – 3.70 (4H, m, ($\underline{CH_2}CH_3$)$_2$), 5.38 (2H, s, NCH_2), 6.82 (1H, s, C(5)-H), 7.17 – 7.34 (5H, m, ArH) ppm. ^{13}C NMR (100 Hz, $CDCl_3$, 25 °C): δ = 12.0 (CH_3), 26.35 (CH_3), 42.34 (NCH_2), 43.66 (NCH_2), 85.67 (C-5), 127.38, 128.37, 128.60, 135.48 (Ar-C), 160.05, 162.20 (C-4 and C-6), 167.16 (C-2) ppm. HRMS (ES): M+Na+, found 322.1639. $C_{16}H_{21}N_5NaO$ requires 322.1644.

***N*-Benzyl-*N*-(2-methylthio-6-(piperidin-1-yl)pyrimidin-4-yl)nitrous amide** (**4ea**): yellowish solid; yield 95%, mp 127–128 °C. ^{1}H NMR (400 MHz, $CDCl_3$, 25 °C): δ = 1.61 – 1.69 (4H, m, $2CH_2$), 1.69 – 1.77 (2H, m, CH_2), 2.53 (3H, s, SCH_3), 3.60 – 3.75 (4H, m, N($\underline{CH_2}$)$_2$), 5.37 (2H, s, NCH_2Ph), 6.86 (1H, s, C(5)-H), 7.21 – 7.32 (5H, m, ArH) ppm. ^{13}C NMR (100 Hz, $CDCl_3$, 25 °C): δ = 14.24 (SCH_3), 24.63 (CH_2), 25.58 (CH_2), 43.75 (NCH_2), 45.47 (NCH_2), 83.65 (C-5), 127.44, 128.35, 128.42, 135.27 (Ar-C), 160.08, 162.28 (C-4 and C-6), 171.03 (C-2) ppm. HRMS (ES): M+Na+, found 366.1361. $C_{17}H_{21}N_5NaOS$ requires 366.1359.

***N*-Benzyl-*N*-6-diethylamino-2-methylpyrimidin-4-amine** (**5db**): white solid; yield 36%, mp 121–123 °C (hexane). IR (KBr): ν_{max} = 3234 (NH) cm^{-1}. ^{1}H NMR (300 MHz, $CDCl_3$, 25 °C): δ = 1.23 (6H, t, *J* = 7.9 Hz. ($CH_2\underline{CH_3}$)$_2$), 2.57 (3H, s, CH_3), 3.58 (4H, br. s, (N$\underline{CH_2}$)$_2$), 5.44 (2H, s, NCH_2), 6.85 (1H, s, C(5)-H), 7.24 – 7.35 (5H, m, ArH) ppm. ^{13}C NMR (75 Hz, $CDCl_3$, 25 °C): δ = 13.08 (CH_3), 26.30 (CH_3), 42.05 (NCH_2), 46.35 (NCH_2), 80.06 (C-5), 127.42, 128.90, 138.88, 141.16 (Ar-C), 162.04, 163.17 (C-4 and C-6), 166.46 (C-2) ppm. HRMS (ES): M+H+, found 271.1928. $C_{16}H_{23}N_4$ requires 271.1923.

***N*-Benzyl-2-methylthio-6-(piperidin-1-yl)pyrimidin-4-amine** (**5ea**): white solid; yield 96%, mp 134–136 °C (ethanol). IR (KBr): ν_{max} = 3257 (NH) cm^{-1}. ^{1}H NMR (400 MHz, DMSO, 25 °C): δ = 1.37 – 1.50 (4H, m, $2CH_2$), 1.51 – 1.61 (2H, m, CH_2), 2.32 (3H, s, SCH_3), 3.40 – 3.44 (4H, m, N($\underline{CH_2}$)$_2$), 4.41 (2H, d, *J* = 5.4 Hz, NH$\underline{CH_2}$Ph), 5.36 (1H, s, C(5)-H), 7.16 – 7.35 (5H, m, ArH) ppm. ^{13}C NMR (100 Hz, DMSO, 25 °C): δ = 13.64 (SCH_3), 21.68 (CH_2), 25.41 (CH_2), 44.19 (NCH_2), 45.05 (NCH_2), 78.01 (C-5), 127.08, 127.66, 128.67, 140.67 (Ar-C), 161.96, 163.36 (C-4 and C-6), 168.98 (C-2) ppm. HRMS (ES): M+H+, found 315.1642. $C_{17}H_{23}N_4S$ requires 315.1643.

***N*^{4}-Benzyl-*N*^{6}-cyclohexyl-2-methylthio-5-nitrosopyrimidine-4,6-diamine** (**6ef**): blue solid; yield 87%; mp 128–130 °C. IR (KBr): ν_{max} = 3318 (NH) cm^{-1}. NMR spectra contain signals of two rotamers.^{1}H NMR (400 MHz, $CDCl_3$, 25 °C): δ = 1.27 – 1.47 (6H, m, (CH_2)$_3$), 1.60 – 1.65, 1.75 – 1.82, 1.94 – 1.98 and 2.06 – 2.11 (4H, 4m, CH($\underline{CH_2}$)$_2$), 2.54 (3H, s, SCH_3), 2.06 – 2.11 (4H, 4m, CH($\underline{CH_2}$)$_2$), 4.16 – 4.26 (1H, m, $\underline{CH}$(CH_2)$_2$), 4.73 (2H, d, *J* = 6 Hz, NCH_2Bn), 4.85 (2H, d, *J* = 6 Hz, NCH_2Bn), 7.28 – 7.40 (5H, m, ArH), 7.82 and 11.68 (1H, 2d, *J* = 8 Hz, $\underline{NH}$CH), 8.13 and 11.94 (1H, 2 t, *J* = 6 Hz, $\underline{NH}CH_2$) ppm. ^{13}C NMR (100 Hz, $CDCl_3$, 25 °C):

δ = 14.60, 14.63 (SCH_3), 24.30, 24.61, 25.34, 32.22, 32.71 ($(CH_2)_3$), 43.50, 44.82 (<u>CH</u>(CH_2)$_2$), 48.47, 49.76 (N<u>CH</u>$_2$Bn), 127.59, 127.65, 127.70, 127.83, 128.65, 128.67, 126.84, 137.18, (Ar-C), 137.21, 137.32 (C-5), 145.28, 146.23, 160.14, 161.12 (C-4 and C-6), 180.86, 181.12 (C-2) ppm. HRMS (ES): M+Na+, found 346.1672. $C_{18}H_{23}N_5NaOS$ requires 346.1672.

***N⁴*-Benzyl-*N⁶*-methyl-2-methylthio-5-nitrosopyrimidine-4,6-diamine** (**6eg**): blue solid; yield 97%; mp 152–154 ^oC. IR (KBr): ν_{max} = 3284 (NH) cm^{-1}. NMR spectra contain signals of two rotamers. ^1H NMR (400 MHz, $CDCl_3$, 25 °C): δ = 2.56 and 2.57 (3H, 2s, SCH_3), 3.08 and 3.22 (3H, 2d, *J* = 5.2 Hz, N<u>CH</u>$_3$), 4.76 and 4.87 (2H, 2d, *J* = 6 Hz, N<u>CH</u>$_2$Bn), 7.28 – 7.41 (5H, m, ArH), 7.96 and 8.170 (1H, 2 br. s, NH), 11.50 and 11.83 (1H, 2 br. s, NH) ppm. ^{13}C NMR (100 Hz, $CDCl_3$, 25 °C): δ = 14.79 (SCH_3), 26.48, 27.98 (N<u>CH</u>$_3$), 43.80, 45.05 (N<u>CH</u>$_2$Bn), 127.82, 127.85, 127.95, 128.81, 128.83, 136.77 137.26, (Ar-C), 137.32, 137.39 (C-5), 146.28, 147.30, 160.96, 161.58 (C-4 and C-6), 181.45, 181.59 (C-2) ppm. HRMS (ES): M+Na+, found 312.0893. $C_{13}H_{15}N_5NaOS$ requires 312.0890.

***N⁴*-Benzyl-*N⁶*-cyclopropyl-2-methylthio-5-nitrosopyrimidine-4,6-diamine** (**6eh**): blue solid; yield 36%; mp 142–144 ^oC. IR (KBr): ν_{max} = 3320 (NH) cm^{-1}. NMR spectra contain signals of two rotamers. ^1H NMR (300 MHz, $CDCl_3$, 25 °C): δ = 0.70 – 0.76 and 0.90 – 0.99 (4H, 2m, CH(<u>CH</u>$_2$)$_2$), 2.58, 2.59 (3H, 2s, SCH_3), 3.71 – 3.79 (1H, m, <u>CH</u>(CH_2)$_2$), 4.76 and 4.87 (2H, 2d, *J* = 6 Hz, NCH_2Bn), 7.32 – 7.40 (5H, m, ArH), 7.84 and 8.11 (1H, 2 br. s, NH), 11.61 and 11.85 (1H, 2 br. s, NH) ppm. ^{13}C NMR (75 Hz, $CDCl_3$, 25 °C): δ = 7.31, 7.52 (CH_2)$_2$), 15.05 (SCH_3), 23.26, 24.47 (<u>CH</u>(CH_2)$_2$), 43.86, 45.20 (N<u>CH</u>$_2$Bn), 127.96, 128.05, 128.18, 129.02, 129.04, 137.12, (Ar-C), 137.58, 137.74 (C-5), 146.21, 147.28, 161.32, 162.75 (C-4 and C-6), 181.48, 181.65 (C-2) ppm. HRMS (ES): MH+, found 316.1239. $C_{15}H_{18}N_5OS$ requires 316.1227.

***N⁴*-Benzyl-2-methylthio-5-nitroso-*N⁶*-phenethylpyrimidine-4,6-diamine** (**6ei**): blue solid; yield 89%; mp 84–86 ^oC. IR (KBr): ν_{max} = 3326 (NH) cm^{-1}. NMR spectra contains signals of two rotamers. ^1H NMR (400 MHz, $CDCl_3$, 25 °C): δ = 2.57 (3H, s, SCH_3), 2.93 and 3.03 (2H, 2t, J = 7.2 Hz, CH_2), 3.79 and 3.95 (2H, 2m, NCH_2), 4.75 and 4.86 (2H, 2d, *J* = 6 Hz and *J* = 5.6 Hz NCH_2Bn), 7.24 – 7.39 (10H, m, ArH), 7.97 and 8.19 (1H, 2 br. s, NH), 11.64 and 11.88 (1H, 2 br. s, NH) ppm. ^{13}C NMR (100 Hz, $CDCl_3$, 25 °C): δ = 14.69 (SCH_3), 35.25, 35.54 (CH_2), 41.27, 42.27 (NCH_2), 43.51, 44.92 (N<u>CH</u>$_2$Bn), 126.59, 126.54, 127.61, 127.66, 127.70, 127.82, 128.60, 128.63, 128.66, 128.71, 136.77, 137.27, 137.35 (Ar-C), 138.20, 138.25 (C-5), 146.04, 146.16, 160.99, 161.09 (C-4 and C-6), 180.97, 181.10 (C-2) ppm. HRMS (ES): M+Na+, found 402.1354. $C_{20}H_{21}N_5NaOS$ requires 402.1359.

***N*-(4-Methoxybenzyl)-2-methylthio-5-nitroso-6-(piperidin-1-yl)pyrimidin-4-amine** (**6fa**): blue wax; yield 65%. IR (KBr): ν_{max} = 3321 (NH) cm^{-1}. ^1H NMR (400 MHz, $CDCl_3$, 25 °C): δ = 1.76 (6H, br. s (CH_2)$_3$), 2.51 (3H, s, SCH_3), 3.79 (3H, s, OCH_3), 4.15 (4H, br. s, N(CH_2)$_2$), 4.65 (2H, d, *J* = 6 Hz, $NHCH_2$), 6.86 (2H, d, J = 8.4 Hz, ArH), 7.24 (2H, d, J = 8.4 Hz, ArH), 12.25 (1H, br. s, NH) ppm. ^{13}C NMR (100 Hz, $CDCl_3$, 25 °C): δ = 14.46 (SCH_3), 24.51 (CH_2), 26.70 (CH_2), 43.25 (N<u>CH</u>$_2$Bn), 49.70 (broad, N(<u>CH</u>$_2$)$_2$), 55.14 (OCH_3), 113.90, 129.03, 129.26, 141.23 (Ar-C), 147.38 (C-5), 158.88, 159.85 (C-4 and C-6), 176.99 (C-2) ppm. HRMS (ES): MH+, found 374.1645. $C_{18}H_{24}N_5O_2S$ requires 374.1638.

***N⁴*-Isopropyl-*N⁶*-(4-methoxybenzyl)-2-methylthio-5-nitrosopyrimidine-4,6-diamine** (**6fb**): blue solid; yield 65%, mp 86–88 ^oC. IR (KBr): ν_{max} = 3322 (NH) cm^{-1}. NMR spectra contain signals of two rotamers. ^1H NMR (300 MHz, $CDCl_3$, 25 °C): δ = 1.28 and 1.35 (6H, 2d, J = 6.6 Hz, (CH_3)$_2$), 2.56 and 2.57 (3H, 2s, SCH_3), 3.82 (3H, s, OCH_3), 4.43 – 4.64 (1H, m, NH<u>CH</u>), 4.68 and 4.78 (2H, 2d, *J* = 6 Hz, $NHCH_2$), 6.87 - 6.91 (2H, m, ArH), 7.24 and 7.32 (2H, 2d, J = 9 Hz, ArH), 7.76 and 8.11 (1H, 2br. s, NH), 11.50 and 11.80 (1H, br. s, NH) ppm. ^{13}C NMR (75 Hz, $CDCl_3$, 25 °C): δ = 14.14, 14.67 (SCH_3), 22.29, 22.54 (CH_3), 42.09,

43.14, 43.15, 44.44 (NCH, N<u>CH</u>$_2$), 55.24 (OCH$_3$), 114.10, 128.78, 129.18, 129.32, 136.86 (Ar-C), 145.45, 146.16 (C-5), 159.13, 159.18, 160.05, 160.77 (C-4 and C-6), 181.14, 181.72 (C-2) ppm. HRMS (ES): MH+, found 348.1489. $C_{16}H_{22}N_5O_2S$ requires 348.1483.

N^4-Benzyl-N^6-(4-methoxybenzyl)-2-methylthio-5-nitrosopyrimidine-4,6-diamine (**6fc**): blue solid; yield 66%; mp 113–114 ^{o}C. IR (KBr): ν_{max} = 3302 (NH) cm^{-1}. NMR spectra contain signals of two rotamers. ^{1}H NMR (400 MHz, CDCl$_3$, 25 °C): δ = 2.56 and 2.57 (3H, 2s, SCH$_3$), 3.82 (3H, s, OCH$_3$), 4.68, 4.75, 4.80 and 4.87 (4H, 4d, *J* = 5.6; 6; 5.6; 6 Hz, 2NHCH$_2$), 6.88 - 6.91 (2H, m, ArH), 7.25 (1H, d, *J* = 8.8 Hz, ArH), 7.32 – 7.39 (6H, m, ArH), 8.10 and 8.16 (1H, 2t, *J* = 5.6 Hz, NH), 11.80 and 11.88 (1H, 2t, *J* = 6 Hz, NH) ppm. ^{13}C NMR (100 Hz, CDCl$_3$, 25 °C): δ = 14.73 (SCH$_3$), 43.07, 43.56, 44.44, 44.90 (N<u>CH</u>$_2$), 55.25 (OCH$_3$), 114.11, 127.65, 127.73, 127.85, 128.70, 128.72, 128.83, 129.16, 129.27, 129.30, 136.81, 137.28 (Ar-C), 145.83, 146.01 (C-5), 159.12, 159.18, 160.93, 161.13 (C-4 and C-6), 181.13, 181.16 (C-2) ppm. HRMS (ES): MH+, found 396.1489. $C_{20}H_{22}N_5O_2S$ requires 396.1484.

N-(4-Methoxybphenethyl)-2-methylthio-6-morpholino-5-nitrosopyrimidin-4-amine (**6ga**): blue solid; yield 80%; mp 109–110 ^{o}C (2-PrOH). IR (KBr): ν_{max} = 3109 (NH) cm^{-1}. ^{1}H NMR (300 MHz, CDCl$_3$, 25 °C): δ = 2.51 (3H, s, SCH$_3$), 2.86 (2H, t, *J* = 7.2 Hz, CH$_2$), 3.71 – 3.77 (2H, m, CH$_2$), 3.79 (3H, s, OCH$_3$), 3.83 (4H, t, *J* = 4.5 Hz, N(CH$_2$)$_2$), 4.27 (4H, t, *J* = 4.5 Hz, O(CH$_2$)$_2$), 6.90 (2H, d, *J* = 8.5 Hz, ArH), 7.14 (2H, d, *J* = 8.5 Hz, ArH), 11.90 (1H, br. s, NH) ppm. ^{13}C NMR (75 Hz, CDCl$_3$, 25 °C): δ = 14.38 (SCH$_3$), 34.03(CH$_2$), 41.89 (CH$_2$), 48.00 (broad, N(<u>CH</u>$_2$)$_2$)54.97 (OCH$_3$), 67.00 (O(<u>CH</u>$_2$)$_2$), 113.81, 129.40, 130.04, 140.50 (Ar-C), 147.39 (C-5), 158.11, 159.59 (C-4 and C-6), 177.68 (C-2) ppm. HRMS (ES): MH+, found 390.1594. $C_{18}H_{24}N_5O_3S$ requires 390.1596.

N-Butyl-2-methylthio-5-nitroso-6-(piperidin-1-yl)pyrimidin-4-amine (**6ha**): blue solid; yield 96%; mp 54–56 ^{o}C. IR (KBr): ν_{max} = 3150 (NH) cm^{-1}. ^{1}H NMR (400 MHz, CDCl$_3$, 25 °C): δ = 0.93 (3H, t, *J* = 7.3 Hz, CH$_3$), 1.33 – 1.42 (2H, m, CH$_2$), 1.55 – 1.62 (2H, m, CH$_2$), 1.70 (6H, br. s (CH$_2$)$_3$), 2.48 (3H, s, SCH$_3$), 3.47 – 3.52 (2H, m, NH<u>CH</u>$_2$), 4.13 (4H, br. s, N(CH$_2$)$_2$), 12.05 (1H, br. s, NH) ppm. ^{13}C NMR (100 Hz, CDCl$_3$, 25 °C): δ = 13.77, 14.57 (SCH$_3$ and CH$_3$) 20.21, 24.68, 26.84, 31.04 (CH$_2$, CH), 39.77 (N<u>CH</u>$_2$Bn), 50.31 (broad, N(<u>CH</u>$_2$)$_2$), 141.25 (C-5), 148.01, 160.01 (C-4 and C-6), 177.11 (C-2) ppm. HRMS (ES): M+Na+, found 332.1509. $C_{14}H_{23}N_5NaOS$ requires 332.1516.

N-Butyl-2-methylthio-5-nitroso-6-morpholinopyrimidin-4-amine (**6hb**): blue solid; yield 82%; mp 60–62 ^{o}C. IR (KBr): ν_{max} = 3443 (NH) cm^{-1}. ^{1}H NMR (400 MHz, CDCl$_3$, 25 °C): δ = 0.93 (3H, t, *J* = 7.2 Hz, CH$_3$), 1.34 – 1.43 (2H, m, CH$_2$), 1.55 – 1.63 (2H, m, CH$_2$), 2.48 (3H, s, SCH$_3$), 3.48 – 3.53 (2H, m, NH<u>CH</u>$_2$), 3.81 – 3.83 (4H, m, N(CH$_2$)$_2$), 4.24 – 4.27 (4H, m, O(CH$_2$)$_2$), 12.00 (1H, br. s, NH) ppm. ^{13}C NMR (100 Hz, CDCl$_3$, 25 °C): δ = 13.73, 14.57 (SCH$_3$ and CH$_3$) 20.19 (CH$_2$), 31.00 (CH$_2$), 39.82 (CH$_2$), 49.44 (broad, N(<u>CH</u>$_2$)$_2$), 67.32 (O(<u>CH</u>$_2$)$_2$), 141.45 (C-5), 147.69, 160.19 (C-4 and C-6), 177.53 (C-2) ppm. HRMS (ES): M+Na+, found 334.1309. $C_{13}H_{21}N_5NaO_2S$ requires 334.1308.

N^4-Butyl-N^6,N^6-diethyl-2-methylthio-5-nitrosopyrimidine-4,6-diamine (**6hc**): blue wax; yield 90%. IR (KBr): ν_{max} = 3325 (NH) cm^{-1}. ^{1}H NMR (400 MHz, CDCl$_3$, 25 °C): δ = 0.91 (3H, td, *J* = 7.3, 1.9 Hz, CH$_3$), 1.28 (3H, br. t, CH$_3$), 1.34 – 1.40 (2H, m, CH$_2$), 1.53 – 1.61 (2H, m, CH$_2$), 2.48 (3H, s, SCH$_3$), 3.45 – 3.50 (2H, m, NH<u>CH</u>$_2$), 3.82 – 3.87 (4H, m., N(CH$_2$)$_2$), 12.29 (1H, br. s, NH) ppm. ^{13}C NMR (100 Hz, CDCl$_3$, 25 °C): δ = 13.75, 14.41 (SCH$_3$ and CH$_3$) 20.20 (CH$_3$), 31.03, 39.64, (CH$_2$), 46.07 and 47.76 (broad, N(<u>CH</u>$_2$)$_2$), 141.57 (C-5), 148.12, 159.49 (C-4 and C-6), 176.75 (C-2) ppm. HRMS (ES): MH+, found 298.1700. $C_{13}H_{24}N_5OS$ requires 298.1696.

N^4^,N^6^-Dibutyl-2-methylthio-5-nitrosopyrimidine-4,6-diamine **(6hd)**: blue solid; yield 96%, mp 57–59 °C.. IR (KBr): ν_{max} = 3330 (NH) cm^{-1}. ^1H NMR (400 MHz, $CDCl_3$, 25 °C): δ = 0.89 – 0.96 (6H, m, $2CH_3$), 1.33 – 1.41 (4H, m, CH_2), 1.52 – 1.57 and 1.62 – 1.68 (4H, 2m, CH_2), 2.49 (3H, br. s, SCH_3), 3.46 – 3.52 and 3.59 – 3.64 (4H, 2m, $NH\underline{CH_2}$), 7.84 (1H, br. s, NH), 11.67 (1H, br. s, NH) ppm. ^{13}C NMR (100 Hz, $CDCl_3$, 25 °C): δ = 13.70, 13.75, 14.65 (SCH_3 and $2CH_3$) 20.05, 20.13, 31.03, 31.39 (CH_2), 39.38 and 40.72 ($2N\underline{CH_2}$), 137.34 (C-5), 146.54, 161.14 (C-4 and C-6), 180.89 (C-2) ppm. HRMS (ES): M+Na+, found 320.1514. $C_{13}H_{23}N_5NaOS$ requires 320.1516.

N^4^-Butyl-N^6^-cyclohexyl-2-methylthio-5-nitrosopyrimidine-4,6-diamine **(6he)**: blue solid; yield 91%, mp 67–68 °C. IR (KBr): ν_{max} = 3239 (NH) cm^{-1}. NMR spectra contain signals of two rotamers. ^1H NMR (400 MHz, $CDCl_3$, 25 °C): δ = 0.94 and 0.96 (3H, 2t, J = 7.2 Hz, CH_3), 1.23 – 1.46 (7H, m, CH_2), 1.55 – 1.68 (3H, m, CH_2), 1.73 – 1.81 (2H, m, CH_2), 1.92 – 1.95 and 2.05 – 2.09 (4H, 2m, CH_2), 2.53 (3H, s, SCH_3) 3.50 – 3.55 and 3.62 – 3.67 (2H, 2m, CH_2), 4.14 – 4.26 (1H, m, $NH\underline{CH}$), 7.76 and 7.84 (1H, d and t, *J* = 8 Hz; 4.8 Hz, NH), 11.71 (1H, br. s, NH) ppm. ^{13}C NMR (100 Hz, $CDCl_3$, 25 °C): δ = 13.60, 13.65, 14.55 (SCH_3 and $2CH_3$), 19.96, 20.04, 24.31, 24.62, 25.35, 25.37 (CH, CH_2), 39.30, 40.62, 48.45 and 49.64 ($N\underline{CH_2}$, $NH\underline{CH}$), 137.05, 137.07 (C-5), 145.43, 146.61, 160.11, 161.12 (C-4 and C-6), 180.84, 180.89 (C-2) ppm. HRMS (ES): M+Na+, found 346.1672. $C_{15}H_{25}N_5NaS$ requires 346.1672.

N^4^-Butyl-2-methylthio-5-nitroso-N^6^-phenethylpyrimidine-4,6-diamine **(6hf)**: blue solid; yield 92%, mp 65–66 °C. IR (KBr): ν_{max} = 3321 (NH) cm^{-1}. NMR spectra contain signals of two rotamers. ^1H NMR (400 MHz, $CDCl_3$, 25 °C): δ = 0.94 – 1.00 (3H, m, CH_3), 1.36 – 1.48 (2H, m, CH_2), 1.57 – 1.50 (2H, m, CH_2), 2.56 (3H, s, SCH_3) 2.92 and 3.01 (2H, 2t, *J* = 7.2 Hz, CH_2), 3.53 and 3.66 (2H, 2q, *J* = 6 and 6.4 Hz, $NH\underline{CH_2}$), 3.77 and 3.92 (2H, 2q, *J* = 6.4 Hz, $NH\underline{CH_2}$), 7.22 – 7.36 (5H, m, ArH), 7.88 and 7.94 (1H, 2t, *J* = 5.6 Hz, NH), 11.68 (1H, br. s, NH) ppm. ^{13}C NMR (100 Hz, $CDCl_3$, 25 °C): δ = 13.76, 13.81, 14.76 (SCH_3 and $2CH_3$), 20.09, 20.18, 31.07, 31.43, 35.40, 35.67 (CH_2), 39.47, 40.79, 41.38 and 42.36 ($2N\underline{CH_2}$,), 126.63, 126.69, 126.74, 128.72, 128.74, 128.76, 137.37, 137.40 (Ar-C), 138.38, 138.44 (C-5), 146.46, 146.57, 161.20, 161.22 (C-4 and C-6), 181.00 (C-2) ppm. HRMS (ES): MH+, found 346.1700. $C_{17}H_{24}N_5OS$ requires 346.1696.

N-Phenyl-2-methylthio-6-morpholino-5-nitrosopyrimidin-4-amine **(6ia)**: blue solid; yield 42%; mp 122–124 °C. IR (KBr): ν_{max} = 3443 (NH) cm^{-1}. ^1H NMR (400 MHz, $CDCl_3$, 25 °C): δ = 2.54 (3H, s, SCH_3), 3.88 (4H, t, *J* = 4.4 Hz, $N(CH_2)_2$), 4.32 (4H, t, *J* = 4.4 Hz, $O(CH_2)_2$), 7.20 (1H, tt, J = 7.2, 1.2 Hz, ArH), 7.36 – 7.40 (2H, m, ArH), 7.76 (2H, dd, J = 7.2, 1.2 Hz, ArH), 14.10 (1H, br. s, NH) ppm. ^{13}C NMR (100 Hz, $CDCl_3$, 25 °C): δ = 14.72 (SCH_3), 49.10 (broad, $N\underline{CH_2}$), 67.29 ($O\underline{CH_2}$), 123.80, 125.58, 128.79, 136.68 (Ar-C), 140.94 (C-5), 144.43 160.12 (C-4 and C-6), 178.42 (C-2) ppm. HRMS (ES): M+Na+, found 354.1000. $C_{15}H_{17}N_5NaO_2S$ requires 354.0995.

2-Acetamido-N-benzyl--6-morpholino-5-nitrosopyrimidin-4-amine **(6ja)**: blue solid; yield 78%; mp 148–150 °C. IR (KBr): ν_{max} = 3278 (NH), 1679 (C=O) cm^{-1}. ^1H NMR (400 MHz, $CDCl_3$, 25 °C): δ = 2.48 (3H, s, $COCH_3$), 3.75 – 3.83 (4H, m, $N(CH_2)_2$), 4.17 – 4.23 (4H, m, $O(CH_2)_2$), 4.63 (2H, d, *J* = 6 Hz, CH_2), 7.22 – 7.32 (5H, m, ArH), 8.12 (1H, br. s, NH), 12.31 (1H, br. s, NH) ppm. ^{13}C NMR (100 Hz, $CDCl_3$, 25 °C): δ = 25.89 ($CO\underline{CH_3}$), 44.22 ($N\underline{CH_2}Bn$), 49.15 (broad, $N\underline{CH_2}$), 67.26 ($O\underline{CH_2}$), 127.44, 127.72, 128.80, 136.77 (Ar-C), 141.25 (C-5), 150.31, 158.02, 162.76 (C-4, C-6 and $NH\underline{CO}CH_3$), 172.00 (C-2) ppm. HRMS (ES): MH+, found 357.1673. $C_{17}H_{21}N_6O_3$ requires 357.1670.

2-Acetamido-N-benzyl--6-(piperidin-1-yl)-5-nitrosopyrimidin-4-amine **(6jb)**: blue wax; yield 69%. IR (KBr): ν_{max} = 3206 (NH), 1689 (C=O) cm^{-1}. ^1H NMR (400 MHz, $CDCl_3$, 25 °C): δ = 1.74 (6H, br. s, $(CH_2)_3$), 2.50 (3H, s, $COCH_3$), 4.09 (4H, br. s, $N(CH_2)_2$), 4.63 (2H, d, *J* = 5.6

Hz, CH_2), 7.23 – 7.30 (5H, m, ArH), 8.22 (1H, br. s, NH), 12.37 (1H, br. s, NH) ppm. ^{13}C NMR (100 Hz, $CDCl_3$, 25 °C): δ = 24.59, 25.84, 26.86 (CO<u>CH</u>$_3$, $(CH_2)_3$), 44.14 (N<u>CH</u>$_2$Bn), 49.08 and 52.80 (broad, N<u>CH</u>$_2$), 127.49, 127.60 128.73, 137.00 (Ar-C), 140.98 (C-5), 150.53, 158.00, 162.34 (C-4, C-6 and NH<u>CO</u>CH_3), 172.35 (C-2) ppm. HRMS (ES): MH+, found 355.1877. $C_{18}H_{23}N_6O_2$ requires 355.1877.

2-Acetamido-N-butyl-6-morpholino-5-nitrosopyrimidin-4-amine **(6ka)**: blue solid; yield 79%; mp 82–84 ^oC. IR (KBr): ν_{max} = 3190 (NH), 1674 (C=O) cm^{-1}. ^1H NMR (400 MHz, $CDCl_3$, 25 °C): δ = 0.92 (3H, t, *J* = 7.2 Hz, CH_3), 1.38 (2H, sext., *J* = 7.2 Hz, CH_2), 1.58 (2H, pent., *J* = 7.2 Hz, CH_2), 1.73 – 1.81 (2H, m, CH_2), 2.55 (3H, s, $COCH_3$), 3.40 – 3.45 (2H, m, NH<u>CH</u>$_2$), 3.76 – 3.82 (4H, m, $N(CH_2)_2$), 4.22 (4H, br. s, $O(CH_2)_2$), 8.13 (1H, br. s, NH), 12.08 (1H, br. s, NH) ppm. ^{13}C NMR (100 Hz, $CDCl_3$, 25 °C): δ = 13.72 (CH_3), 20.22 (CH_2), 25.85 (CO<u>CH</u>$_3$), 30.92, 40.19 (N<u>CH</u>$_2$Bn), 49.54 (broad, N<u>CH</u>$_2$), 67.25 (O<u>CH</u>$_2$), 141.18 (C-5), 150.45, 157.98, 162.79 (C-4, C-6 and NH<u>CO</u>CH_3), 172.09 (C-2) ppm. HRMS (ES): MH+, found 323.1826. $C_{14}H_{23}N_6O_3$ requires 323.1824.

N-Benzyl-2,6-dimorpholino-5-nitrosopyrimidin-4-amine **(6la)**: blue solid; yield 72%; mp 162–164 ^oC. IR (KBr): ν_{max} = 3153, 3088 (NH) cm^{-1}. ^1H NMR (400 MHz, $CDCl_3$, 25 °C): δ = 3.69 – 3.91 (12H, m, $O(CH_2)_2$, $N(CH_2)_4$), 4.21 (4H, s, $O(CH_2)_2$), 4.67 (2H, d, *J* = 5.6 Hz, $PhCH_2$), 7.28 – 7.36 (5H, m, ArH), 12.14 (1H, br s, NH) ppm. ^{13}C NMR (100 MHz, $CDCl_3$, 25 °C): δ = 44.0, 44.7, 49.3 (NCH_2), 66.7, 67.2 (OCH_2), 127.4, 127.6, 128.6, 137.7 (Ar-C), 139.8 (C-5), 159.5, 151.7 (C-4 and C-6), 162.3 (C-2) ppm. HRMS (ES): MH^+, found 385.1980. $C_{19}H_{25}N_6O_3$ requires 385.1983.

N-Benzyl-4-chloro-6-morpholino-N-nitrosopyrimidin-2-amine **(7):** white solid; yield 91%; mp 123–125 ^oC. ^1H NMR (400 MHz, $CDCl_3$, 25 °C): δ = 3.67 (4H, br. s, $N(CH_2)_2$), 3.76 – 3.79 (4H, m, $O(CH_2)_2$), 5.31 (2H, s, NCH_2Ph), 6.45 (1H, s, C(5)-H), 7.20 – 7.34 (5H, m, ArH) ppm. ^{13}C NMR (100 MHz, $CDCl_3$, 25 °C): δ = 45.30, 44.58, (NCH_2), 66.28 (OCH_2), 98.89 (C-5), 127.45, 127.85, 128.47, 135.24 (Ar-C), 158.27, 160.97 (C-4 and C-6), 163.07 (C-2) ppm. HRMS (ES): MH^+, found 334.1071. $C_{15}H_{17}ClN_5O_2$ requires 334.1071.

2-Benzyl-4,6-dimorpholino-5-nitrosopyrimidin-2-amine **(8)**: violet solid; yield 92%; mp 80–82 ^oC. IR (KBr): ν_{max} = 3277 (NH) cm^{-1}. NMR spectra contain signals of two tautomers. ^1H NMR (400 MHz, $CDCl_3$, 25 °C): δ = 3.11 (2H, br. s, $N(CH_2)_2$), 3.69 – 3.83 (10H, m, $O(CH_2)_2$, $N(CH_2)_4$), 4.00 – 4.09 (4H, m, $O(CH_2)_2$), 4.54 – 4.61 (2H, m, $PhCH_2$), 6.18 (1H, br s, NH), 7.26 – 7.28 (5H, m, ArH) ppm. ^{13}C NMR (100 MHz, $CDCl_3$, 25 °C): δ = 45.14, 45.53, 51.64, 51.62 (NCH_2), 66.36, 67.11 (OCH_2), 125.08, 127.31, 128.01, 128.43, 128.81, 138.14 (Ar-C), 140.42 (C-5), 150.74, 150.89, 160.48, 160.48 (C-4 and C-6), 165.26, 165.50 (C-2) ppm. HRMS (ES): MH^+, found 385.1988. $C_{19}H_{25}N_6O_3$ requires 385.1983.

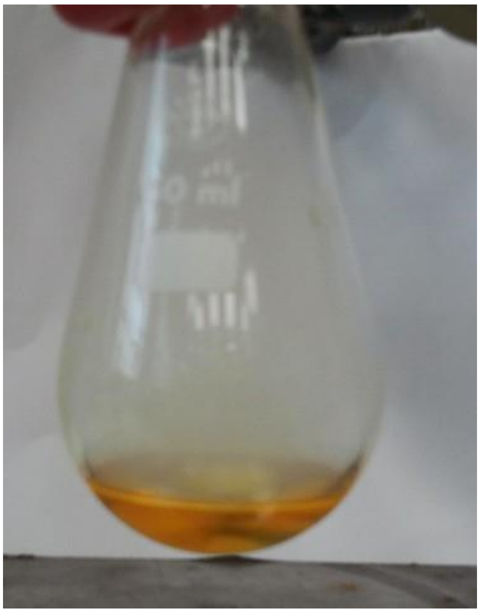

Figure S1a: Typical one-pot nucleophilic substitution – rearrangement procedure. Stirring in 50% H_2SO_4 step.

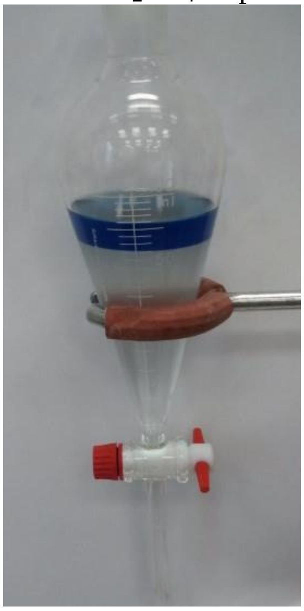

Figure S1b: Typical one-pot nucleophilic substitution – rearrangement procedure. Extraction of final product after the quenching of reaction mixture (Figure. S1a) with sodium bicarbonate.

References

1. van der Westhuyzen, C. W.; Rousseau, A. L.; Parkinson C. J. *Tetrahedron* **2007**, *63*, 5394.
2. Wan, Z.-K.; Wacharasindhu, S.; Levins, C. G.; Lin, M.; Tabei, K.; Mansour, T. S. *J. Org. Chem*. **2007**, *72*, 10194.
3. Jakubkiene, V.; Cikotiene, I. *Tetrahedron* **2012**, *68*, 2294.
4. Jakubkiene, V.; Zurba, J.; Cikotiene, I. *Synthesi*, **2013**, *45*, 448.

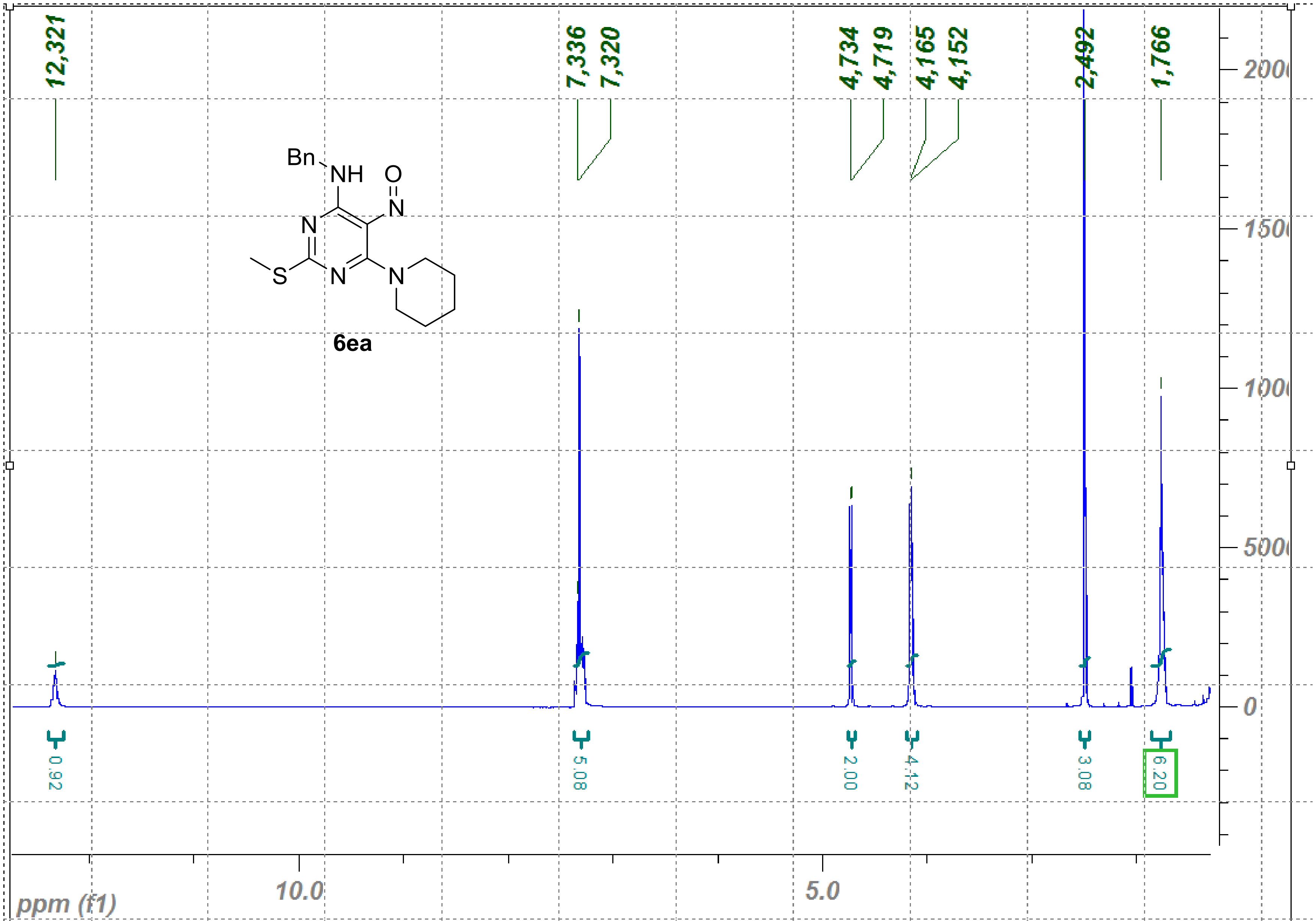
12,321
7,336
7,320
4,734
4,719
4,165
4,152
2,492
1,766
Bn
NH
O
N
N
N
N
S
6ea
0.92
5.08
2.00
4.12
3.08
6.20
10.0
5.0
ppm (f1)
0
500
1000
1500
2000

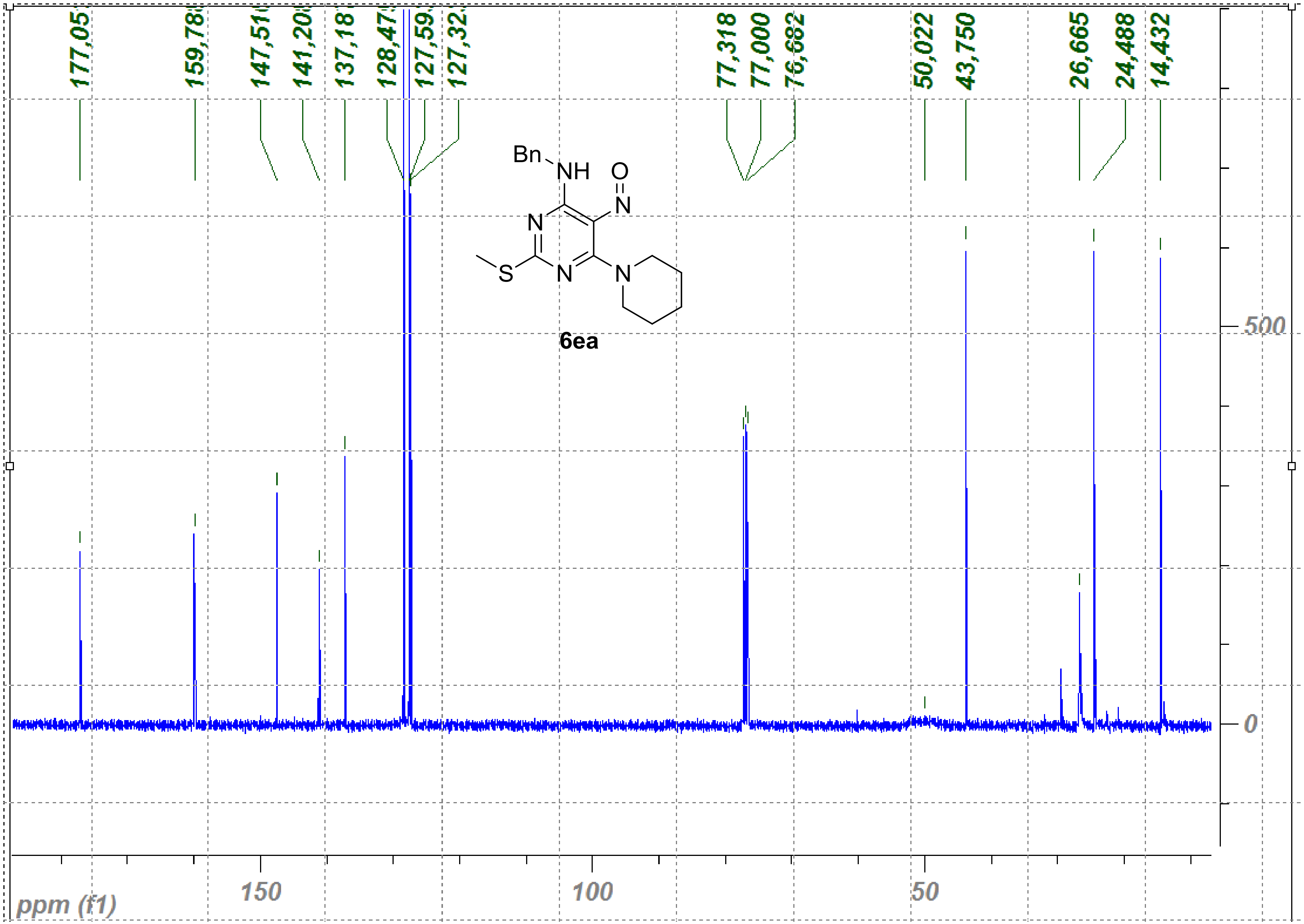
177,05
159,78
147,51
141,20
137,18
128,47
127,59
127,32
77,318
77,000
76,682
50,022
43,750
26,665
24,488
14,432
Bn
NH
O
N
N
S
N
N
6ea
500
0
150
100
50
ppm (f1)

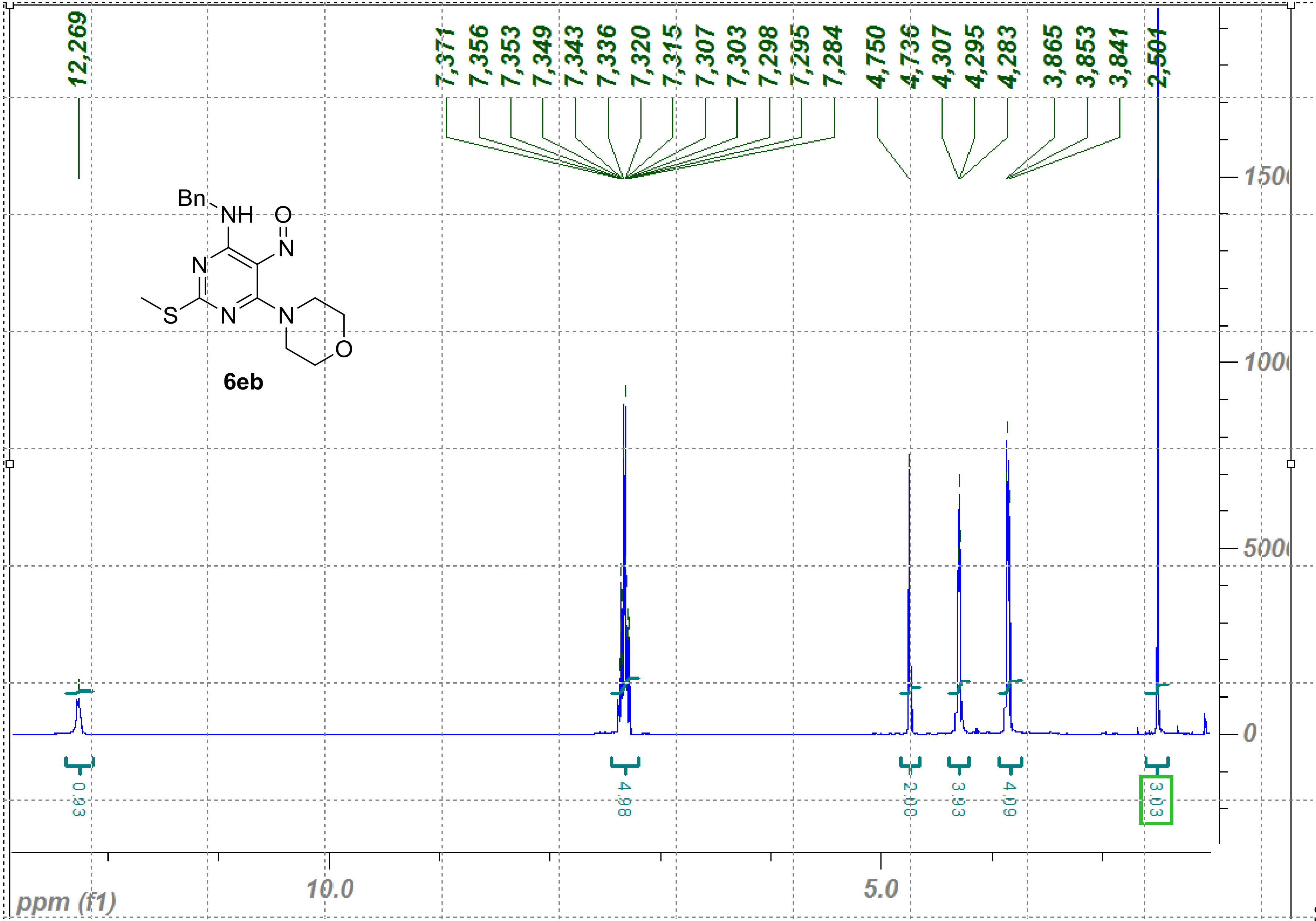
12,269
7,371
7,356
7,353
7,349
7,343
7,336
7,320
7,315
7,307
7,303
7,298
7,295
7,284
4,750
4,736
4,307
4,295
4,283
3,865
3,853
3,841
2,501
Bn
NH
O
N
N
S
N
N
O
6eb
0.93
4.98
2.00
3.93
4.09
3.03
ppm (f1)
10.0
5.0
1500
1000
500
0

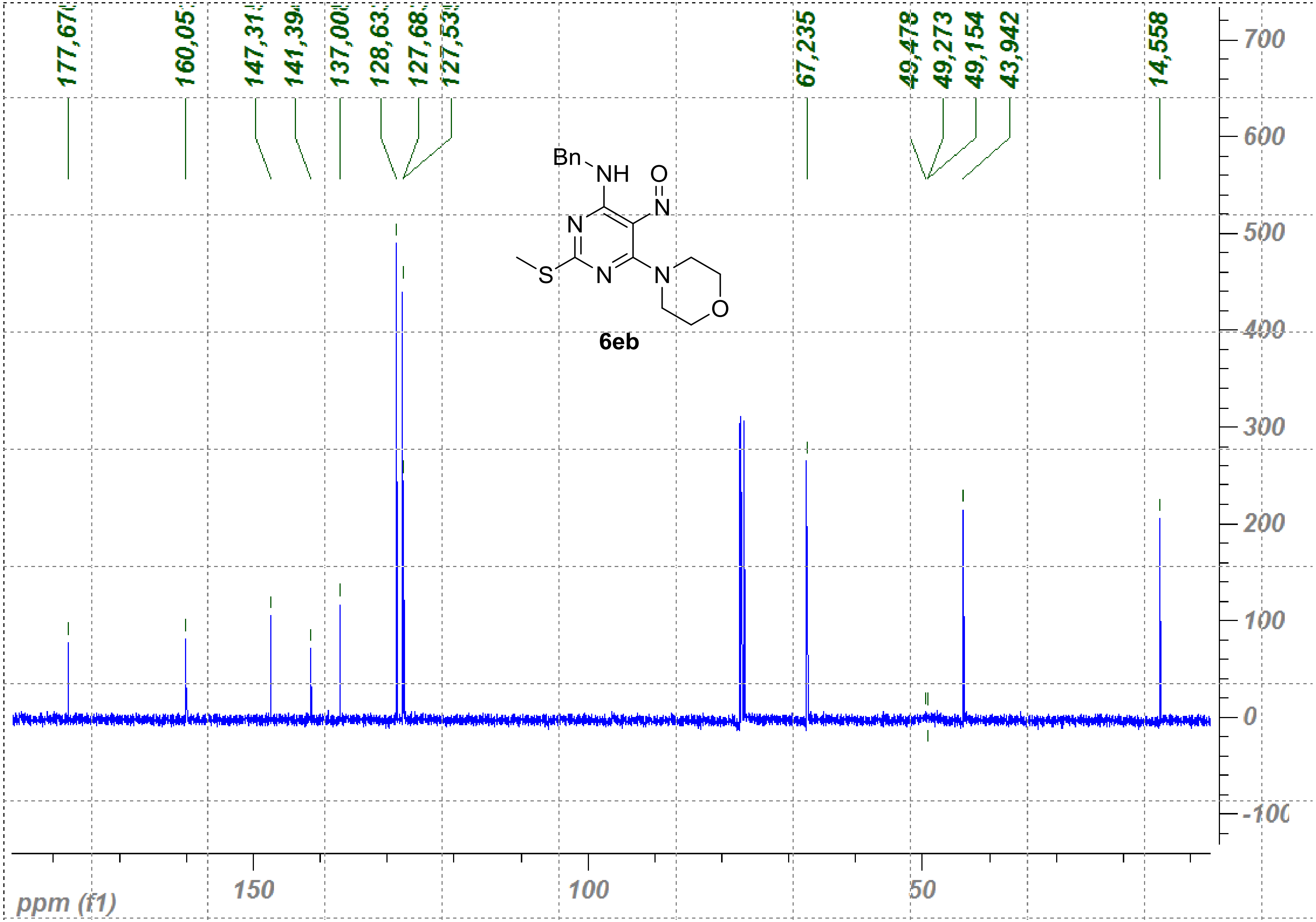

177,67
160,05
147,31
141,39
137,00
128,63
127,68
127,53
67,235
49,478
49,273
49,154
43,942
14,558
Bn
NH
O
N
N
S
N
N
O
6eb
700
600
500
400
300
200
100
0
-100
ppm (f1)
150
100
50

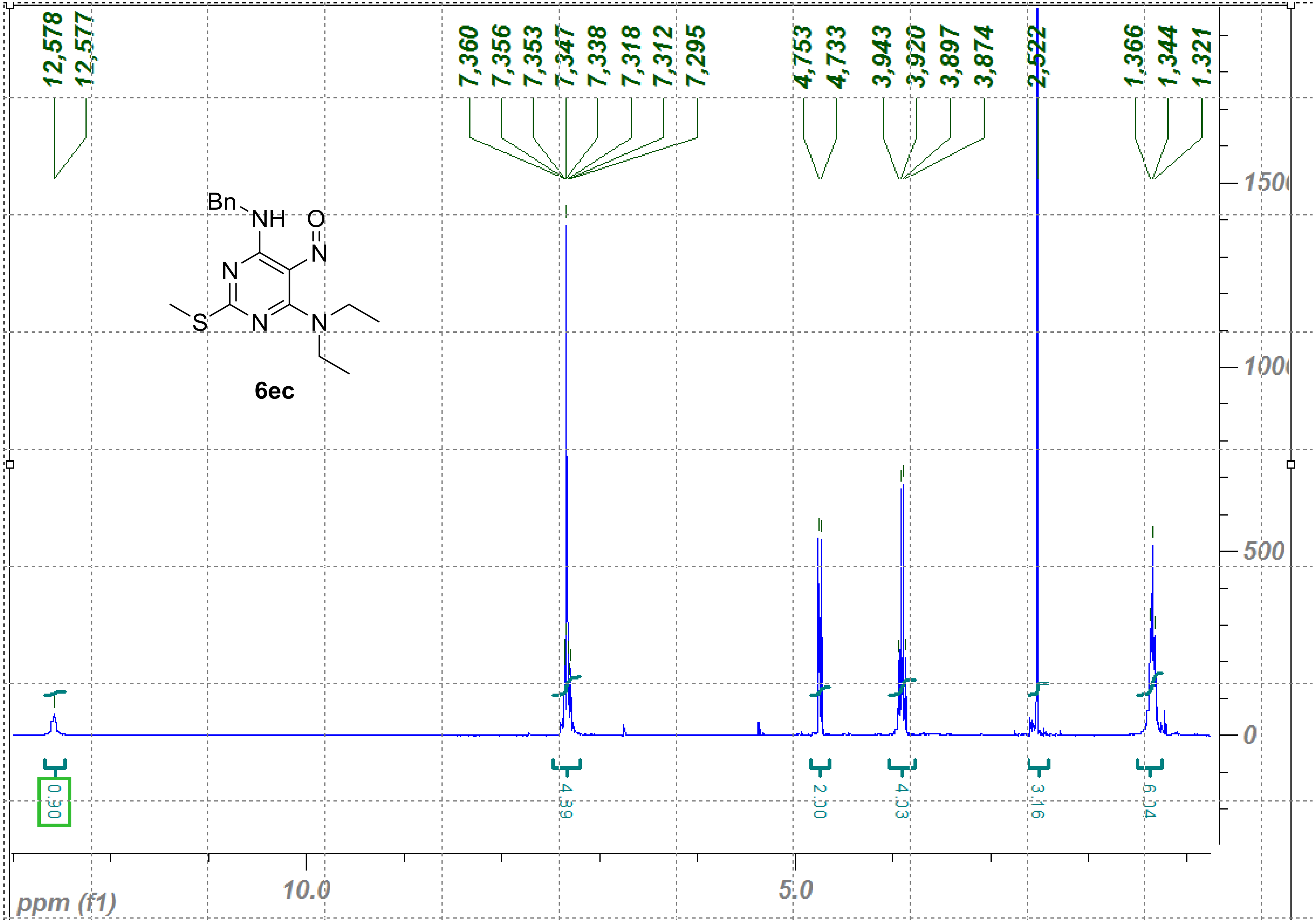
12,578
12,577
7,360
7,356
7,353
7,347
7,338
7,318
7,312
7,295
4,753
4,733
3,943
3,920
3,897
3,874
2,522
1,366
1,344
1,321
Bn
NH
O
N
N
N
S
N
6ec
0.90
4.39
2.00
4.03
3.16
6.04
1500
1000
500
0
10.0
5.0
ppm (f1)

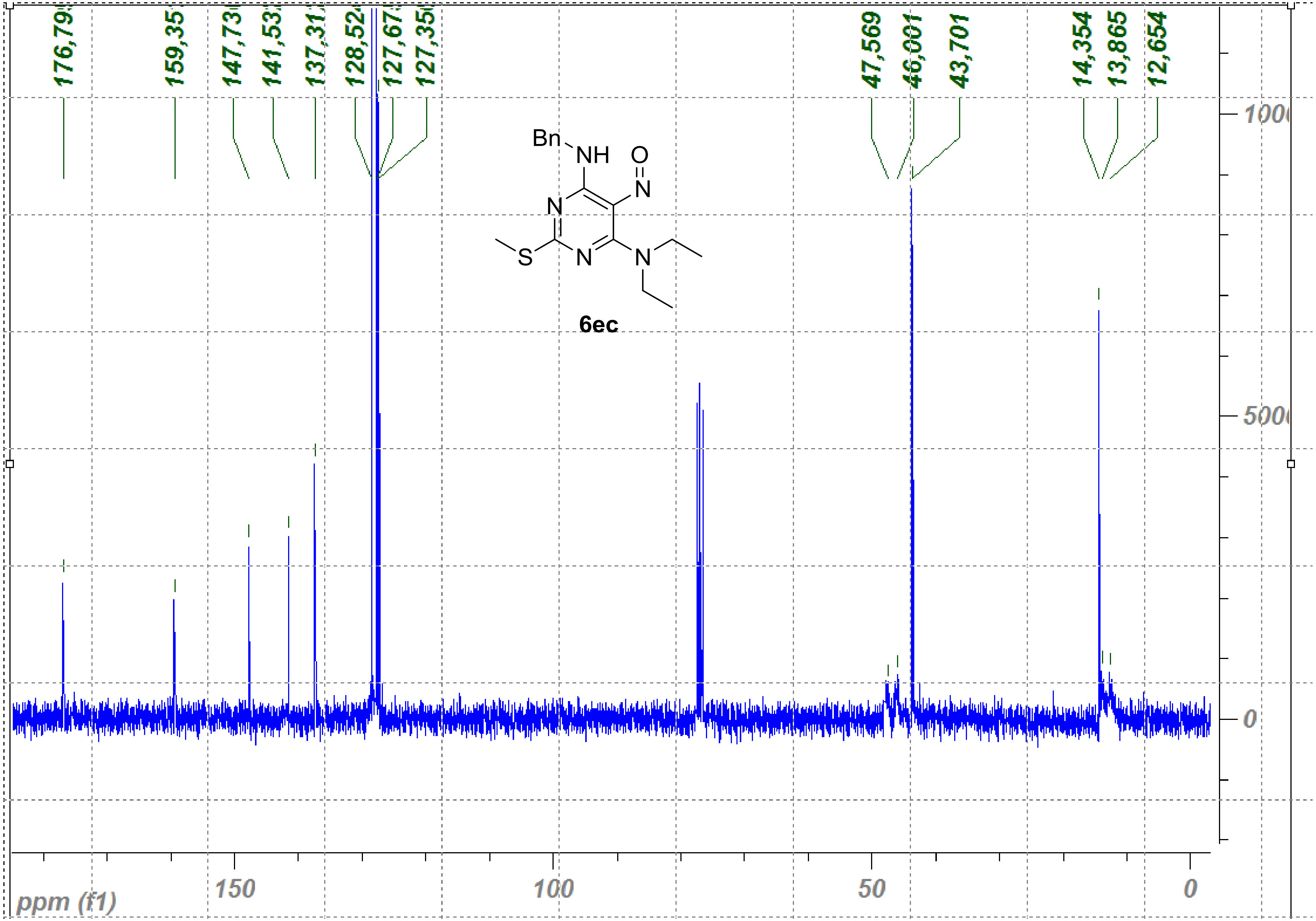

176,79
159,35
147,73
141,53
137,31
128,52
127,67
127,35
47,569
46,001
43,701
14,354
13,865
12,654
Bn
NH
O
N
N
N
S
N
6ec
1000
5000
0
150
100
50
0
ppm (f1)

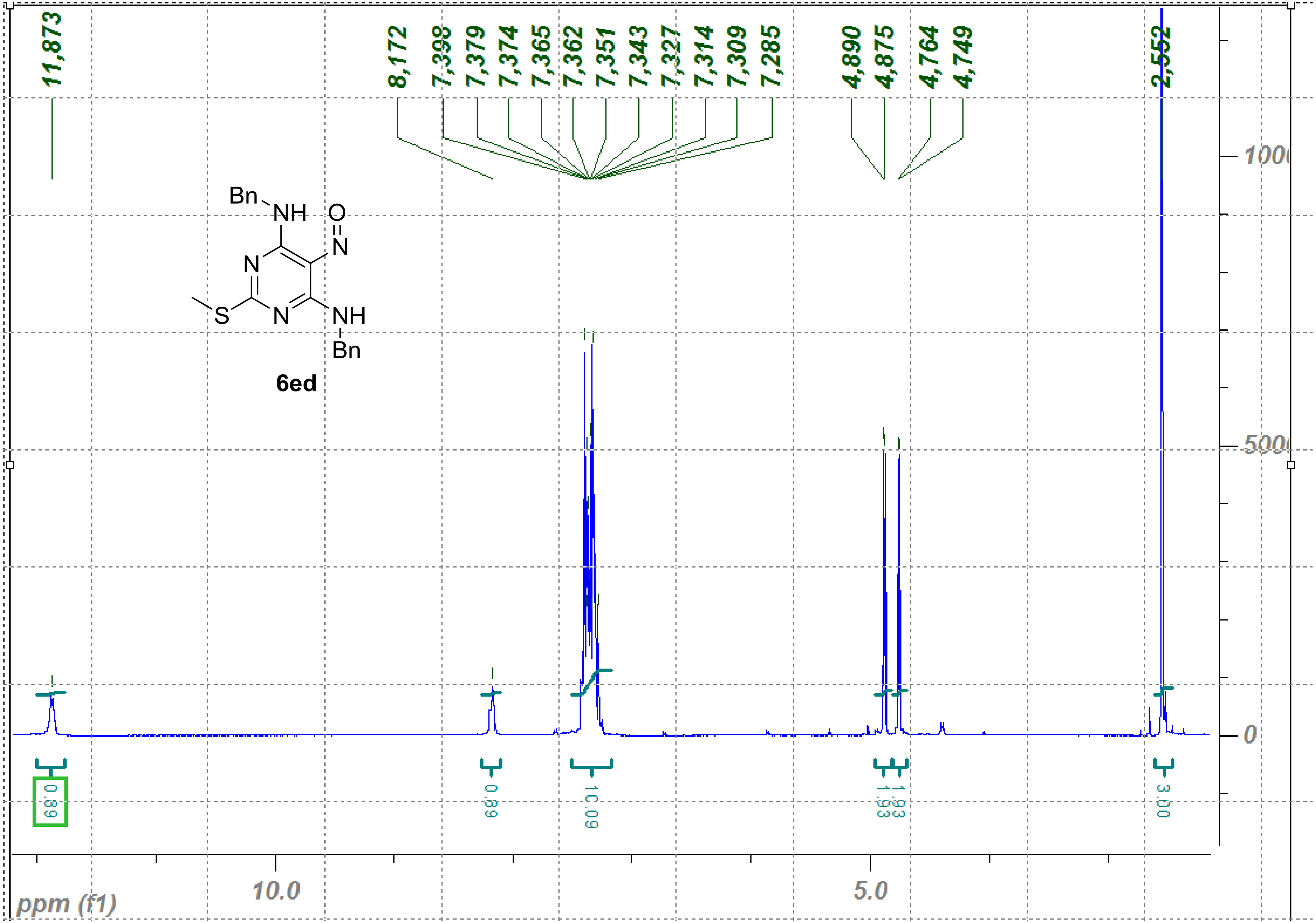
11,873
8,172
7,398
7,379
7,374
7,365
7,362
7,351
7,343
7,327
7,314
7,309
7,285
4,890
4,875
4,764
4,749
2,552
Bn
NH
O
N
N
N
S
NH
Bn
6ed
0.89
0.89
10.09
1.93
1.93
3.00
10.0
5.0
ppm (f1)
1000
500
0

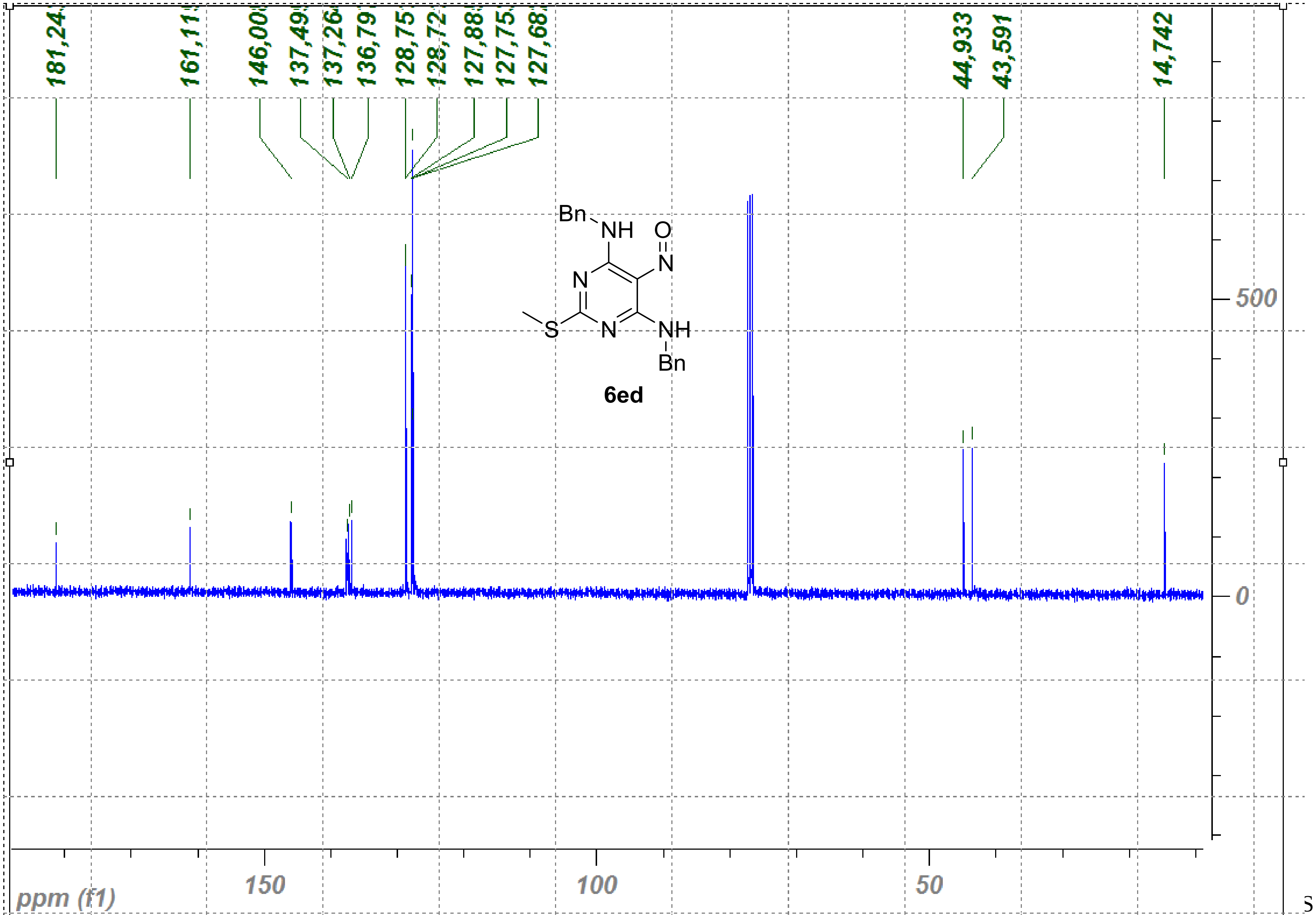

181,24
161,11
146,00
137,49
137,26
136,79
128,75
128,72
127,88
127,75
127,68
44,933
43,591
14,742
Bn
NH
O
N
N
S
N
NH
Bn
6ed
500
0
ppm (f1)
150
100
50

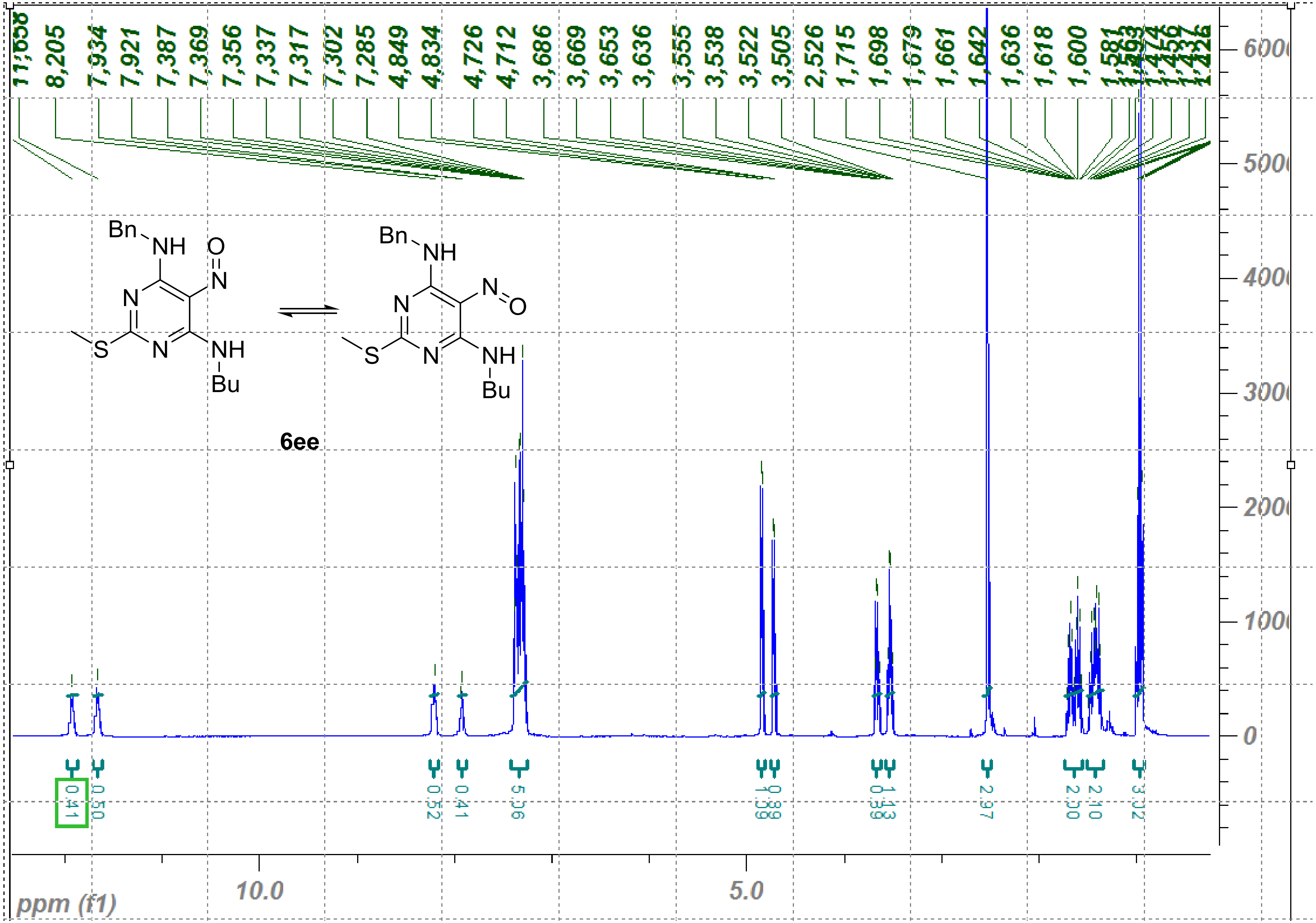
8,205
7,934
7,921
7,387
7,369
7,356
7,337
7,317
7,302
7,285
4,849
4,834
4,726
4,712
3,686
3,669
3,653
3,636
3,555
3,538
3,522
3,505
2,526
1,715
1,698
1,679
1,661
1,642
1,636
1,618
1,600
Bn
NH
O
N
N
S
N
NH
Bu
6ee
0.41
0.50
0.52
0.41
5.06
1.13
2.97
2.10
3.02
10.0
5.0
ppm (f1)

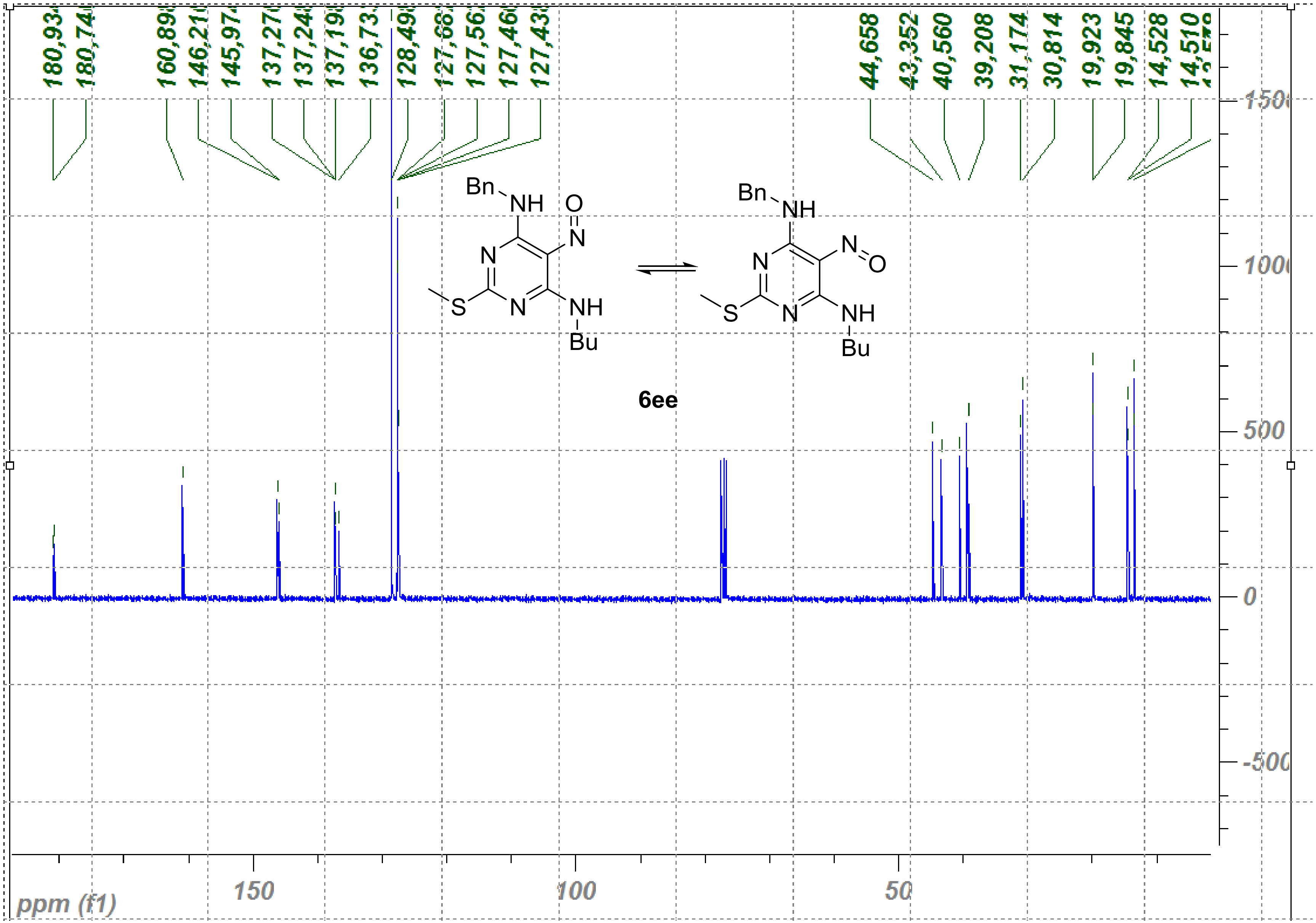
180,93
180,74
160,89
146,21
145,97
137,27
137,24
137,19
136,73
128,49
127,68
127,56
127,46
127,43
44,658
43,352
40,560
39,208
31,174
30,814
19,923
19,845
14,528
14,510
Bn
NH
O
N
N
N
S
NH
Bu
6ee
ppm (f1)
150
100
50
1500
1000
500
0
-500

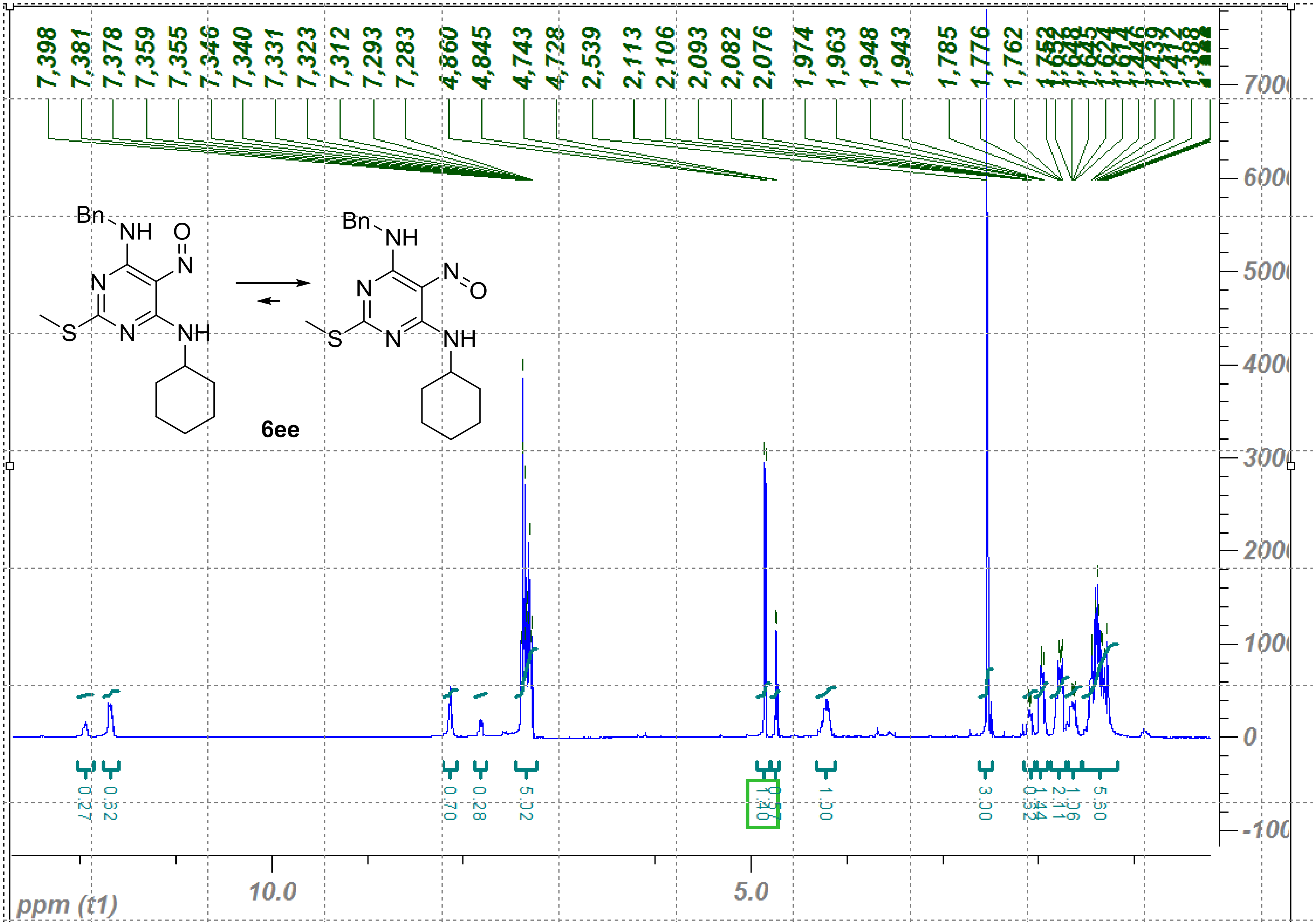

6ee
ppm (t1)
10.0
5.0

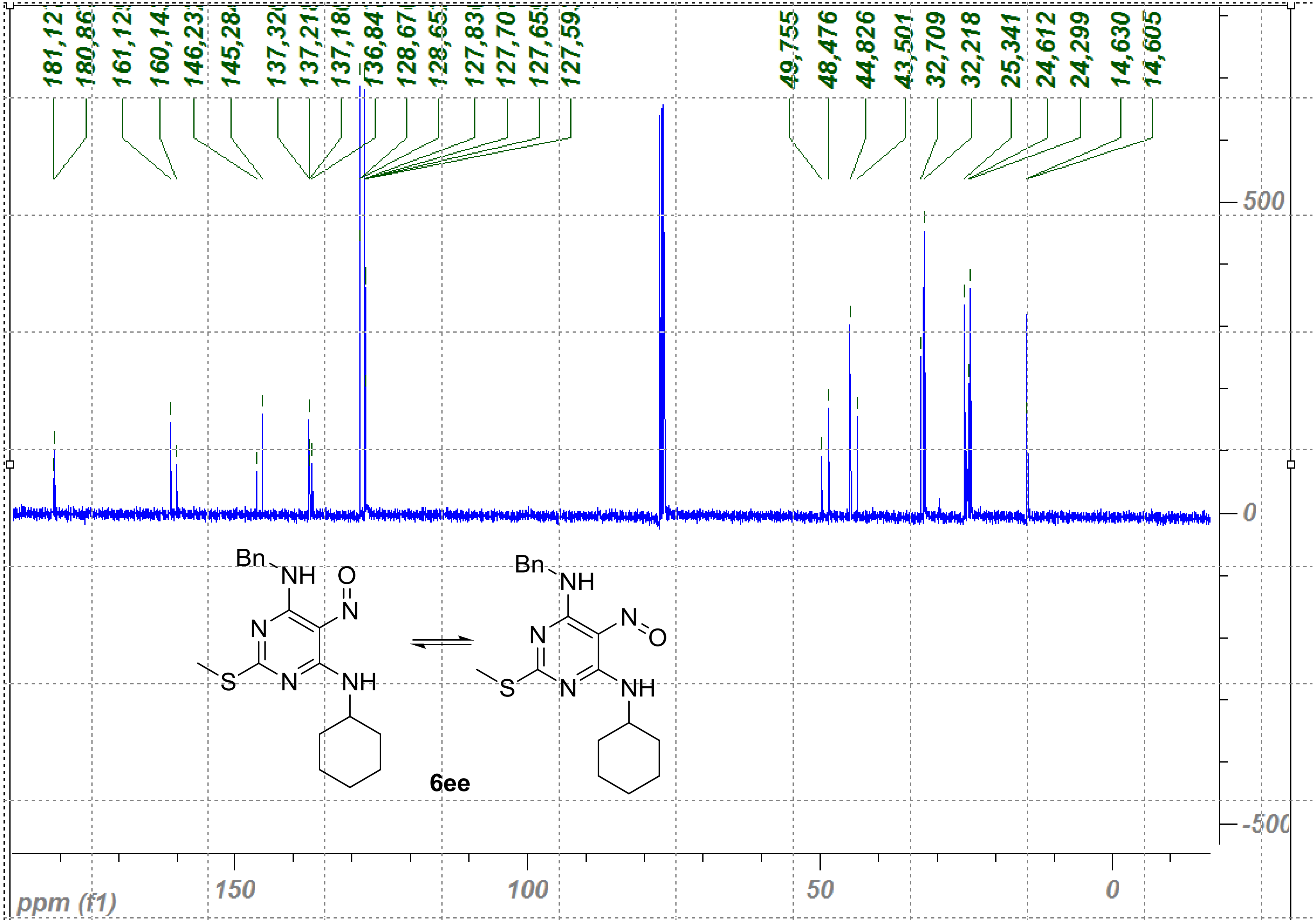
181,12
180,86
161,12
160,14
146,23
145,28
137,32
137,21
137,18
136,84
128,67
128,65
127,83
127,70
127,65
127,59
49,755
48,476
44,826
43,501
32,709
32,218
25,341
24,612
24,299
14,630
14,605
500
0
-500
150
100
50
0
ppm (f1)
Bn
NH
O
N
N
S
N
NH
N
O
6ee

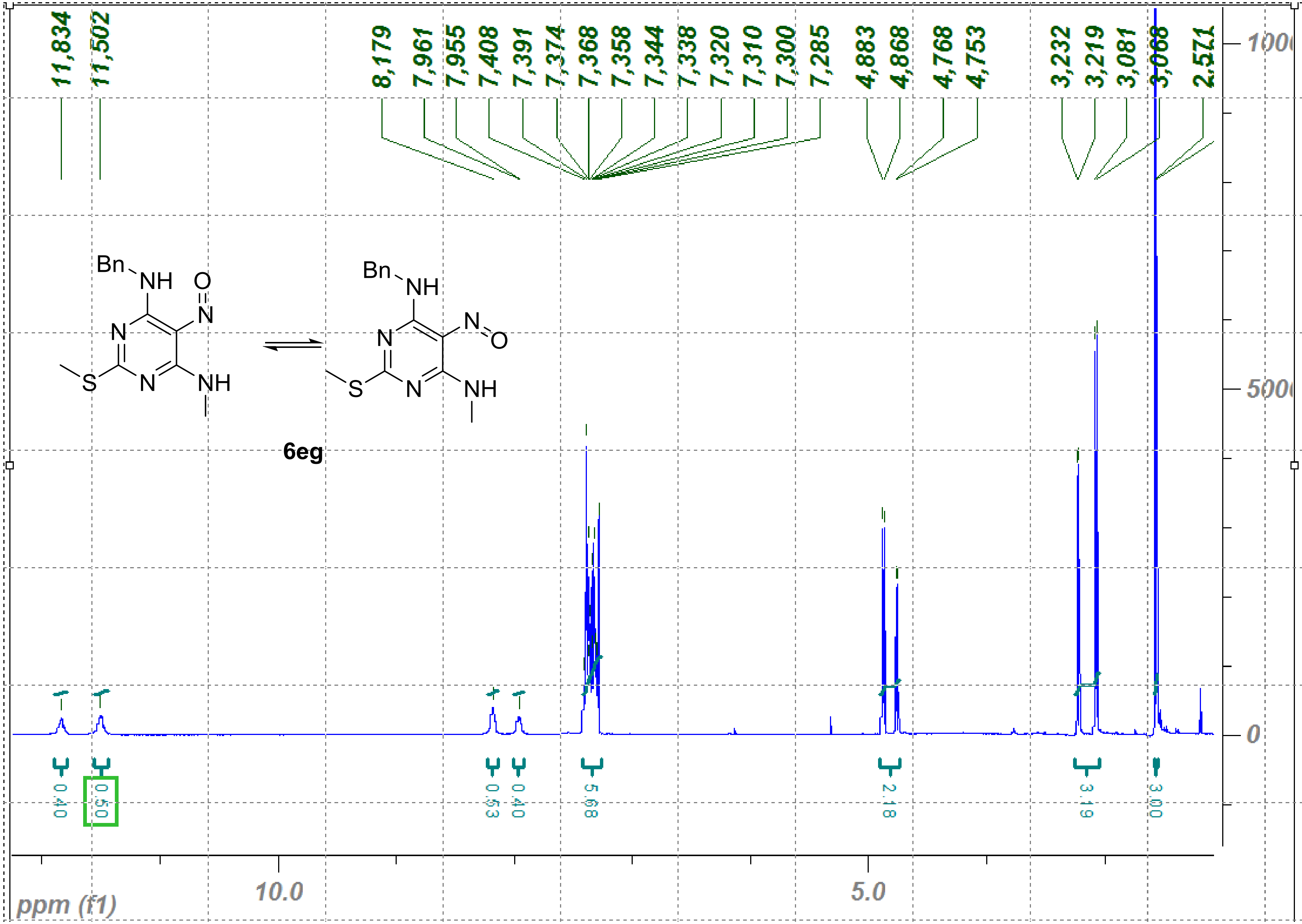
11,834
11,502
8,179
7,961
7,955
7,408
7,391
7,374
7,368
7,358
7,344
7,338
7,320
7,310
7,300
7,285
4,883
4,868
4,768
4,753
3,232
3,219
3,081
3,068
2,571
Bn
NH
O
N
N
N
S
NH
6eg
0.40
0.50
0.53
0.40
5.68
2.18
3.19
3.00
10.0
5.0
ppm (f1)
0

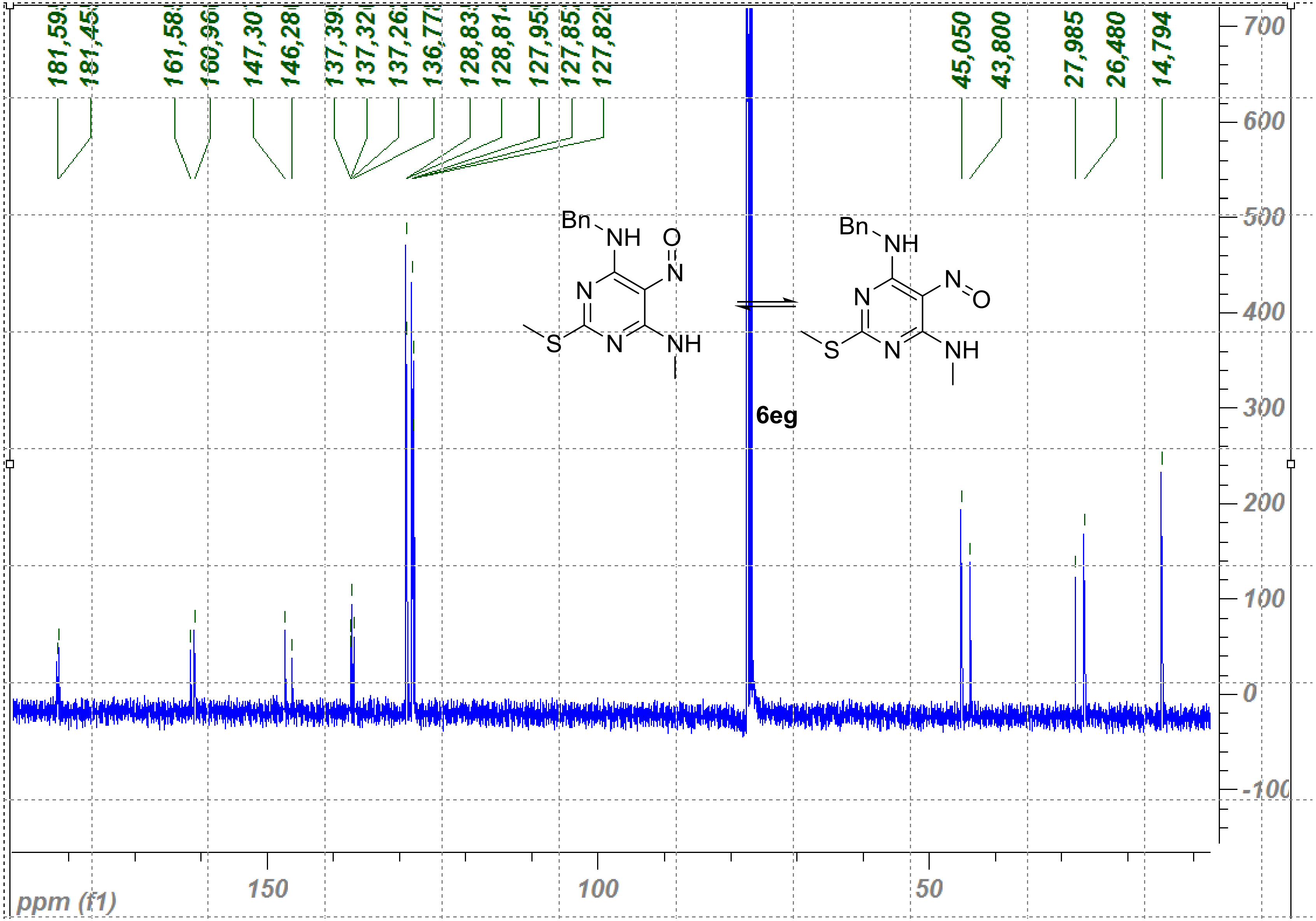

181,59
181,45
161,58
160,96
147,30
146,28
137,39
137,32
137,26
136,77
128,83
128,81
127,95
127,85
127,82
45,050
43,800
27,985
26,480
14,794
Bn
NH
O
N
N
S
N
NH
6eg
700
600
500
400
300
200
100
0
-100
150
100
50
ppm (f1)

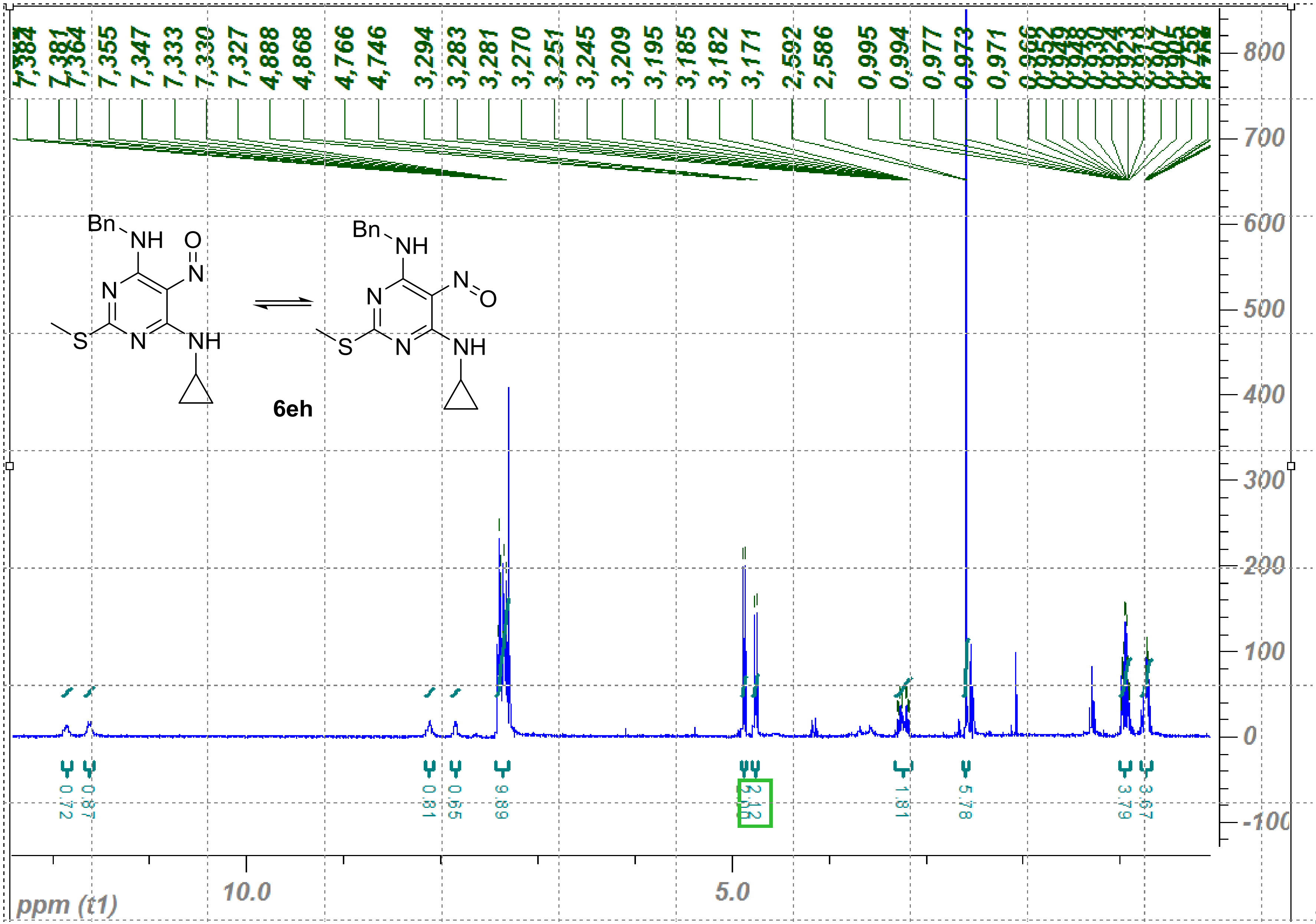
7,381
7,364
7,355
7,347
7,333
7,330
7,327
4,888
4,868
4,766
4,746
3,294
3,283
3,281
3,270
3,251
3,245
3,209
3,195
3,185
3,182
3,171
2,592
2,586
0,995
0,994
0,977
0,973
0,971
Bn
NH
O
N
N
S
N
NH
6eh
0.72
0.87
0.81
0.65
9.89
2.12
1.81
5.78
3.79
3.67
10.0
5.0
ppm (t1)
800
700
600
500
400
300
200
100
0

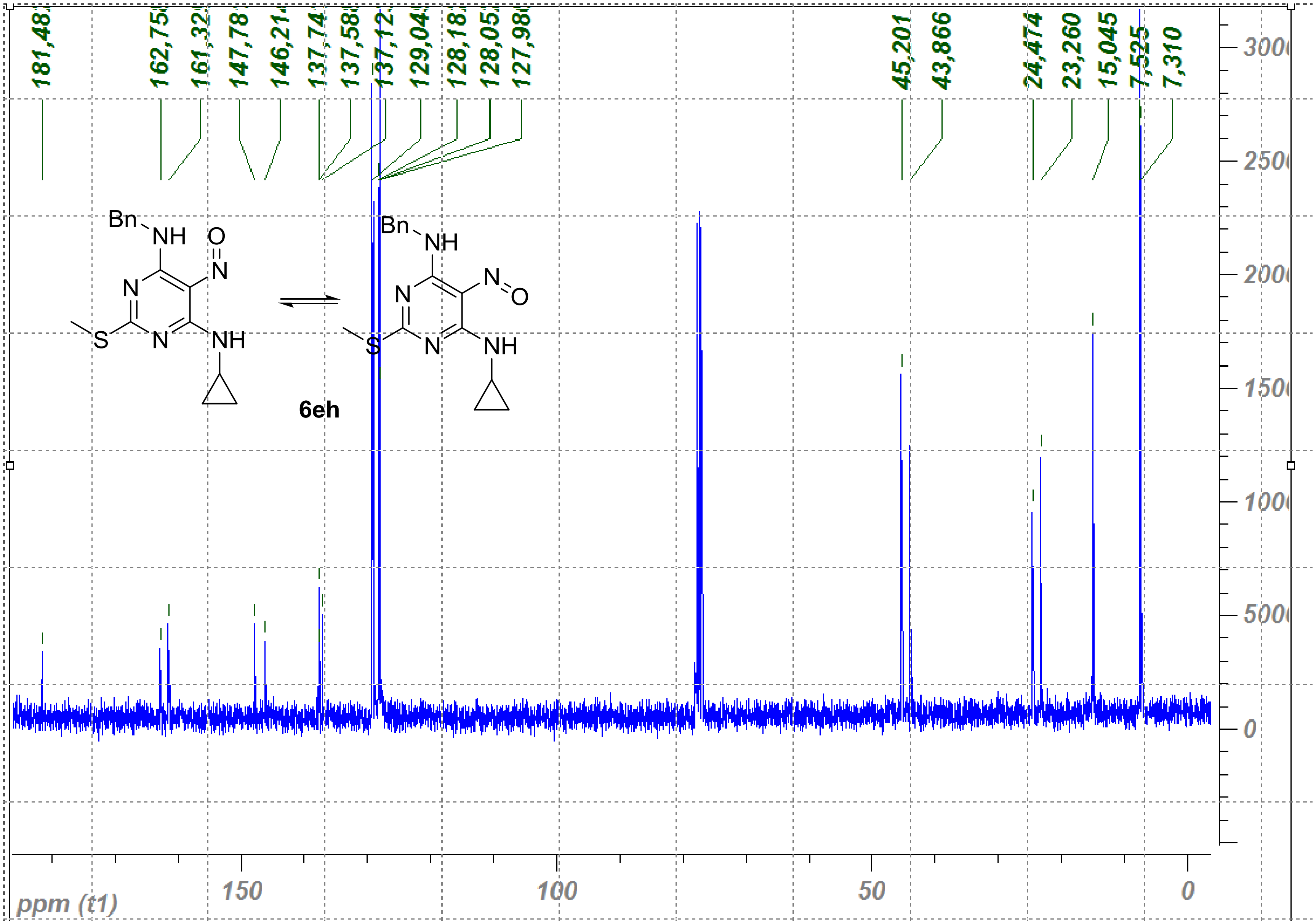
181,48
162,75
161,32
147,78
146,21
137,74
137,58
137,12
129,04
128,18
128,05
127,98
45,201
43,866
24,474
23,260
15,045
7,525
7,310
Bn
NH
O
N
N
S
N
NH
6eh
ppm (t1)
150
100
50
0

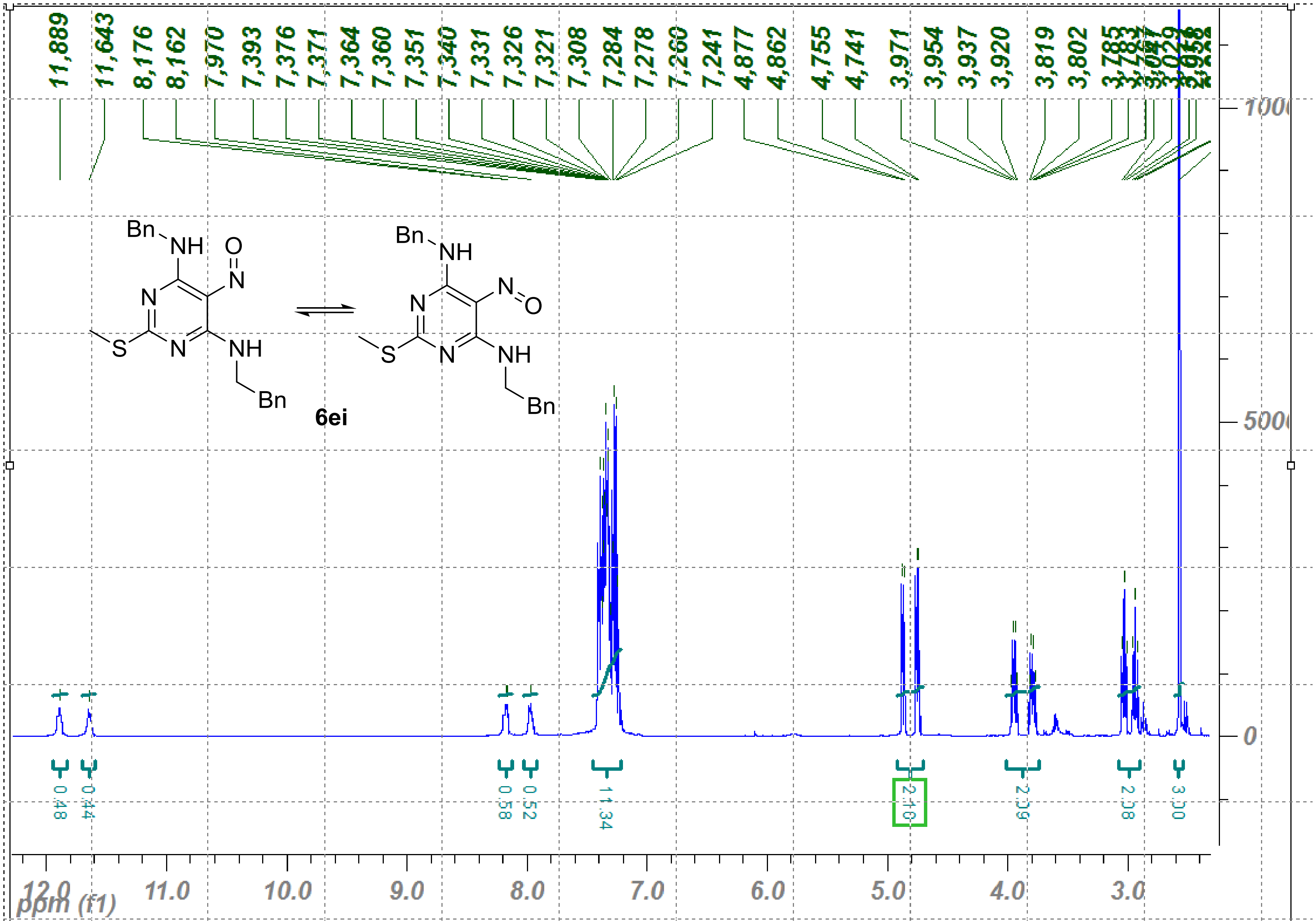

6ei
ppm (f1)

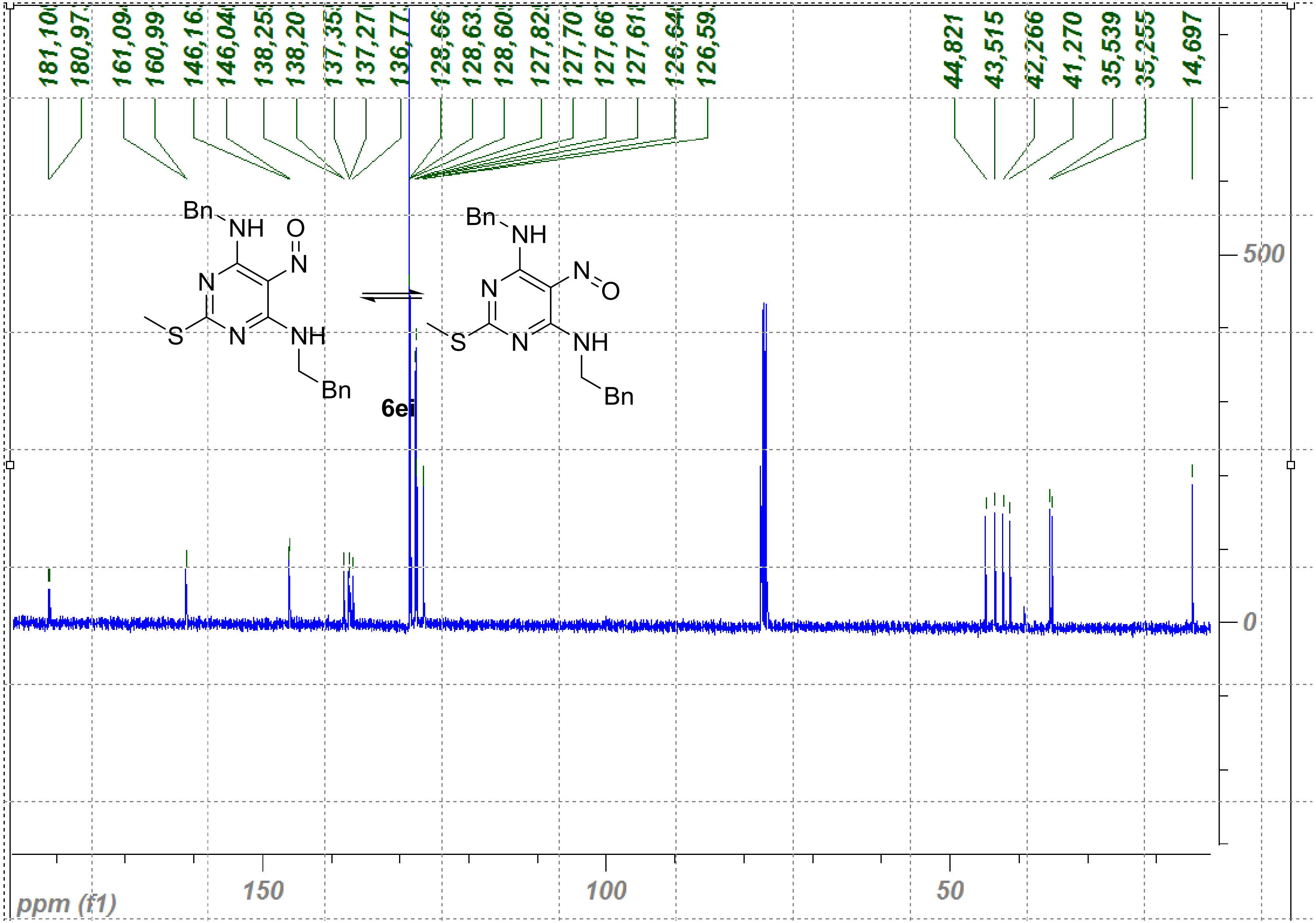
181,10
180,97
161,09
160,99
146,16
146,04
138,25
138,20
137,35
137,27
136,77
128,66
128,63
128,60
127,82
127,70
127,66
127,61
126,64
126,59
44,821
43,515
42,266
41,270
35,539
35,255
14,697
Bn
NH
O
N
N
S
N
NH
Bn
6ei
Bn
NH
N
O
N
S
N
NH
Bn
500
0
150
100
50
ppm (f1)

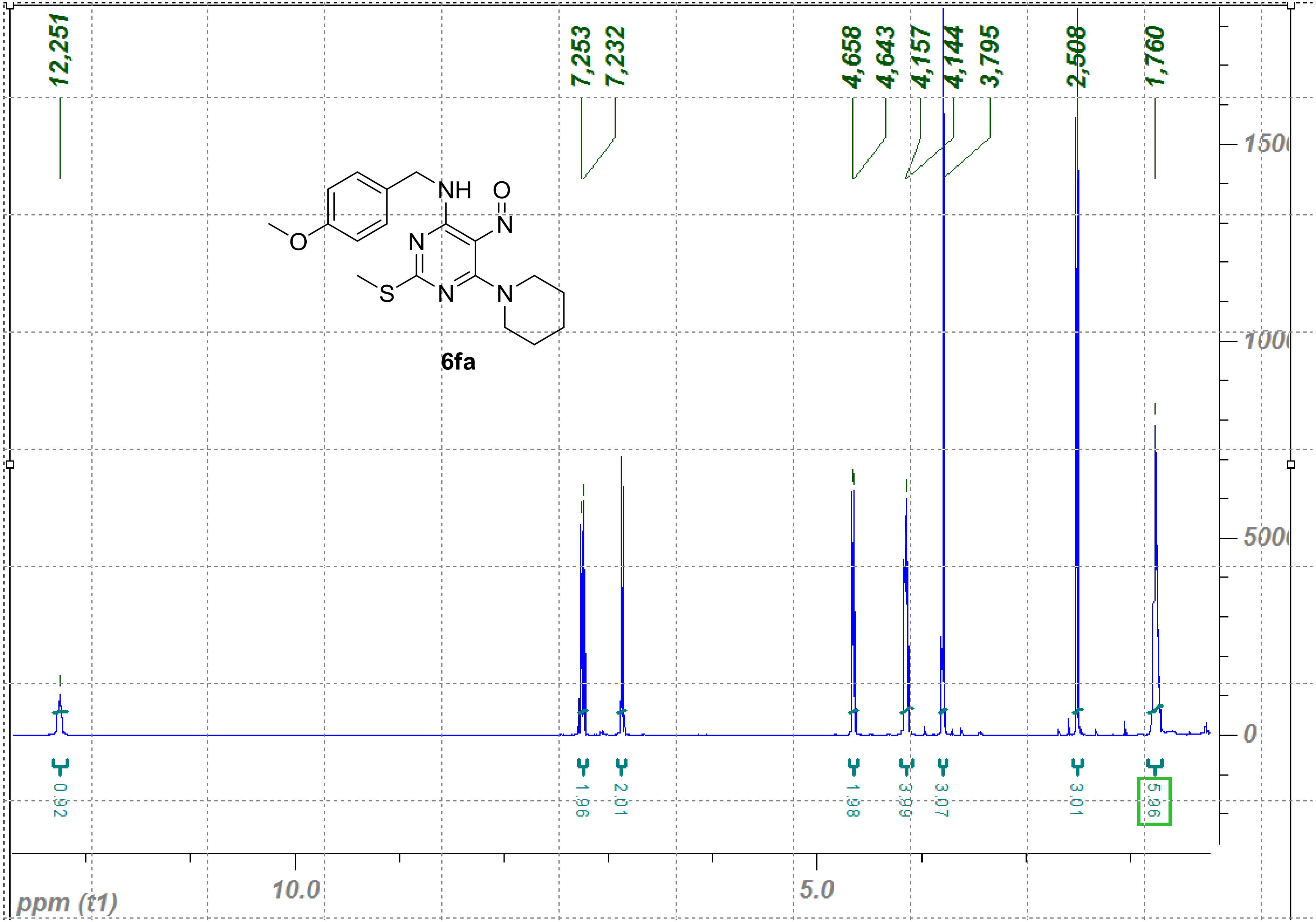

12,251
7,253
7,232
4,658
4,643
4,157
4,144
3,795
2,508
1,760
NH
O
N
N
O
S
N
N
6fa
0.92
1.96
2.01
1.98
3.99
3.07
3.01
5.96
10.0
5.0
ppm (t1)

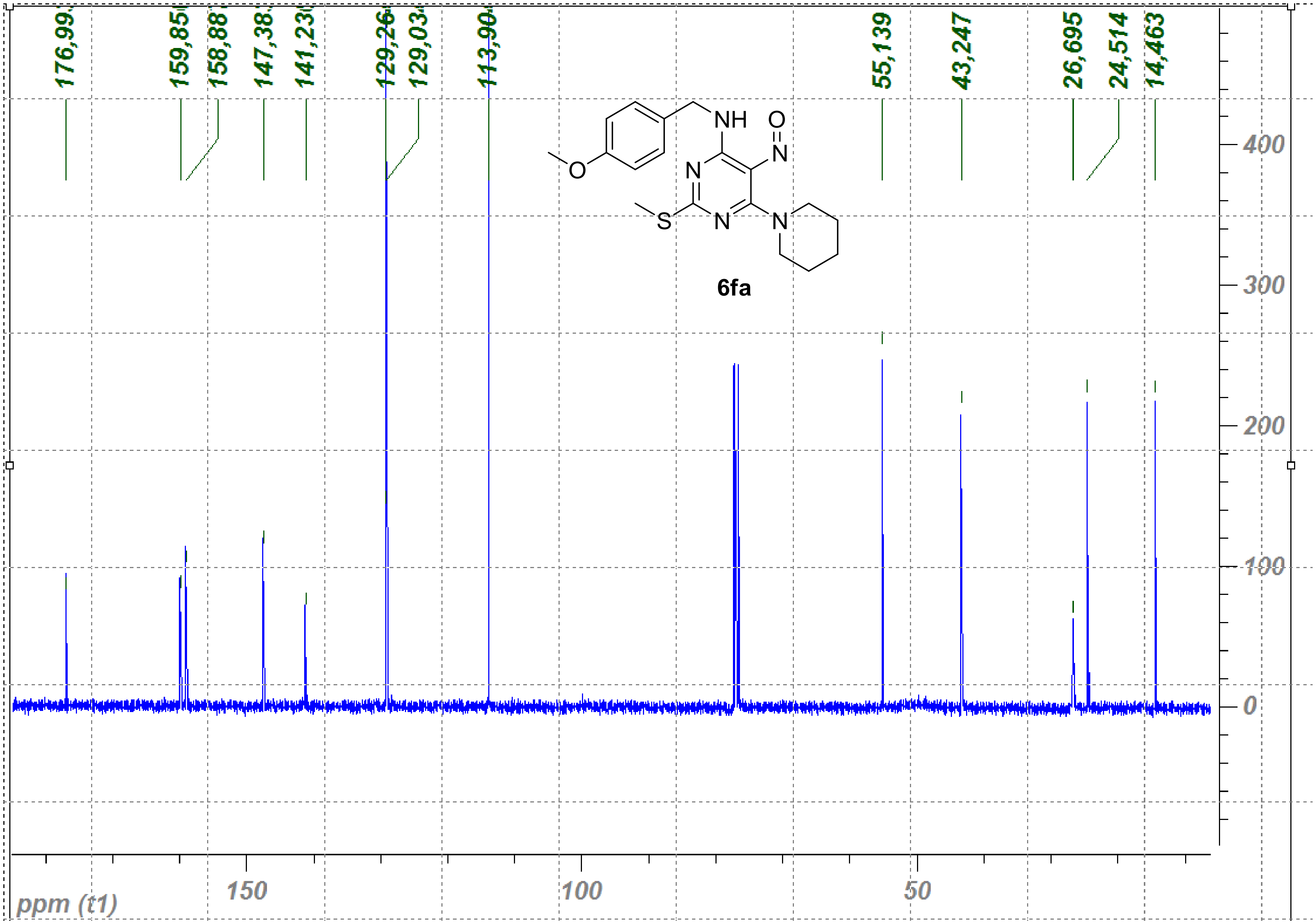

176,99
159,85
158,88
147,38
141,23
129,26
129,03
113,90
55,139
43,247
26,695
24,514
14,463
NH
O
N
N
O
N
S
N
N
6fa
400
300
200
100
0
150
100
50
ppm (t1)

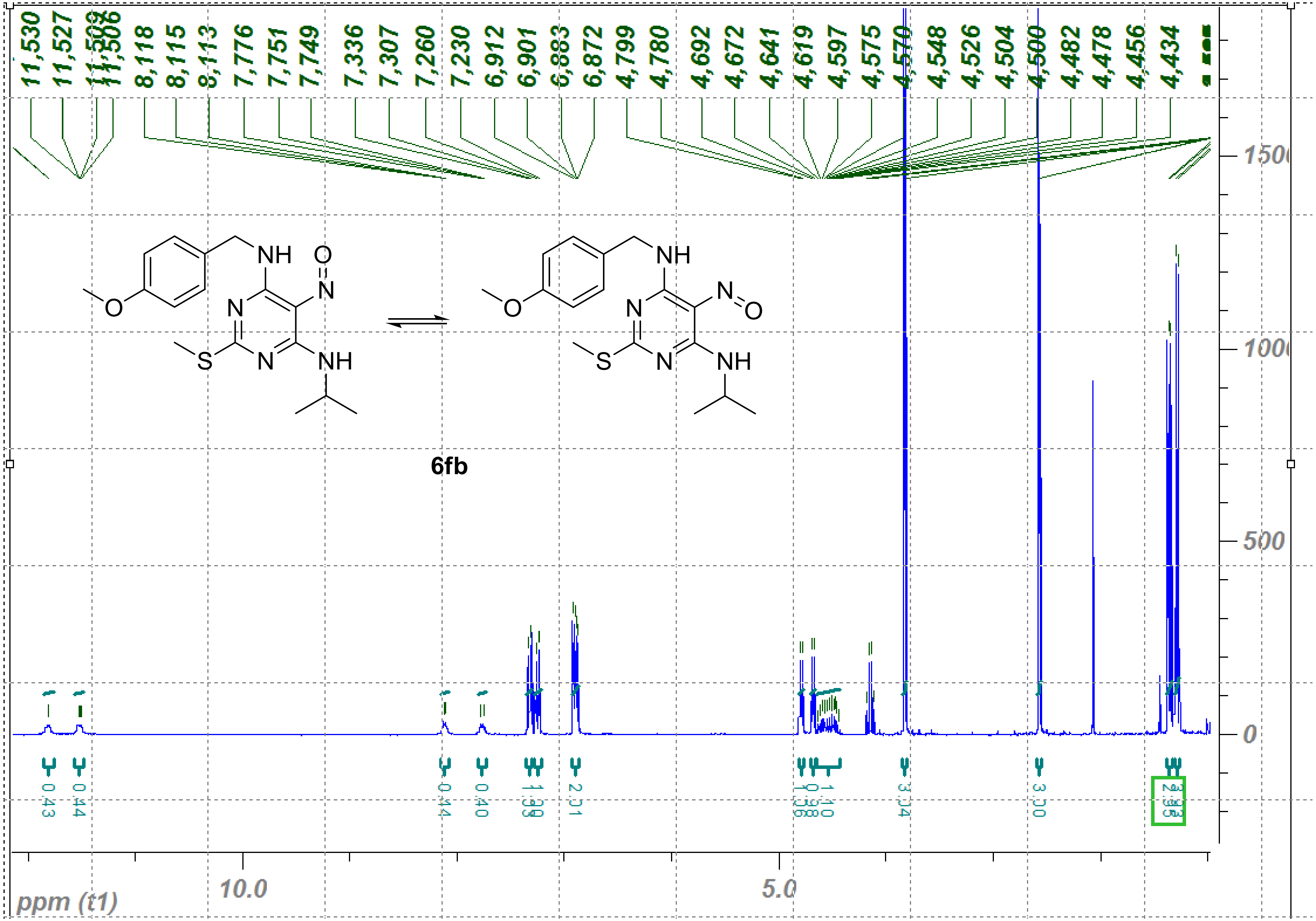

6fb

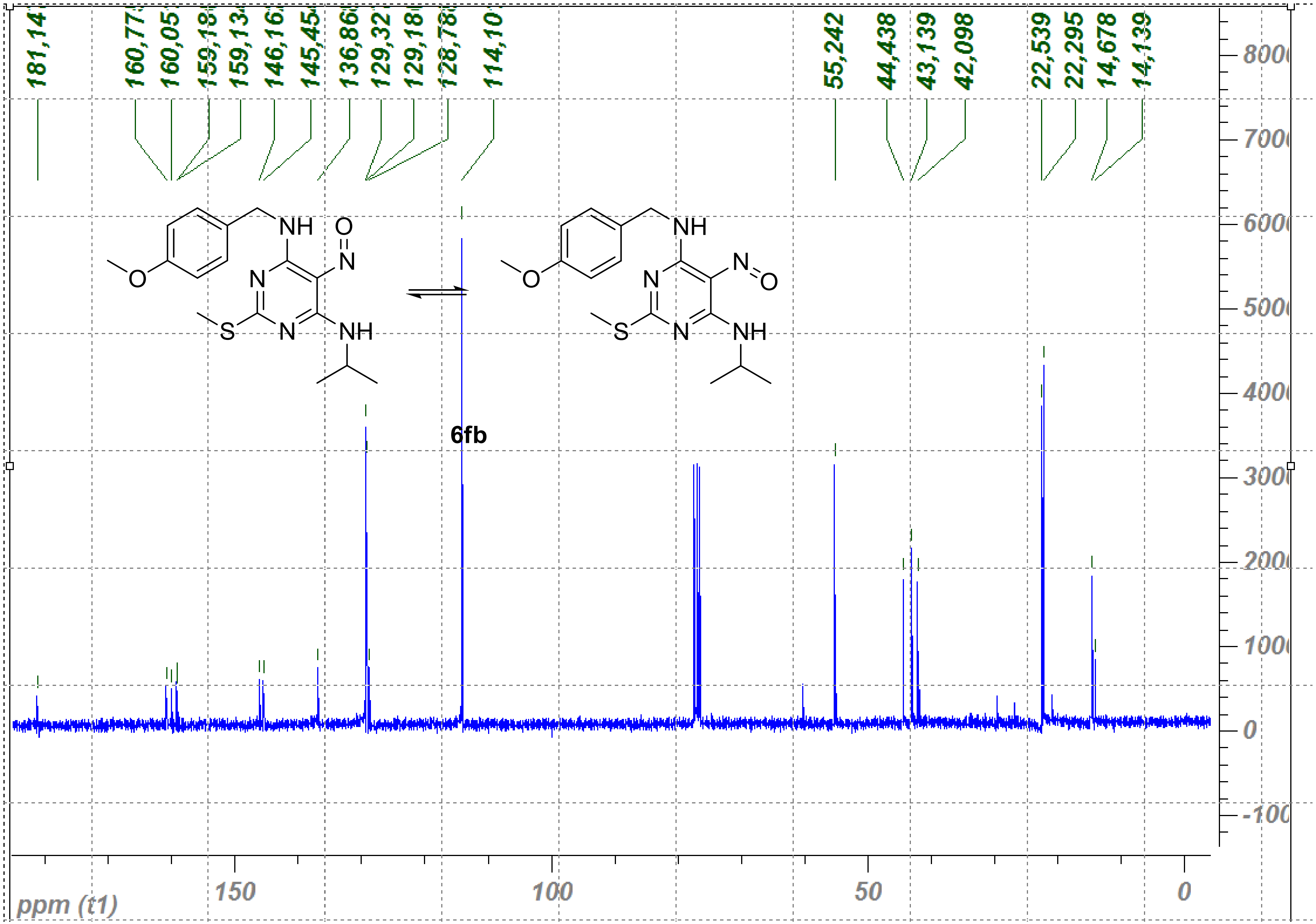
181,14
160,77
160,05
159,18
159,13
146,16
145,45
136,86
129,32
129,18
128,78
114,10
55,242
44,438
43,139
42,098
22,539
22,295
14,678
14,139
6fb
ppm (t1)
150
100
50
0

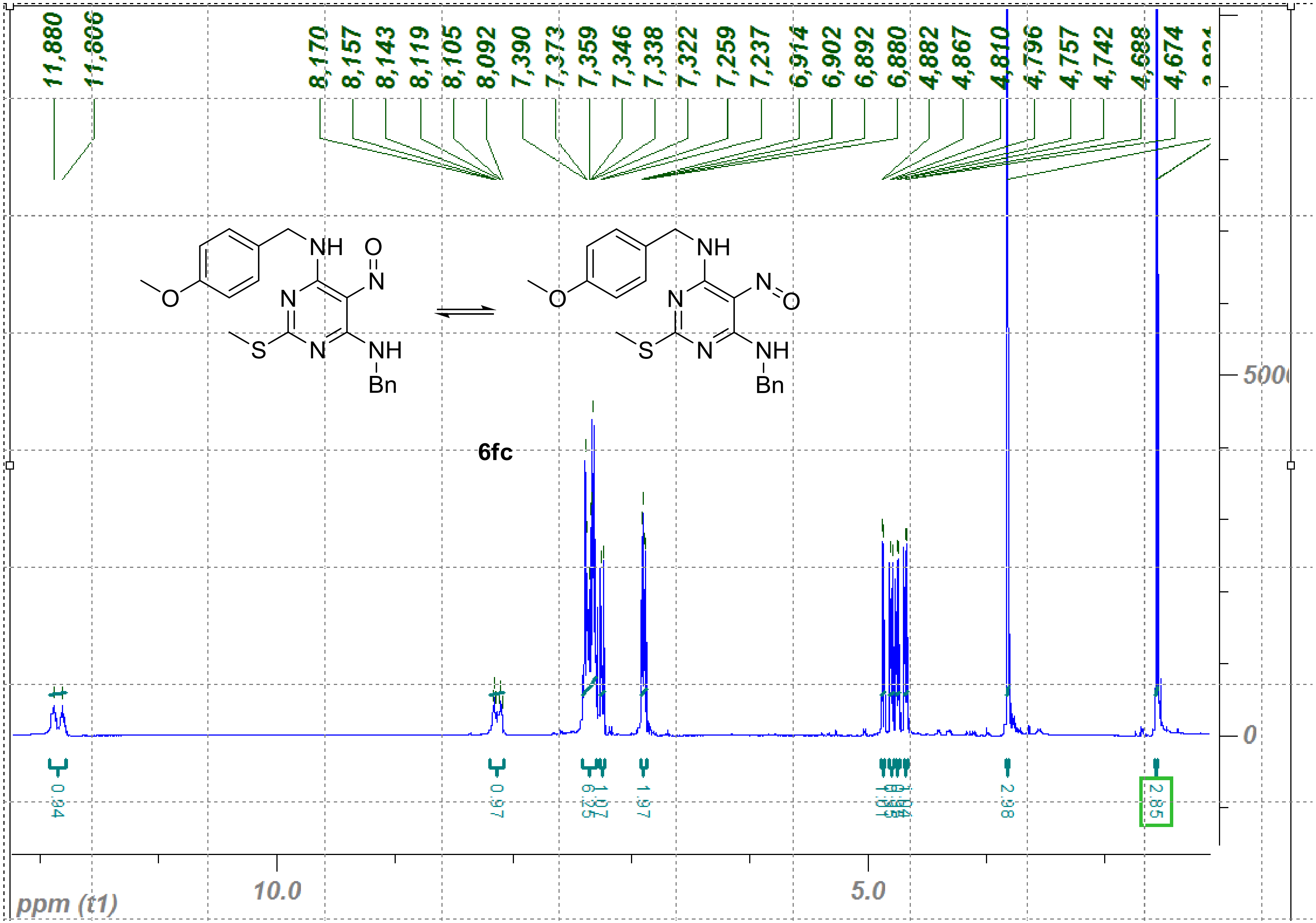
11,880
11,806
8,170
8,157
8,143
8,119
8,105
8,092
7,390
7,373
7,359
7,346
7,338
7,322
7,259
7,237
6,914
6,902
6,892
6,880
4,882
4,867
4,810
4,796
4,757
4,742
4,688
4,674
NH
O
N
N
N
S
NH
Bn
O
6fc
0.34
0.97
6.25
1.07
1.97
2.98
2.85
ppm (t1)
10.0
5.0
0

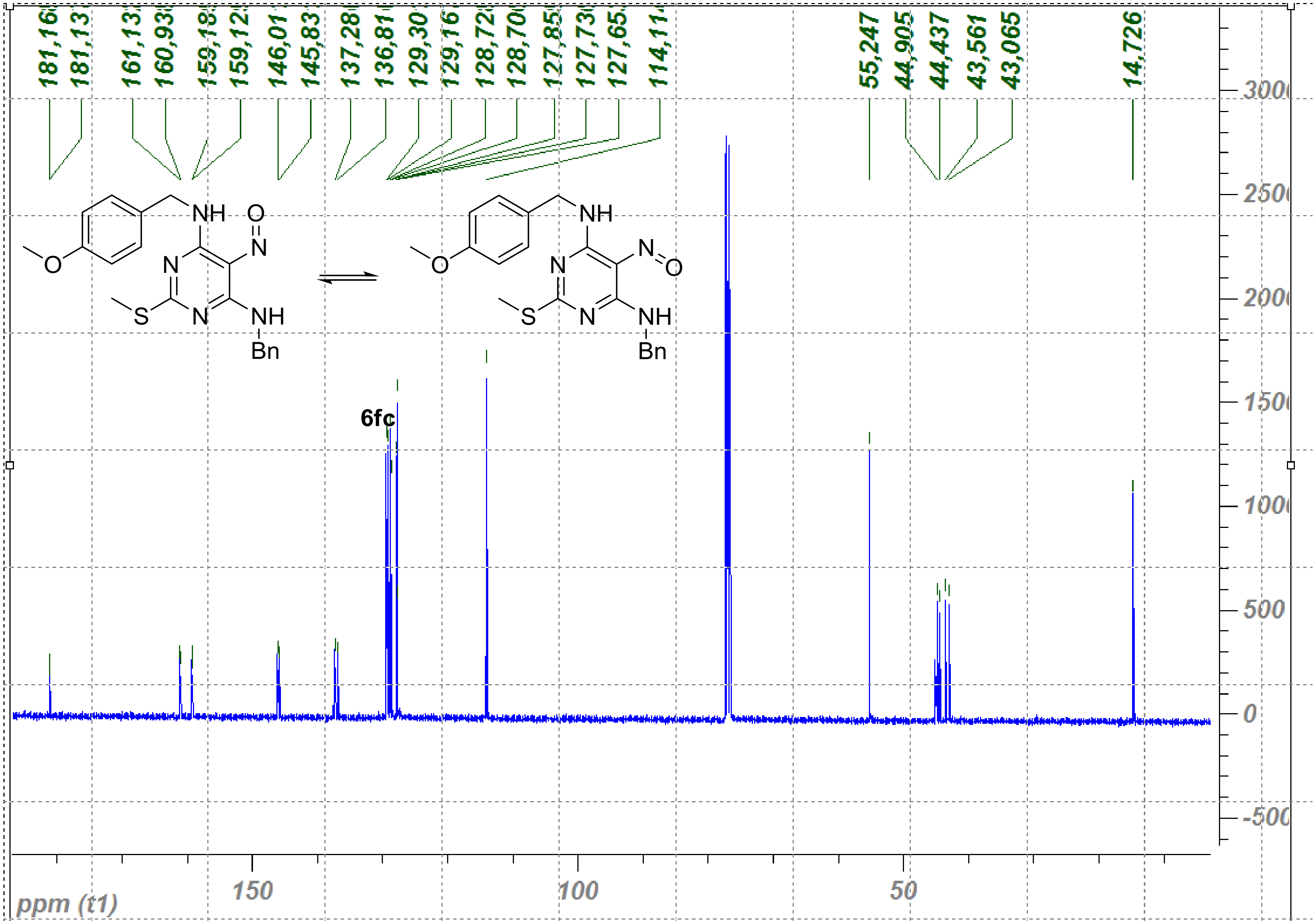
181,16
181,13
161,13
160,93
159,18
159,12
146,01
145,83
137,28
136,81
129,30
129,16
128,72
128,70
127,85
127,73
127,65
114,11
55,247
44,905
44,437
43,561
43,065
14,726
NH
O
N
O
N
S
N
NH
Bn
NH
N
O
O
N
S
N
NH
Bn
6fc
3000
2500
2000
1500
1000
500
0
-500
ppm (t1)
150
100
50

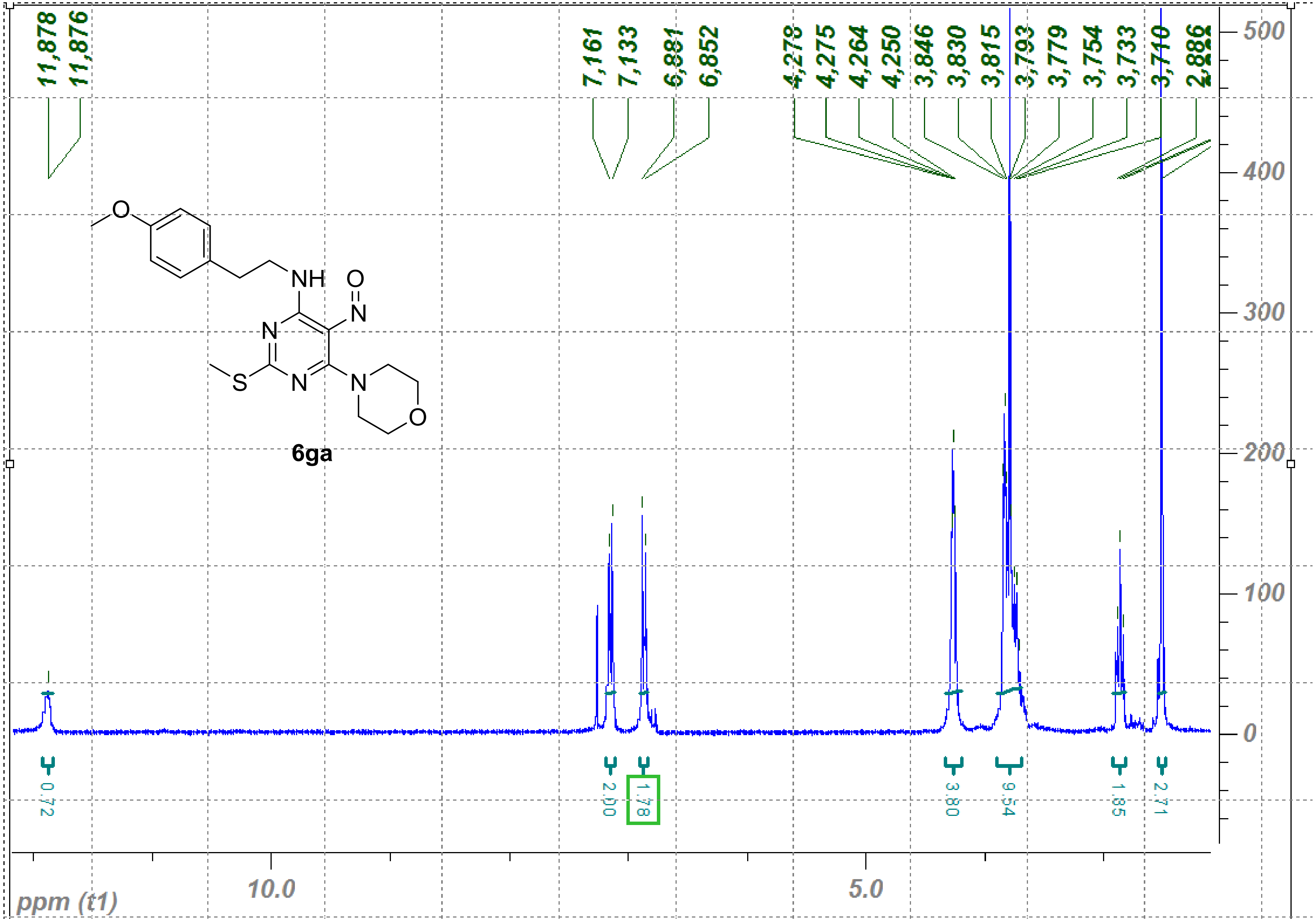
11,878
11,876
7,161
7,133
6,881
6,852
4,278
4,275
4,264
4,250
3,846
3,830
3,815
3,793
3,779
3,754
3,733
3,740
2,886
500
400
300
200
100
0
0.72
2.00
1.78
3.80
9.54
1.85
2.71
10.0
5.0
ppm (t1)
O
NH
O
N
N
N
S
N
O
6ga

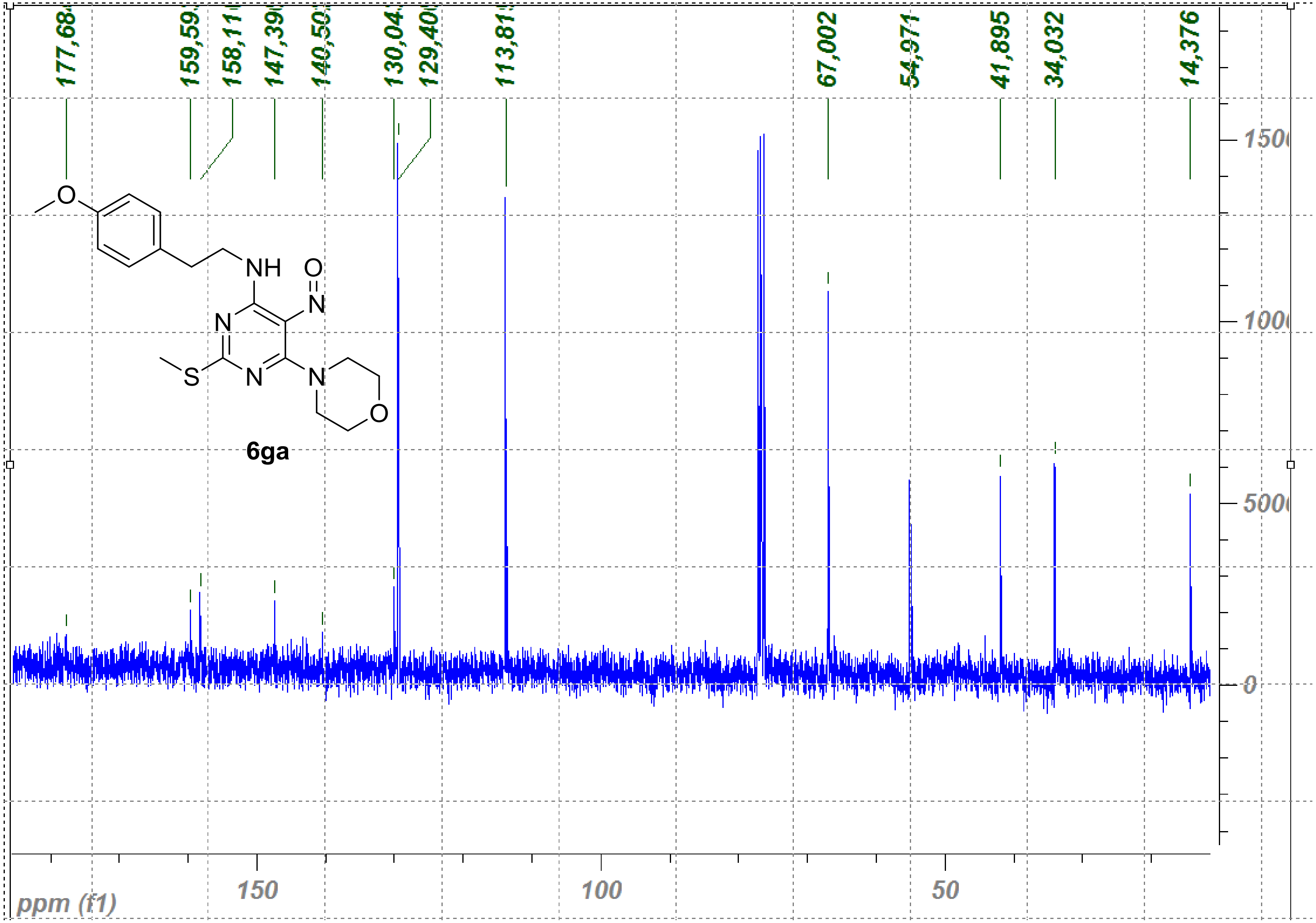
177,68
159,59
158,11
147,39
140,50
130,04
129,40
113,81
67,002
54,971
41,895
34,032
14,376
1500
1000
500
0
ppm (f1)
150
100
50
6ga

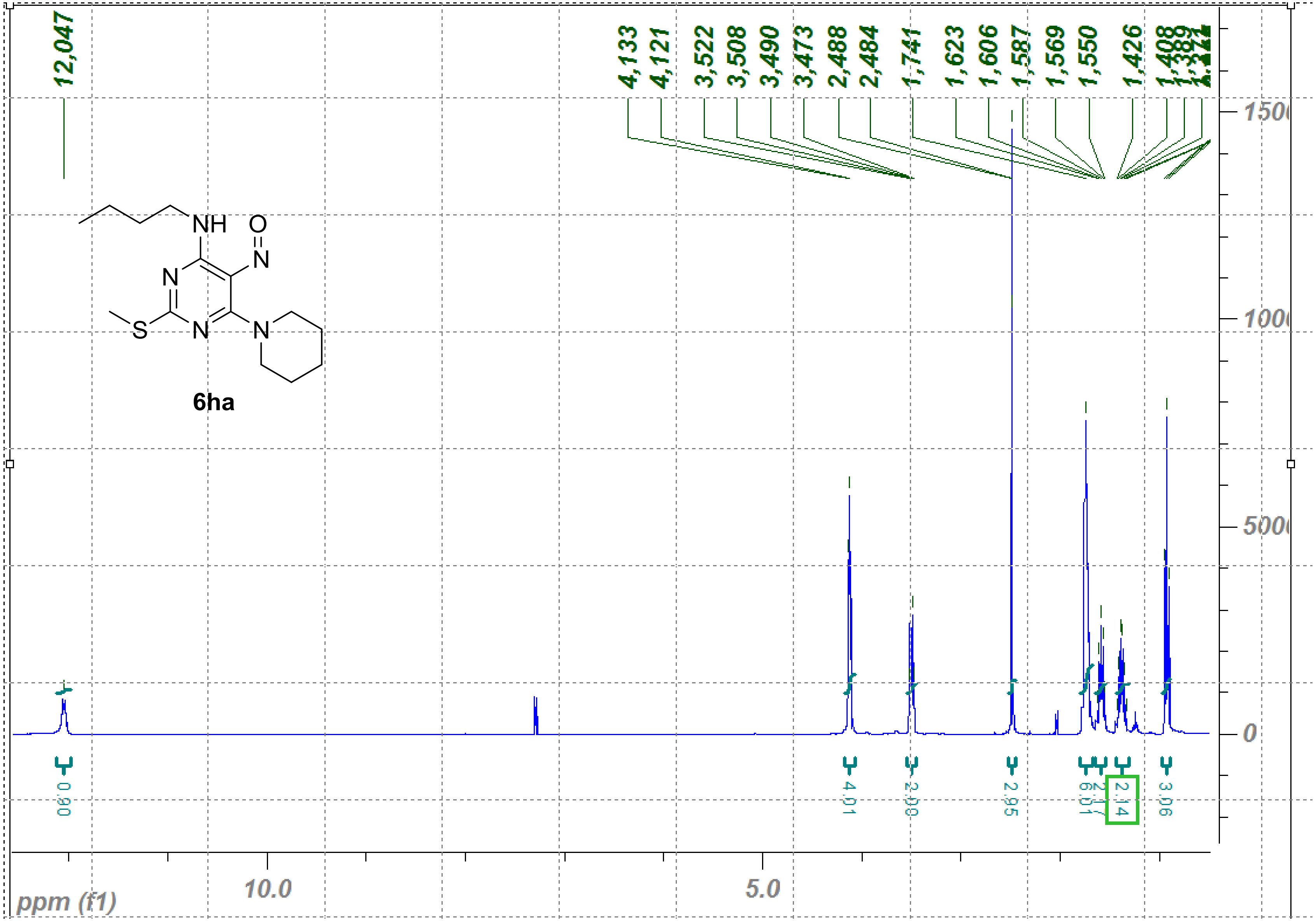

12,047
4,133
4,121
3,522
3,508
3,490
3,473
2,488
2,484
1,741
1,623
1,606
1,587
1,569
1,550
1,426
1,408
NH
O
N
N
S
N
N
6ha
0.90
4.01
2.08
2.95
6.01
2.17
2.14
3.06
ppm (f1)
10.0
5.0

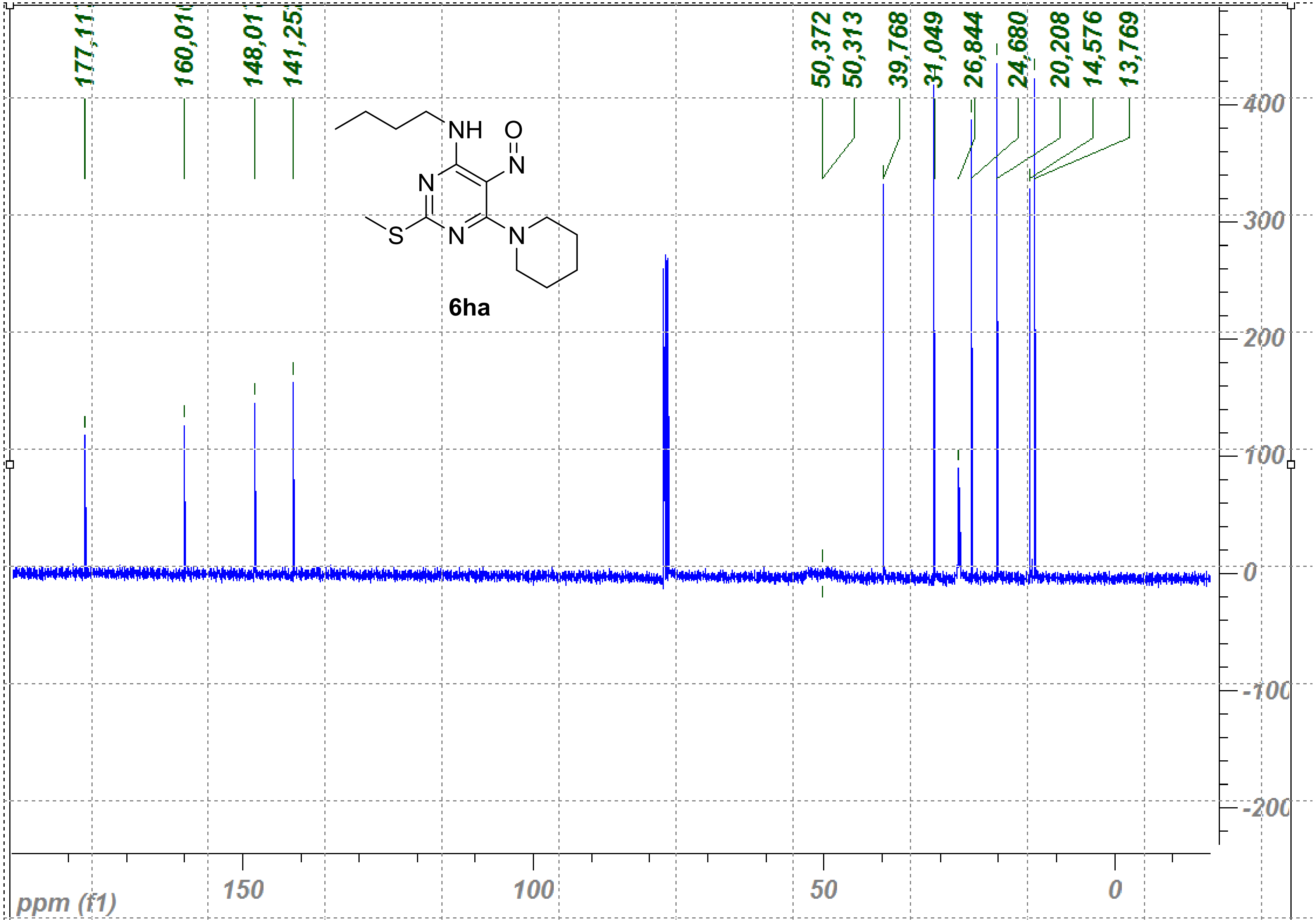
177,11
160,01
148,01
141,25
50,372
50,313
39,768
31,049
26,844
24,680
20,208
14,576
13,769
NH
O
N
N
N
S
N
6ha
400
300
200
100
0
-100
-200
ppm (f1)
150
100
50
0

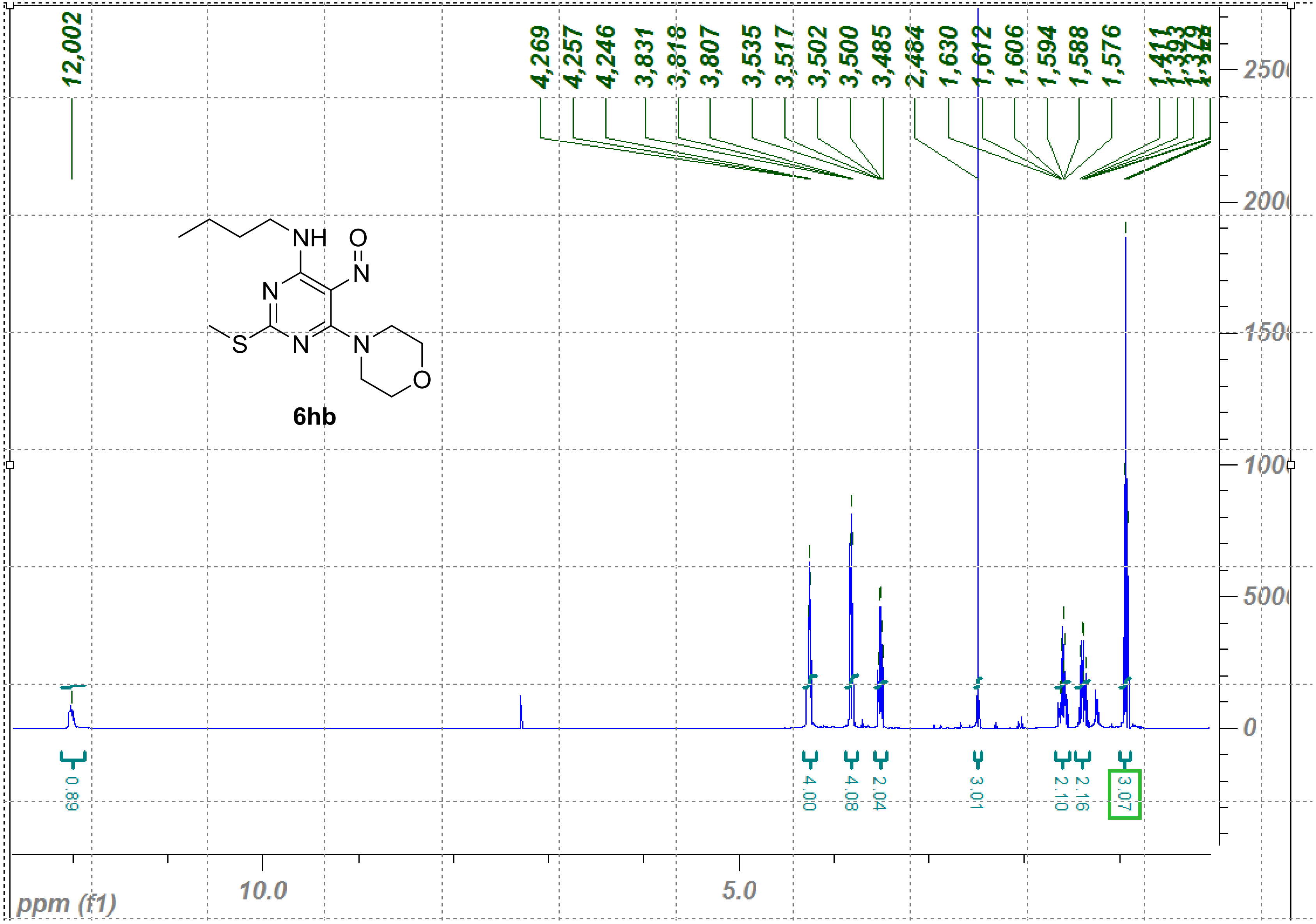
12,002
4,269
4,257
4,246
3,831
3,818
3,807
3,535
3,517
3,502
3,500
3,485
2,484
1,630
1,612
1,606
1,594
1,588
1,576
1,411
NH
O
N
N
S
N
N
O
6hb
0.89
4.00
4.08
2.04
3.01
2.10
2.16
3.07
10.0
5.0
ppm (f1)

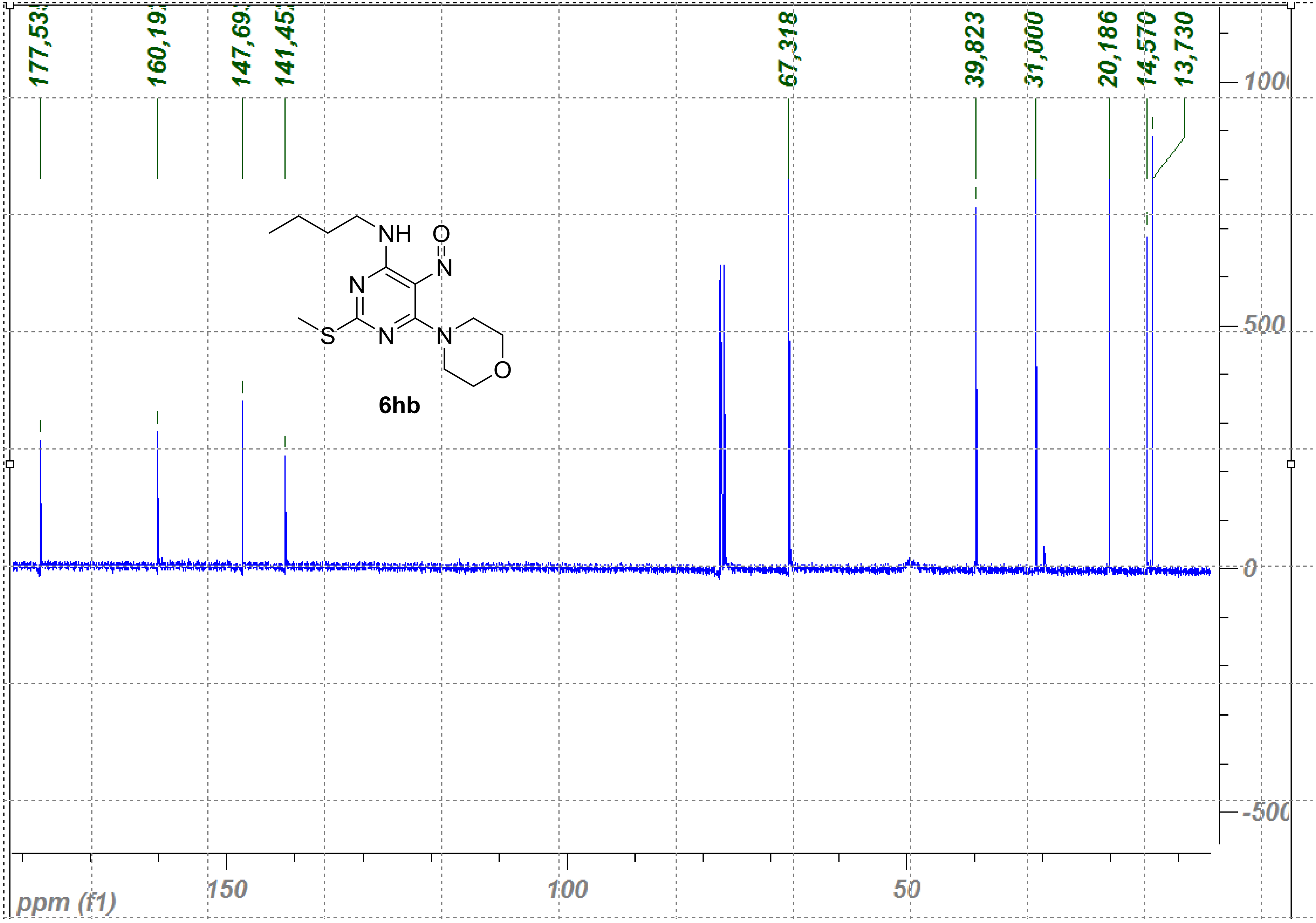
177,53
160,19
147,69
141,45
67,318
39,823
31,000
20,186
14,570
13,730
NH
O
N
N
S
N
N
O
6hb
1000
500
0
-500
ppm (f1)
150
100
50

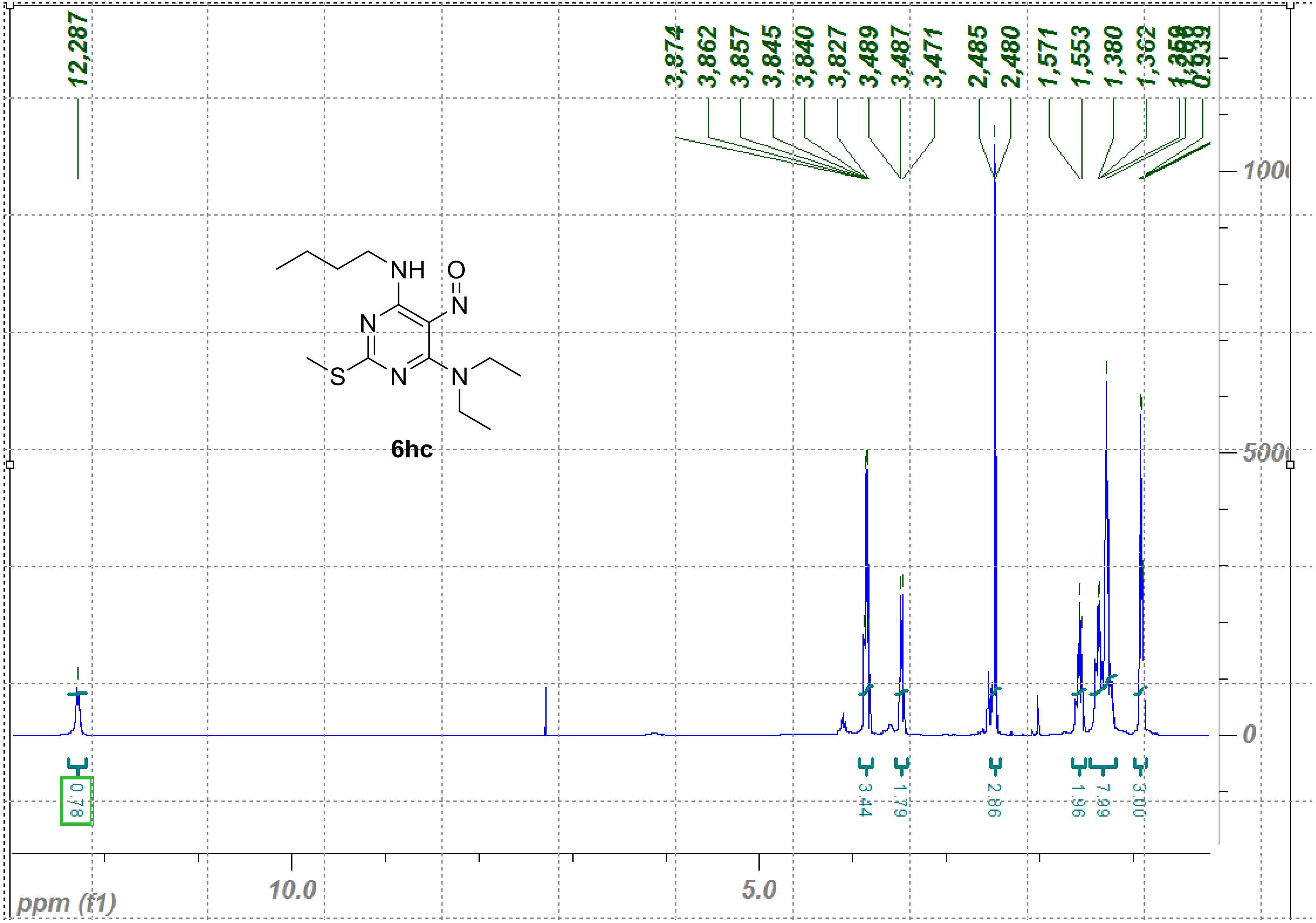

12,287
3,874
3,862
3,857
3,845
3,840
3,827
3,489
3,487
3,471
2,485
2,480
1,571
1,553
1,380
1,362
0,939
NH
O
N
N
N
S
N
6hc
0.78
3.44
1.79
2.86
1.96
7.99
3.00
10.0
5.0
ppm (f1)
0
500
100

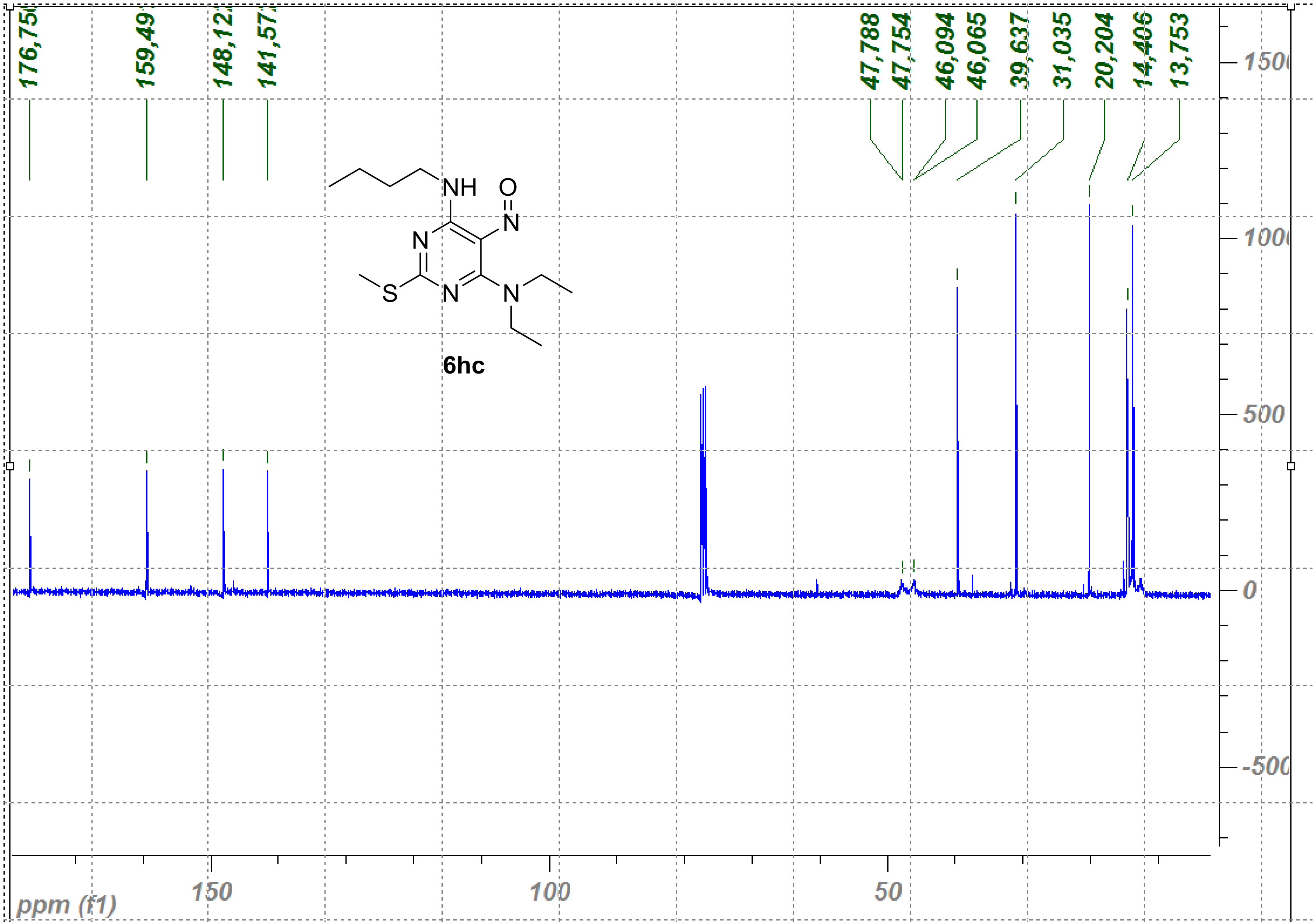
176,75
159,49
148,12
141,57
47,788
47,754
46,094
46,065
39,637
31,035
20,204
14,406
13,753
NH
O
N
N
N
S
N
6hc
1500
1000
500
0
-500
ppm (f1)
150
100
50

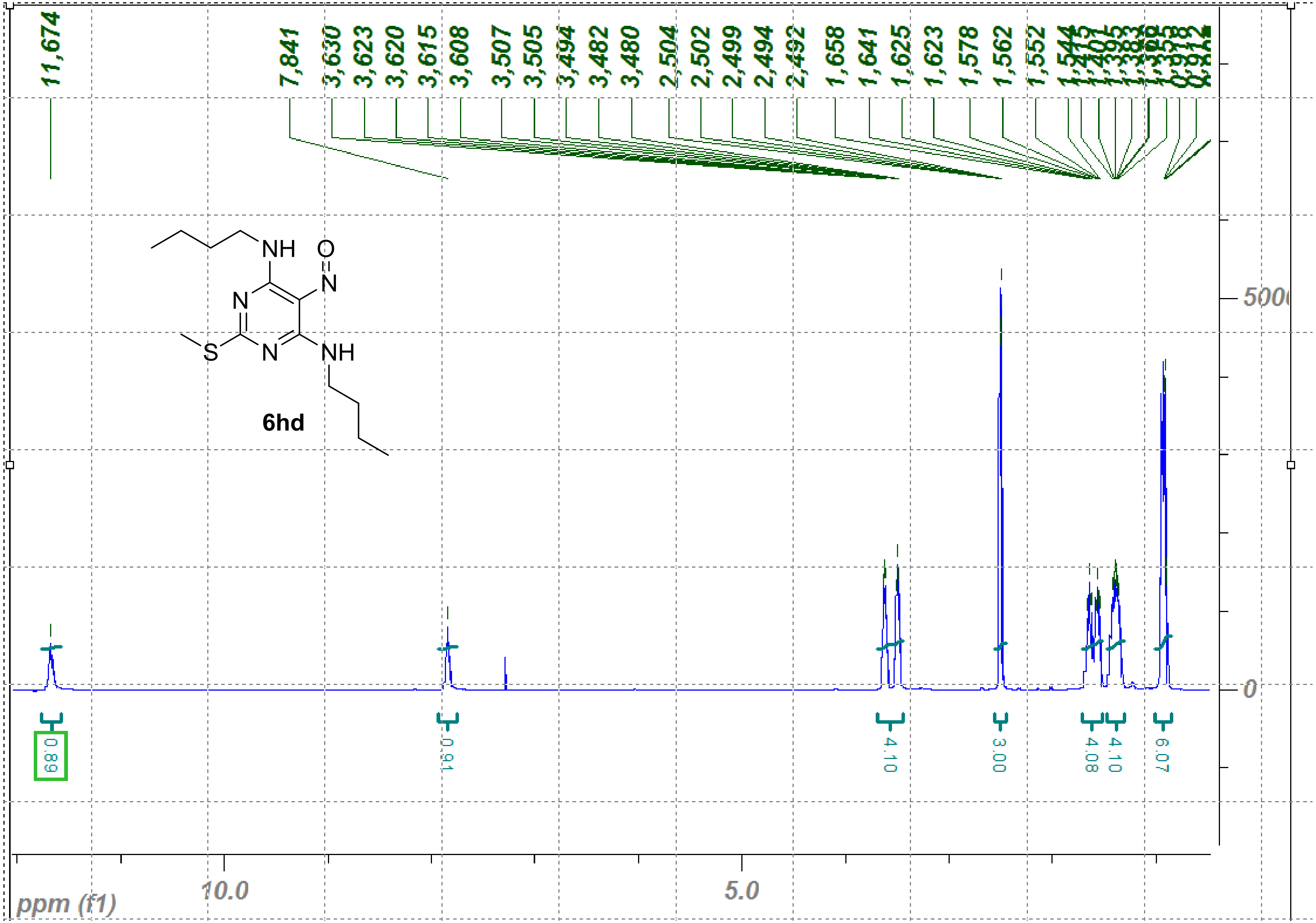
11,674
7,841
3,630
3,623
3,620
3,615
3,608
3,507
3,505
3,494
3,482
3,480
2,504
2,502
2,499
2,494
2,492
1,658
1,641
1,625
1,623
1,578
1,562
1,552
NH
O
N
N
S
N
NH
6hd
500
0
0.89
0.91
4.10
3.00
4.08
4.10
6.07
10.0
5.0
ppm (f1)

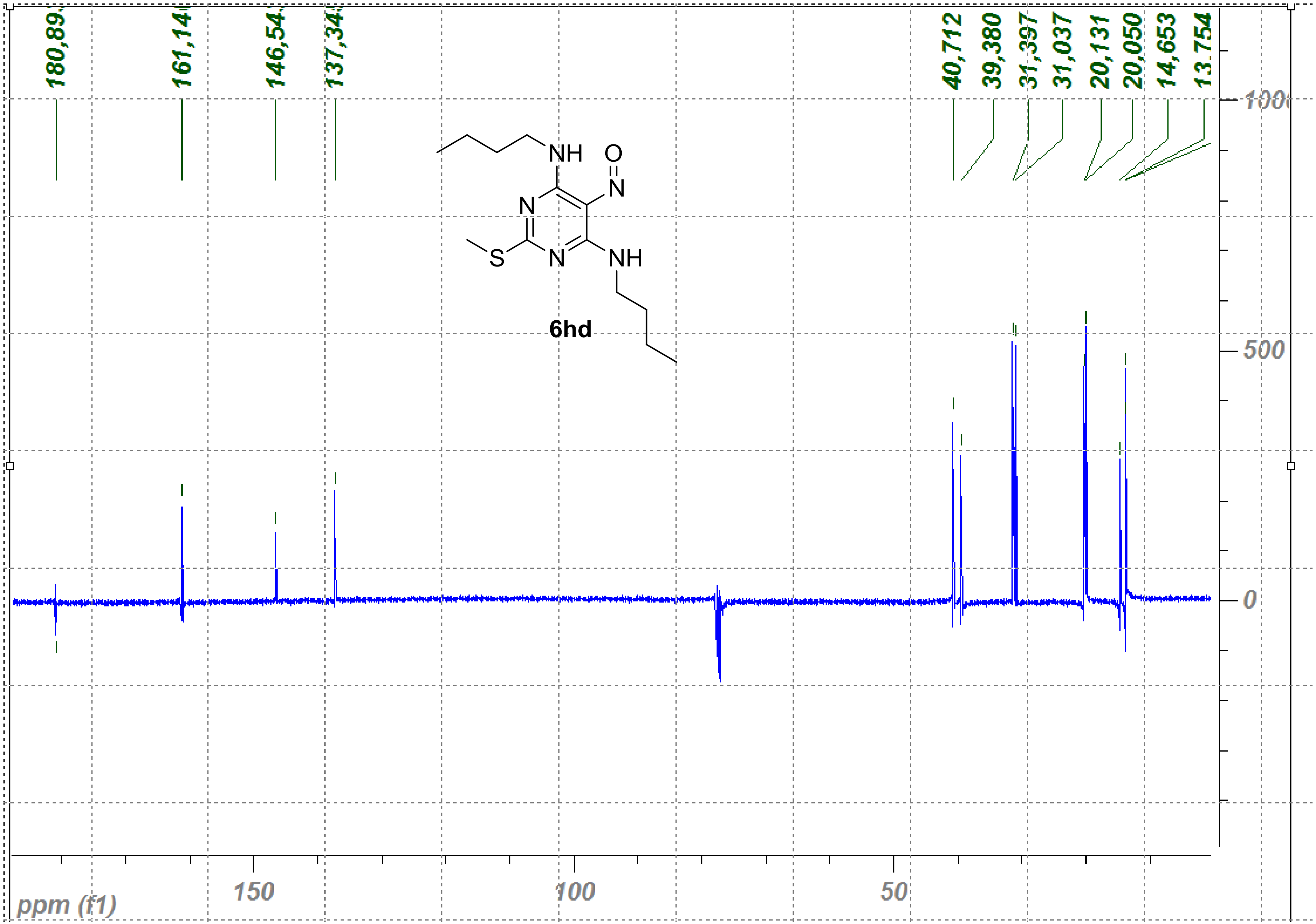

180,89
161,14
146,54
137,34
40,712
39,380
31,397
31,037
20,131
20,050
14,653
13,754
NH
O
N
N
S
N
NH
6hd
1000
500
0
ppm (f1)
150
100
50

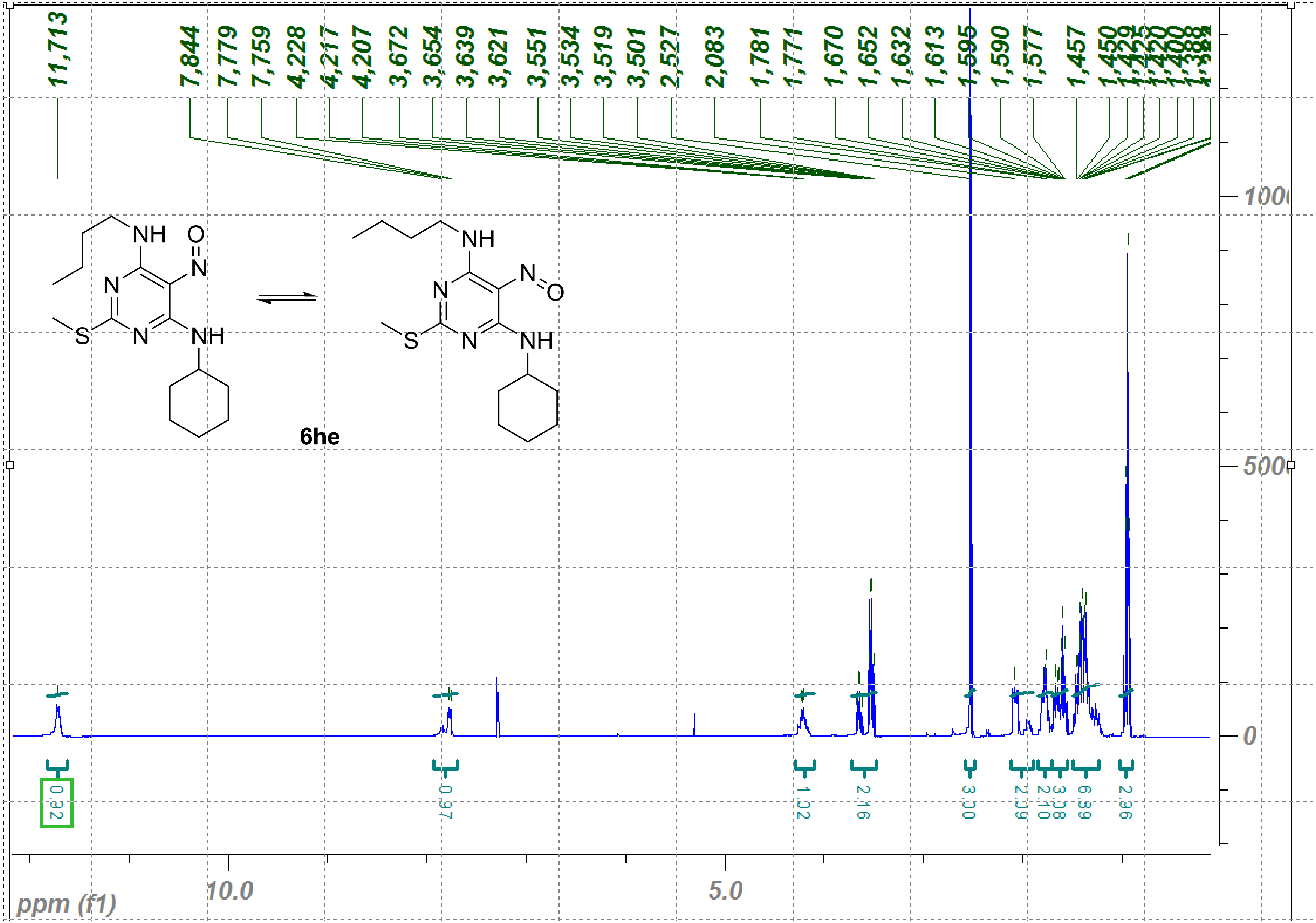

6he
11,713
7,844
7,779
7,759
4,228
4,217
4,207
3,672
3,654
3,639
3,621
3,551
3,534
3,519
3,501
2,527
2,083
1,781
1,771
1,670
1,652
1,632
1,613
1,595
1,590
1,577
1,457
1,450
1,420
1,400
1,388
0.92
0.97
1.02
2.16
3.00
2.09
2.10
3.08
6.99
2.86
ppm (f1)
10.0
5.0
1000
500
0

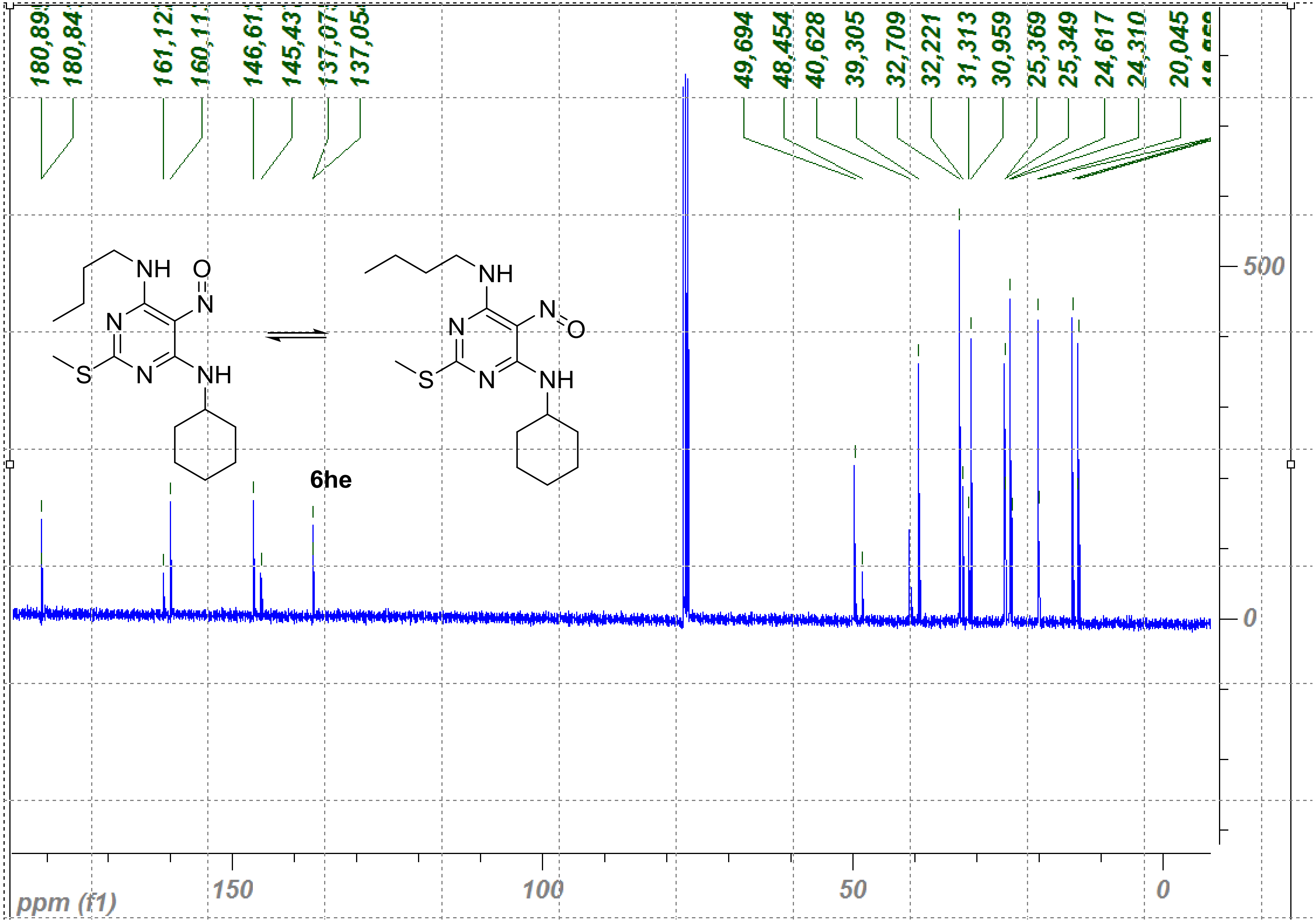

180,89
180,84
161,12
160,11
146,61
145,43
137,07
137,05
49,694
48,454
40,628
39,305
32,709
32,221
31,313
30,959
25,369
25,349
24,617
24,310
20,045
NH
O
N
N
S
N
NH
NH
N
O
N
S
N
NH
6he
500
0
150
100
50
0
ppm (f1)

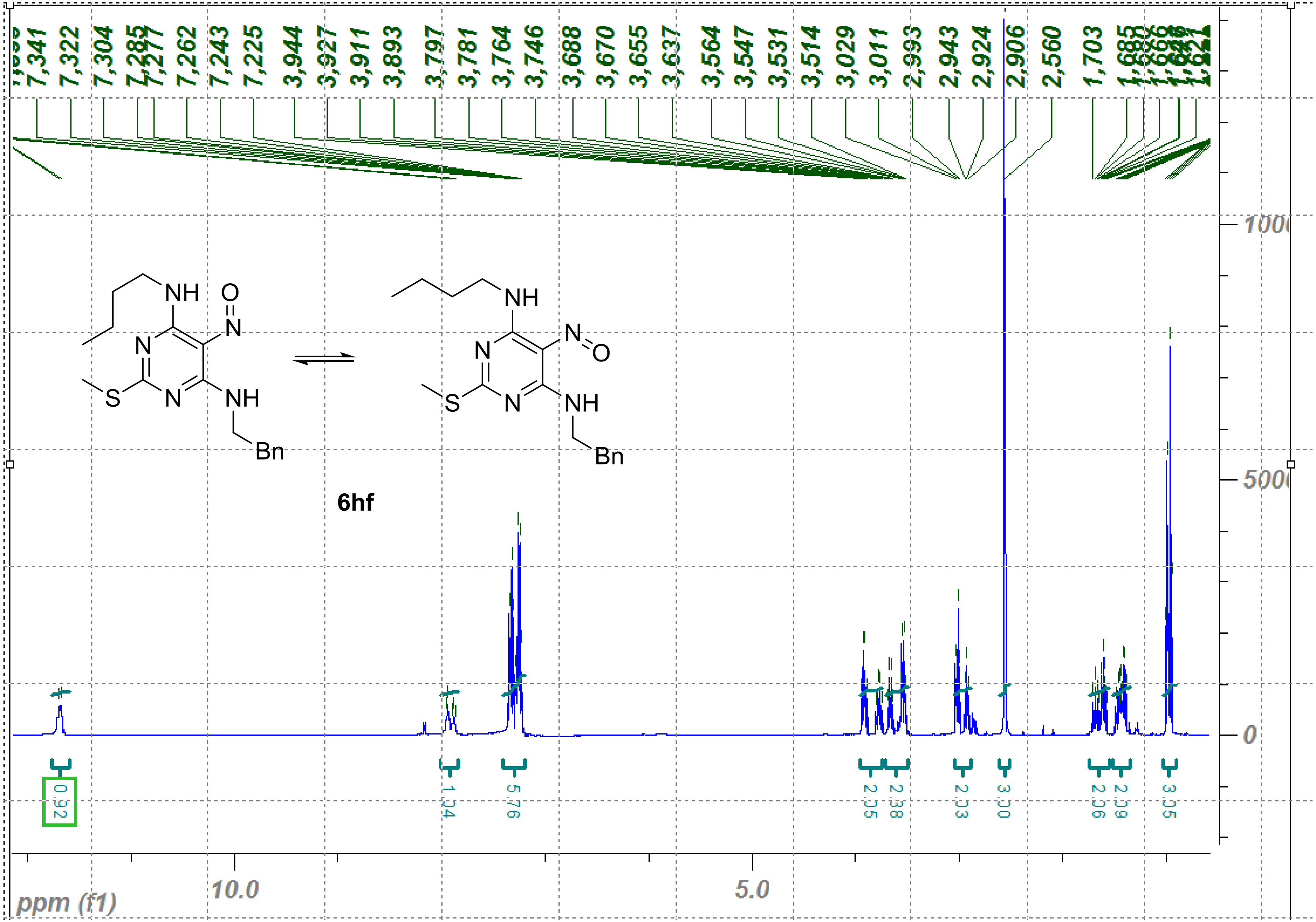
6hf
ppm (f1)
10.0
5.0
0.92
1.04
5.76
2.05
2.38
2.03
3.00
2.06
2.09
3.05

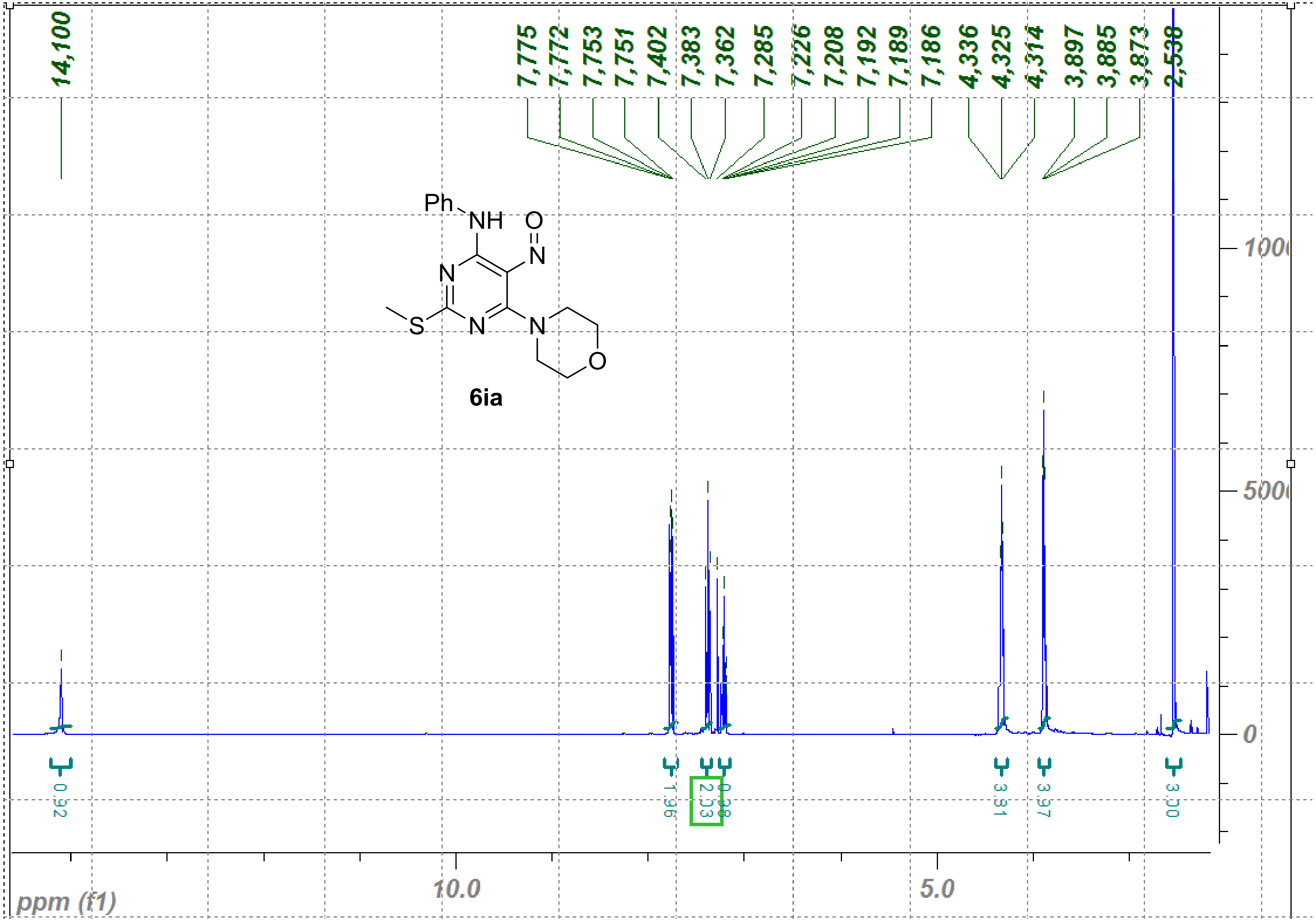
14,100
7,775
7,772
7,753
7,751
7,402
7,383
7,362
7,285
7,226
7,208
7,192
7,189
7,186
4,336
4,325
4,314
3,897
3,885
3,873
2,538
Ph
NH
O
N
N
N
S
N
O
6ia
1000
5000
0
0.92
1.96
2.03
3.81
3.97
3.00
10.0
5.0
ppm (f1)

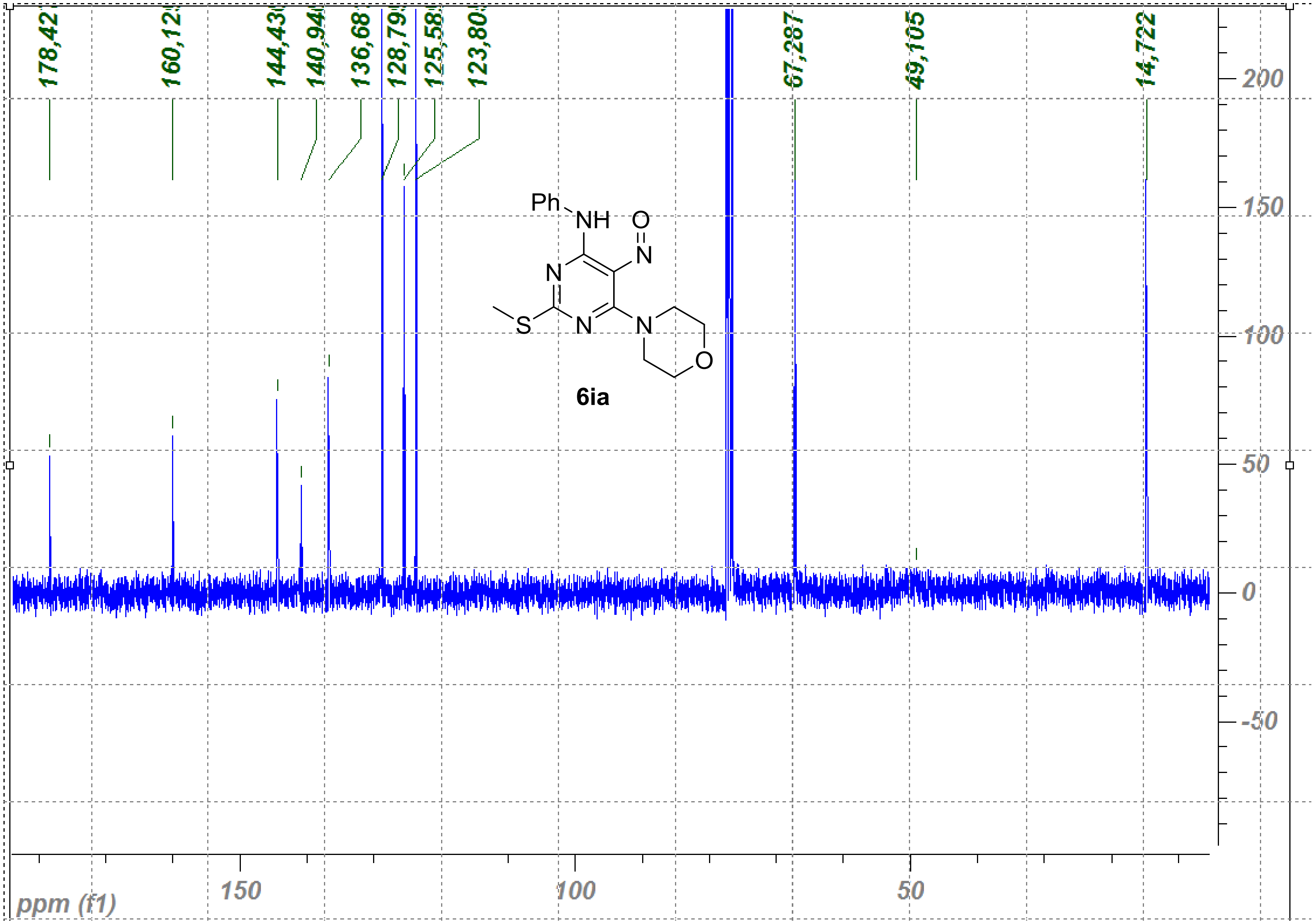

178,42
160,12
144,43
140,94
136,68
128,79
125,58
123,80
67,287
49,105
14,722
Ph
NH
O
N
N
S
N
N
O
6ia
200
150
100
50
0
-50
150
100
50
ppm (f1)

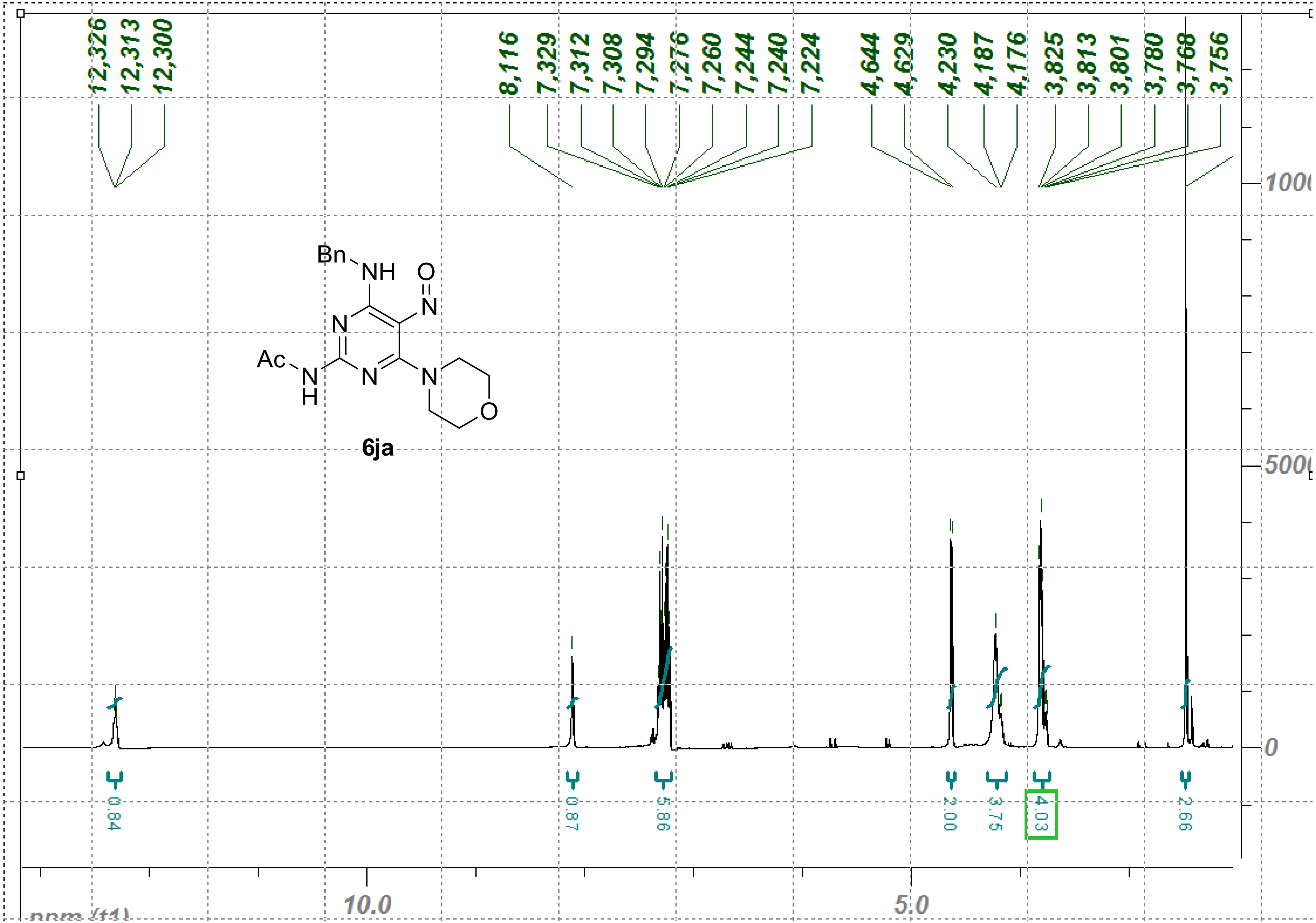
12,326
12,313
12,300
8,116
7,329
7,312
7,308
7,294
7,276
7,260
7,244
7,240
7,224
4,644
4,629
4,230
4,187
4,176
3,825
3,813
3,801
3,780
3,768
3,756
Bn
NH
O
N
N
Ac
N
H
N
N
O
6ja
0.84
0.87
5.86
2.00
3.75
4.03
2.66
10.0
5.0
ppm (t1)

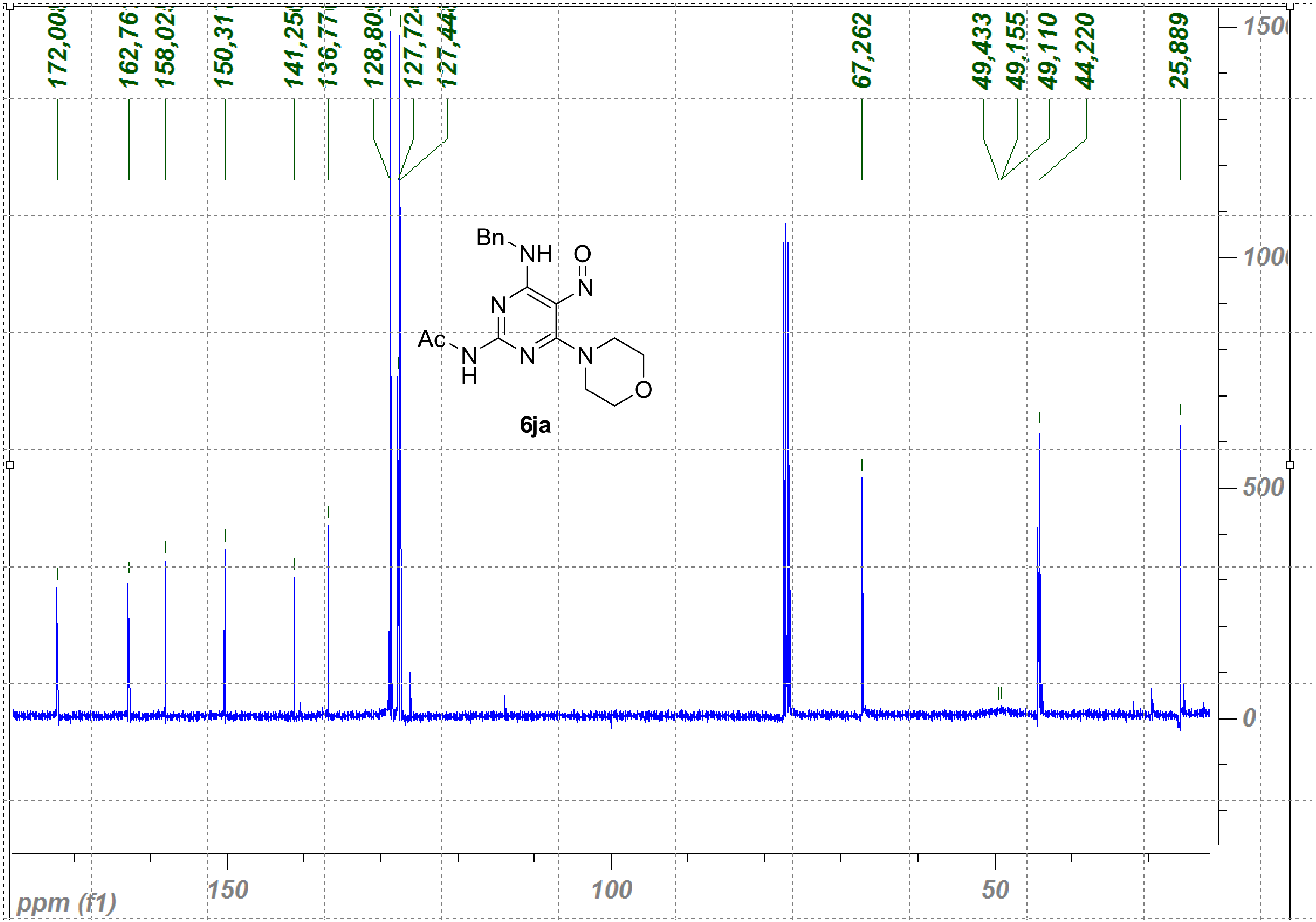
172,00
162,76
158,02
150,31
141,25
136,77
128,80
127,72
127,44
67,262
49,433
49,155
49,110
44,220
25,889
Bn
NH
O
N
N
Ac
N
H
N
N
O
6ja
1500
1000
500
0
ppm (f1)
150
100
50

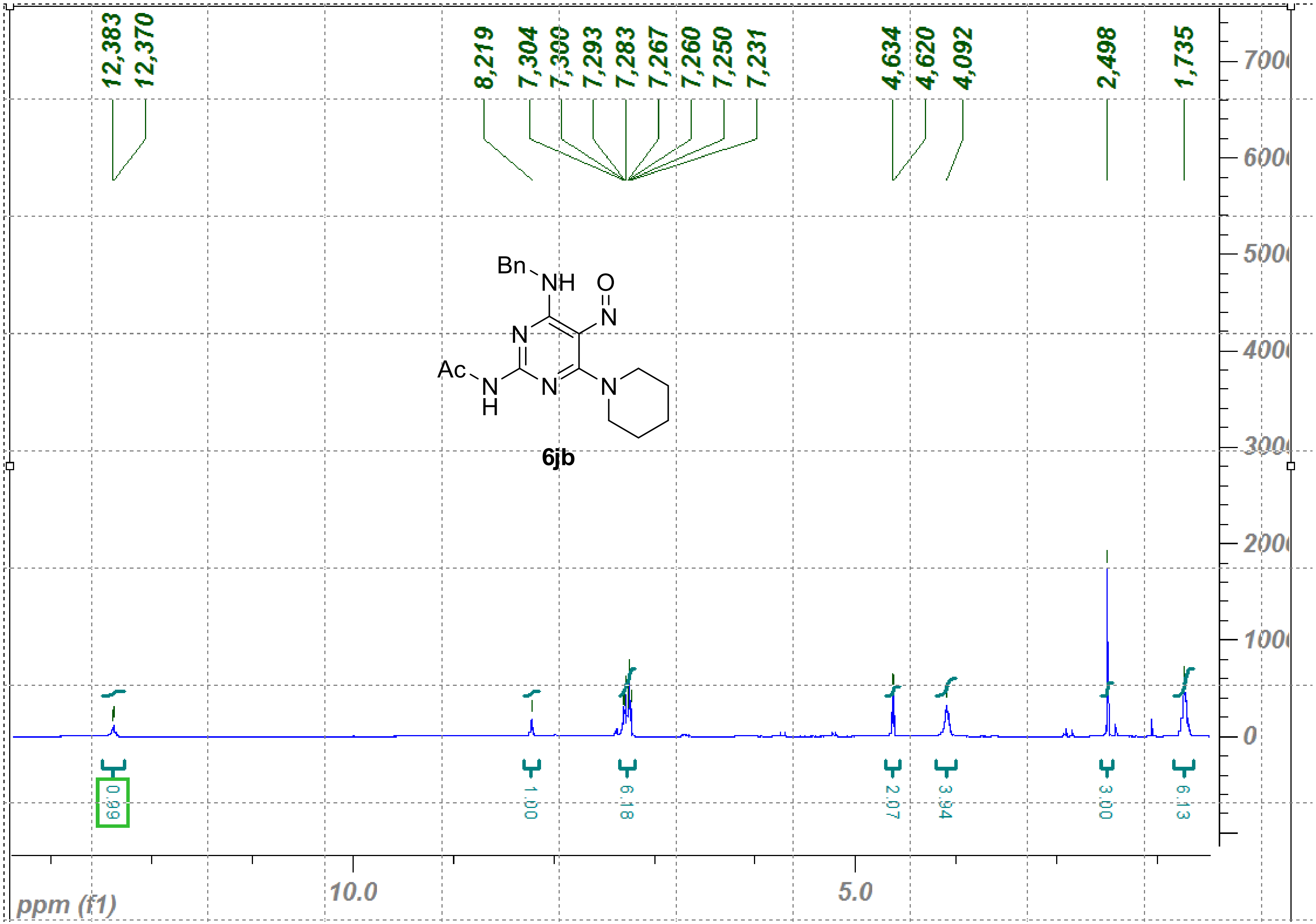
12,383
12,370
8,219
7,304
7,300
7,293
7,283
7,267
7,260
7,250
7,231
4,634
4,620
4,092
2,498
1,735
Bn
NH
O
N
N
Ac
N
H
N
N
6jb
0.99
1.00
6.18
2.07
3.94
3.00
6.13
10.0
5.0
ppm (f1)

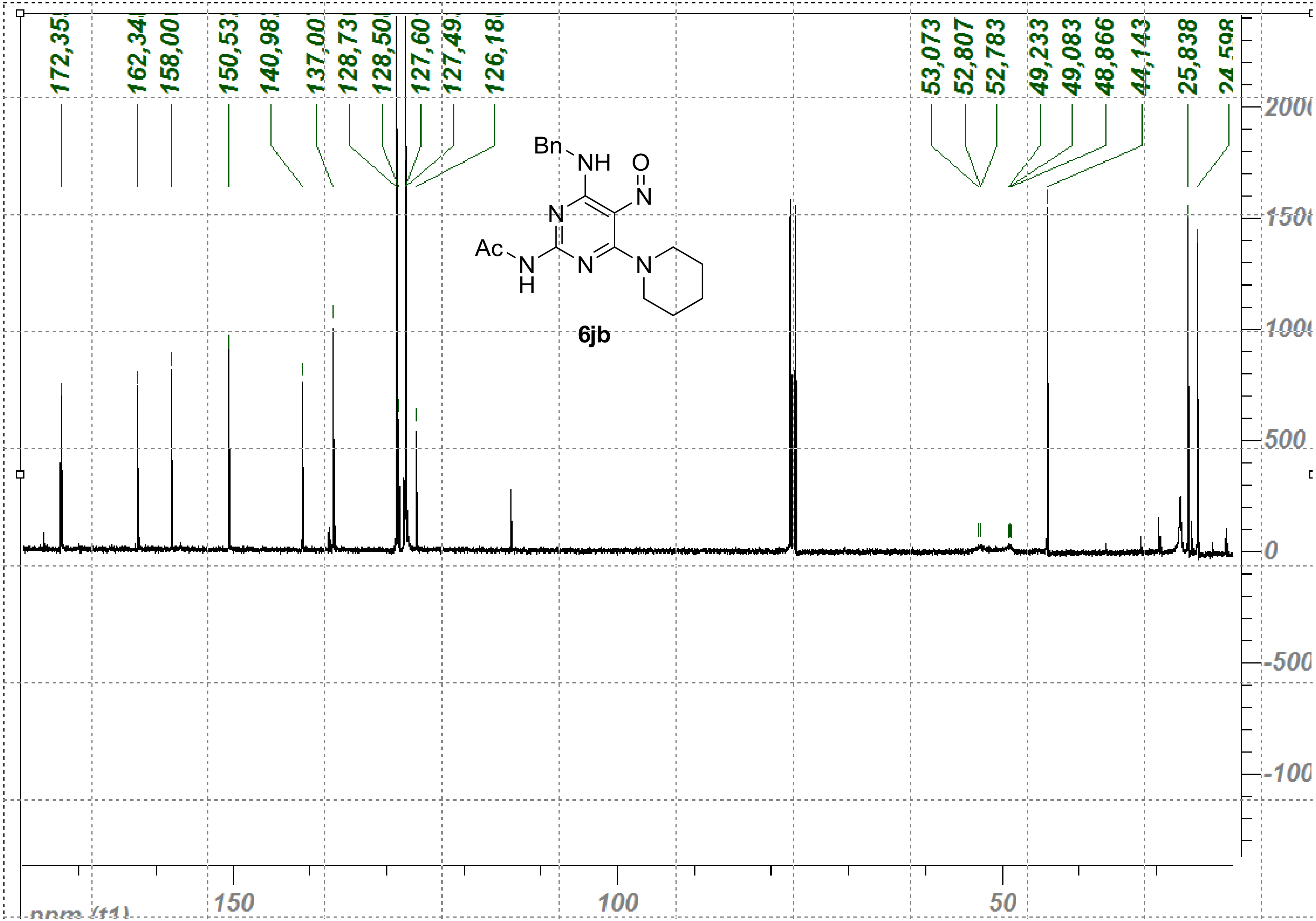
172,35
162,34
158,00
150,53
140,98
137,00
128,73
128,50
127,60
127,49
126,18
53,073
52,807
52,783
49,233
49,083
48,866
44,143
25,838
24,598
Bn
NH
O
N
N
Ac
N
H
N
N
6jb
2000
1500
1000
500
0
-500
-100
150
100
50
ppm (t1)

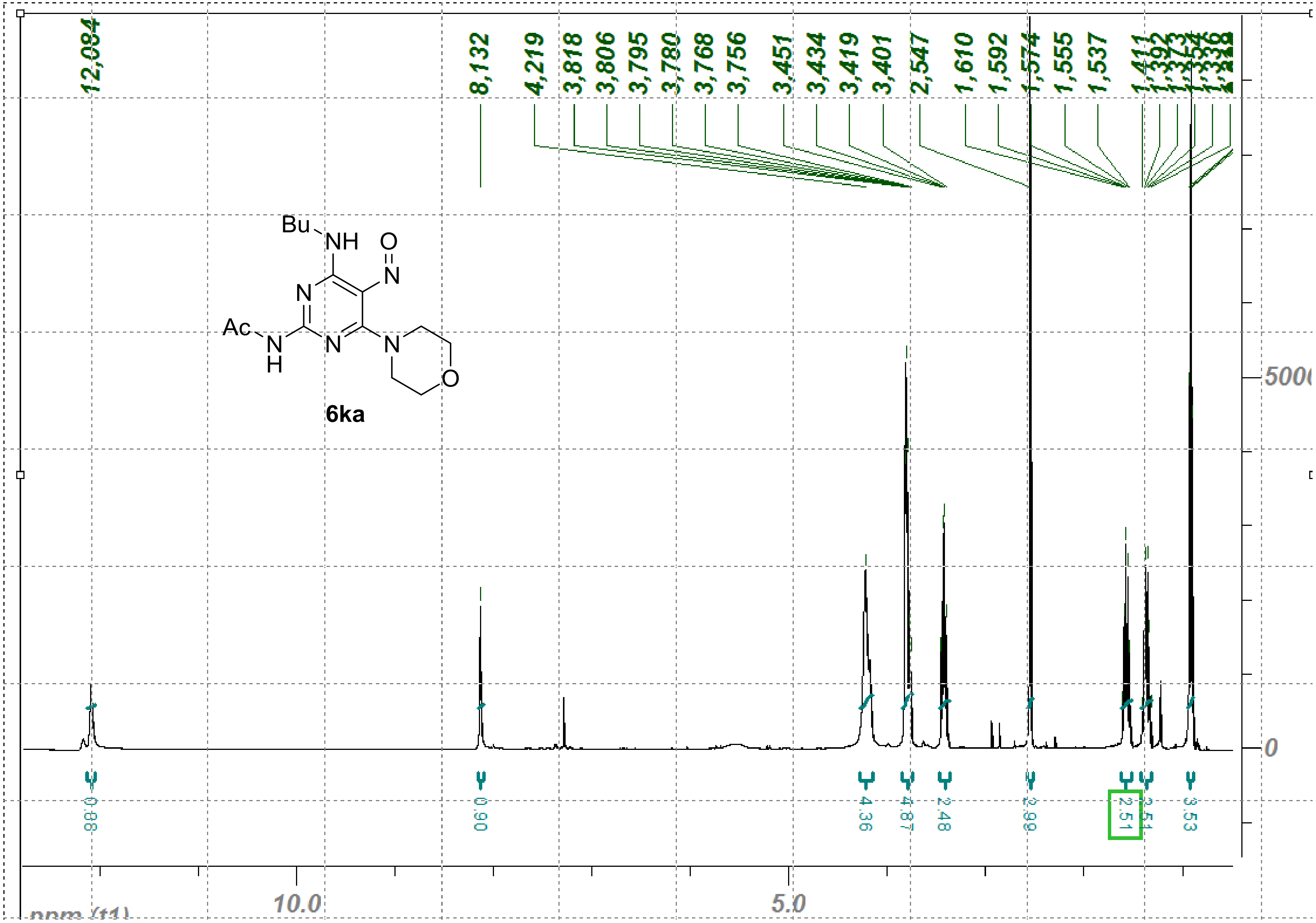

12,084
8,132
4,219
3,818
3,806
3,795
3,780
3,768
3,756
3,451
3,434
3,419
3,401
2,547
1,610
1,592
1,574
1,555
1,537
1,411
Bu
NH
O
N
N
Ac
N
H
N
N
O
6ka
5000
0
0.88
0.90
4.36
4.87
2.48
2.99
2.51
3.53
10.0
5.0
ppm (t1)

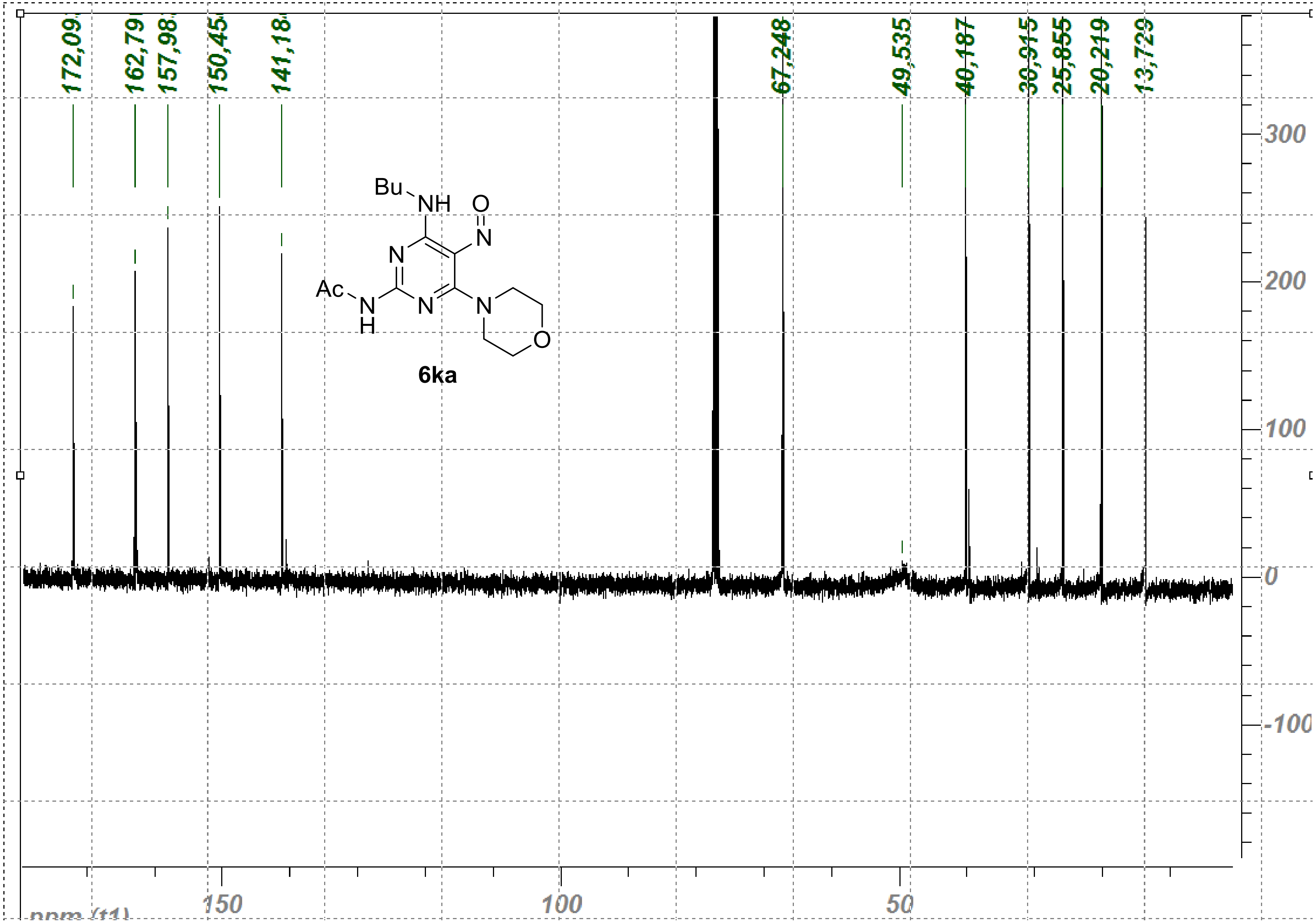
172,09
162,79
157,98
150,45
141,18
67,248
49,535
40,187
30,915
25,855
20,219
13,729
300
200
100
0
-100
150
100
50
ppm (t1)
Bu
NH
O
N
N
Ac
N
H
N
N
O
6ka

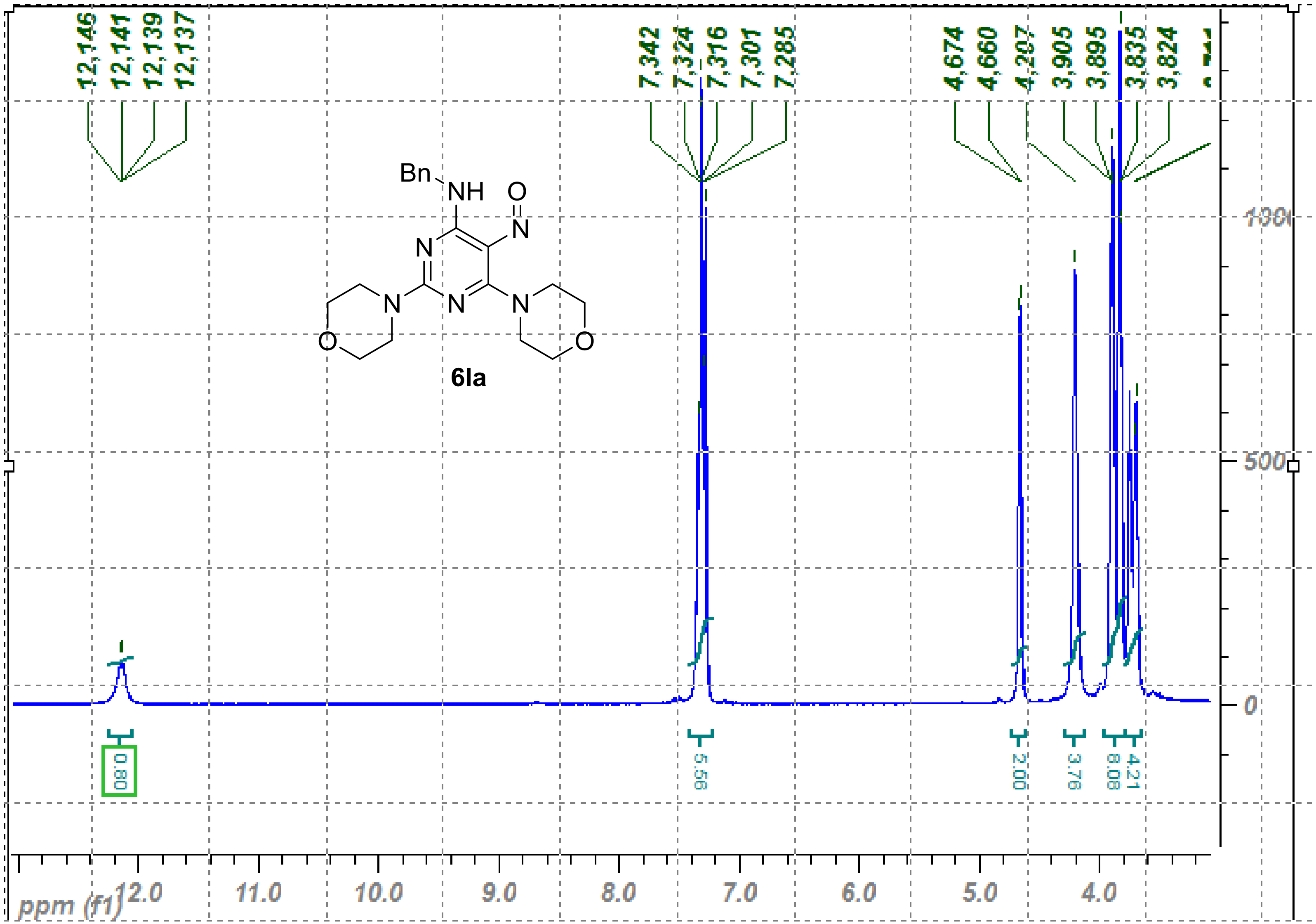
12,146
12,141
12,139
12,137
7,342
7,324
7,316
7,301
7,285
4,674
4,660
4,207
3,905
3,895
3,835
3,824
Bn
NH
O
N
N
N
N
N
O
O
6la
1000
500
0
0.80
5.56
2.00
3.76
8.08
4.21
ppm (f1)
12.0
11.0
10.0
9.0
8.0
7.0
6.0
5.0
4.0

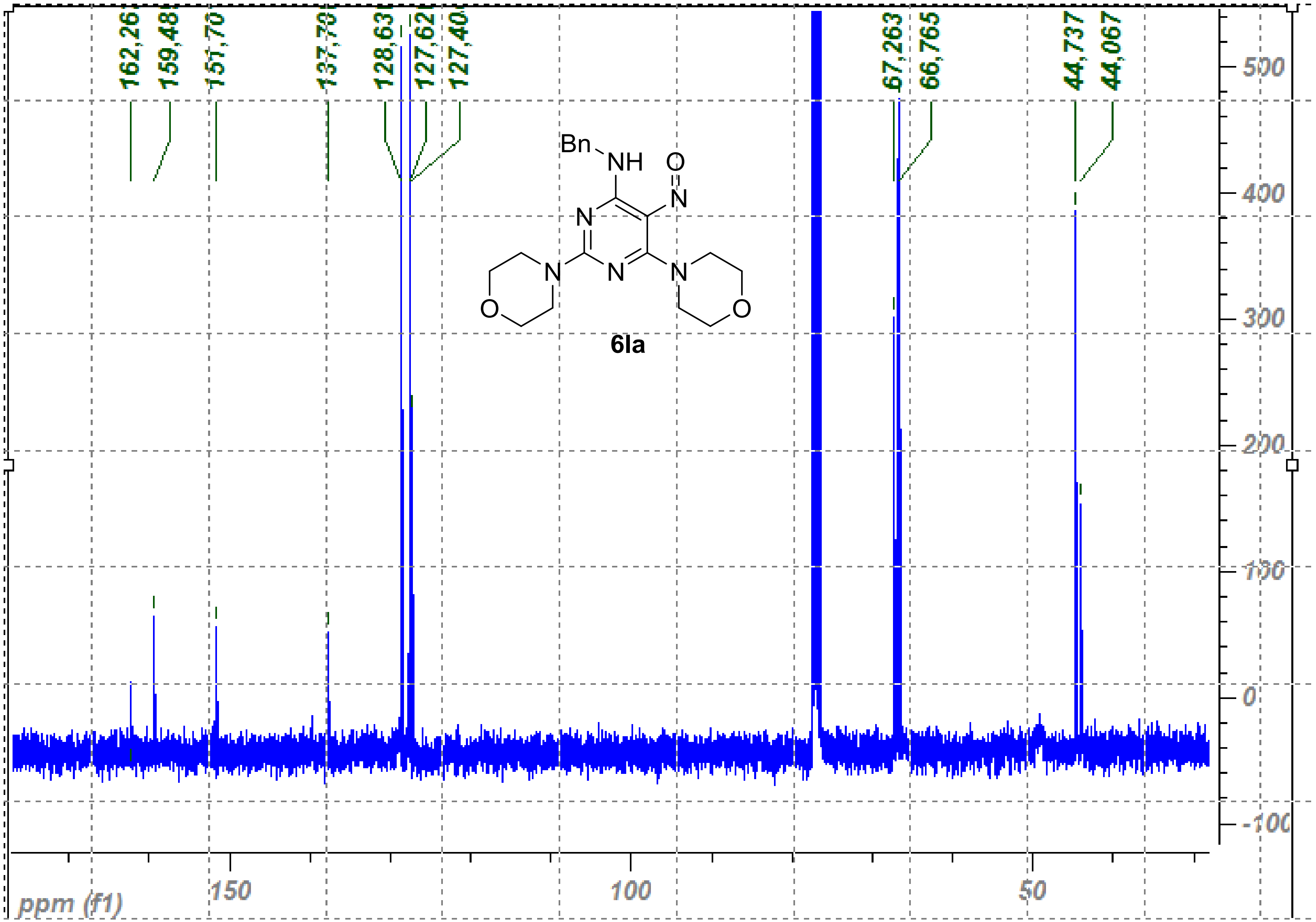
162,26
159,48
151,70
137,70
128,63
127,62
127,40
67,263
66,765
44,737
44,067
Bn
NH
O
N
N
N
N
N
O
O
6la
ppm (f1)
150
100
50
500
400
300
200
100
0
-100

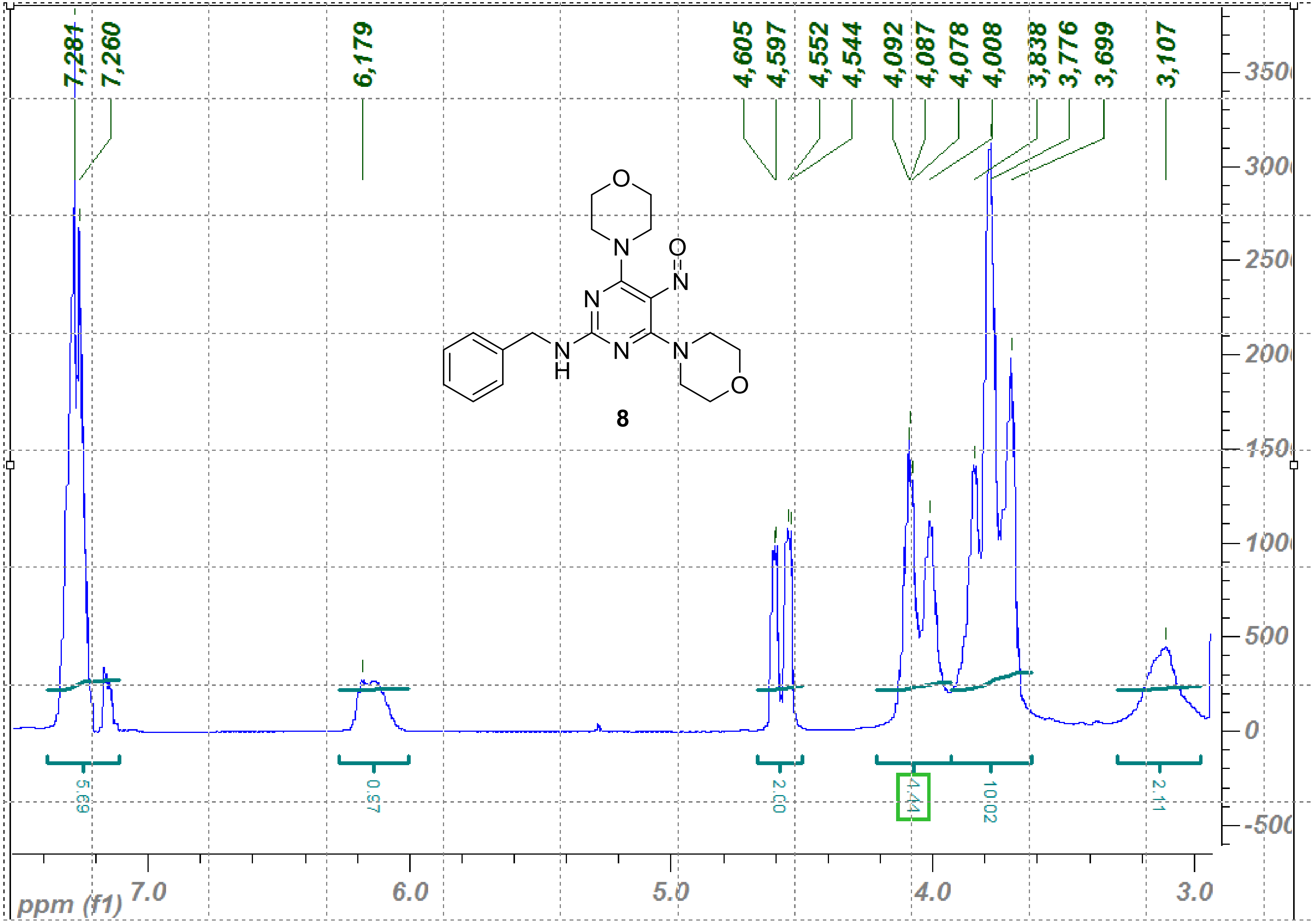

7,281
7,260
6,179
4,605
4,597
4,552
4,544
4,092
4,087
4,078
4,008
3,838
3,776
3,699
3,107
8
5.69
0.97
2.00
4.44
10.02
2.11
ppm (f1)
7.0
6.0
5.0
4.0
3.0

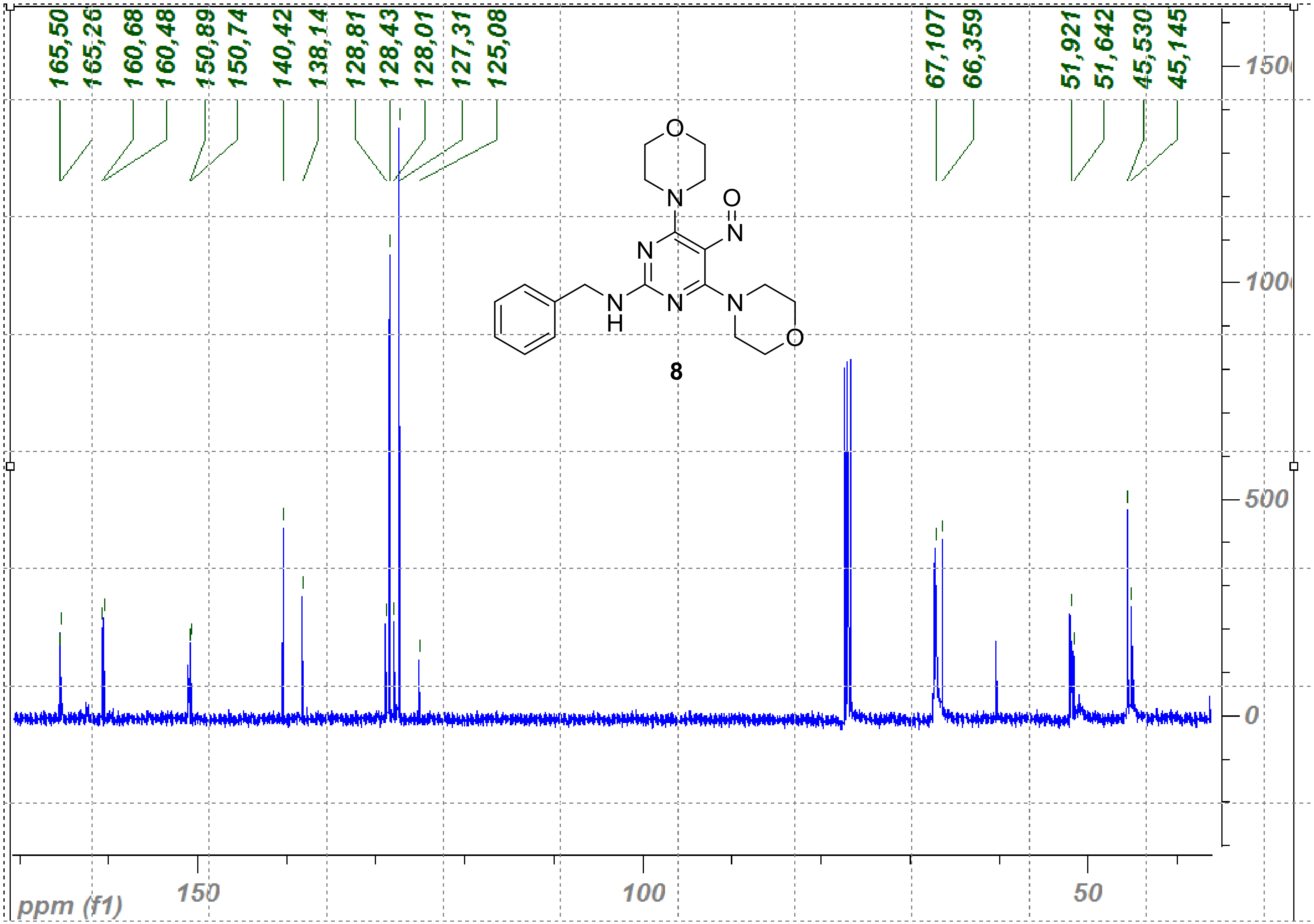
165,50
165,26
160,68
160,48
150,89
150,74
140,42
138,14
128,81
128,43
128,01
127,31
125,08
67,107
66,359
51,921
51,642
45,530
45,145
8
1500
1000
500
0
ppm (f1)
150
100
50
